# Supplementary material for: Mapping Pu’er tea plantations from GF-1 images using Object-Oriented Image Analysis (OOIA) and Support Vector Machine (SVM)
Source: PLoS One. 2023 Feb 7;18(2):e0263969. doi: 10.1371/journal.pone.0263969 (PMC9904455; doi:10.1371/journal.pone.0263969)
Supplement: Attachment — Submitted filename: PONE-D-21-11402_reviewed.pdf [file pone.0263969.s002.pdf]

PLOS ONE

Mapping Pu'er tea plantations from GF-1 images using Object-oriented image analysis (OOIA) andclassification and regression tree (CART)

--Manuscript Draft--

**Manuscript Number:**	PONE-D-21-11402
**Article Type:**	Research Article
**Full Title:**	Mapping Pu'er tea plantations from GF-1 images using Object-oriented image analysis (OOIA) andclassification and regression tree (CART)
**Short Title:**	Mapping Pu'er tea plantations from GF-1 images using OOIA and CART
**Corresponding Author:**	Jinliang Wang, Ph.D.<br>Yunnan Normal University<br>Kunming, Yunnan CHINA
**Keywords:**	Pu'er tea, OOIA, Feature selection, Decision tree, Change detection
**Abstract:**	Tea is the most popular drink worldwide, and China is the largest producer of tea. Therefore, tea is an important commercial crop in China, playing a significant role in domestic and foreign markets. It is necessary to make accurate and timely maps of the distribution of tea plantation areas to for plantation management and decision making. In the present study, the town of Menghai in the Xishuangbanna Dai Autonomous Prefecture, Yunnan Province, was chosen as the study area, and GF-1 remotely sensed data from 2014-2017 were chosen as the data source. Image texture, spectral and geometry features were integrated, while feature space was built by separability and thresholds algorithms (SEaTH) with decorrelation. Object-oriented image analysis (OOIA) with a classification and regression tree (CART) algorithm was utilized to map tea plantation areas. The overall accuracy and Kappa coefficient of the proposed method were 92.00% and 0.81, respectively, 4% and 0.15 better than the results of the maximum likelihood method. The tea plantation area increased by 14 km 2 from 2014 to 2017, with slight fluctuations during this period.
**Order of Authors:**	Jinliang Wang, Ph.D.
	lei liang
	fei deng
	deyang kong
**Additional Information:**	
**Question**	**Response**
**Financial Disclosure**<br><br>Enter a financial disclosure statement that describes the sources of funding for the work included in this submission. Review the submission guidelines for detailed requirements. View published research articles from *PLOS ONE* for specific examples.<br><br>This statement is required for submission and **will appear in the published article** if the submission is accepted. Please make sure it is accurate.	The author(s) received no specific funding for this work.

**Unfunded studies**<br>Enter: *The author(s) received no specific funding for this work.*<br><br>**Funded studies**<br>Enter a statement with the following details:<br>• Initials of the authors who received each award<br>• Grant numbers awarded to each author<br>• The full name of each funder<br>• URL of each funder website<br>• Did the sponsors or funders play any role in the study design, data collection and analysis, decision to publish, or preparation of the manuscript?<br>• **NO** - Include this sentence at the end of your statement: *The funders had no role in study design, data collection and analysis, decision to publish, or preparation of the manuscript.*<br>• **YES** - Specify the role(s) played.<br><br>* typeset	
**Competing Interests**<br><br>Use the instructions below to enter a competing interest statement for this submission. On behalf of all authors, disclose any competing interests that could be perceived to bias this work—acknowledging all financial support and any other relevant financial or non-financial competing interests.<br><br>This statement is required for submission and **will appear in the published article** if the submission is accepted. Please make sure it is accurate and that any funding sources listed in your Funding Information later in the submission form are also declared in your Financial Disclosure statement.<br><br>View published research articles from *PLOS ONE* for specific examples.	The authors have declared that no competing interests exist.

Powered by Editorial Manager® and ProduXion Manager® from Aries Systems Corporation

**NO authors have competing interests**<br><br>Enter: *The authors have declared that no competing interests exist.*<br><br>**Authors with competing interests**<br><br>Enter competing interest details beginning with this statement:<br><br>*I have read the journal's policy and the authors of this manuscript have the following competing interests: [insert competing interests here]*<br><br>* typeset	
**Ethics Statement**<br><br>Enter an ethics statement for this submission. This statement is required if the study involved:<br><br>• Human participants<br>• Human specimens or tissue<br>• Vertebrate animals or cephalopods<br>• Vertebrate embryos or tissues<br>• Field research<br><br>Write "N/A" if the submission does not require an ethics statement.<br><br>General guidance is provided below. Consult the submission guidelines for detailed instructions. **Make sure that all information entered here is included in the Methods section of the manuscript.**	N/A

Powered by Editorial Manager® and ProduXion Manager® from Aries Systems Corporation

**Format for specific study types**<br><br>**Human Subject Research (involving human participants and/or tissue)**<br>• Give the name of the institutional review board or ethics committee that approved the study<br>• Include the approval number and/or a statement indicating approval of this research<br>• Indicate the form of consent obtained (written/oral) or the reason that consent was not obtained (e.g. the data were analyzed anonymously)<br><br>**Animal Research (involving vertebrate animals, embryos or tissues)**<br>• Provide the name of the Institutional Animal Care and Use Committee (IACUC) or other relevant ethics board that reviewed the study protocol, and indicate whether they approved this research or granted a formal waiver of ethical approval<br>• Include an approval number if one was obtained<br>• If the study involved *non-human primates*, add *additional details* about animal welfare and steps taken to ameliorate suffering<br>• If anesthesia, euthanasia, or any kind of animal sacrifice is part of the study, include briefly which substances and/or methods were applied<br><br>**Field Research**<br><br>Include the following details if this study involves the collection of plant, animal, or other materials from a natural setting:<br>• Field permit number<br>• Name of the institution or relevant body that granted permission	
**Data Availability**<br><br>Authors are required to make all data underlying the findings described fully available, without restriction, and from the time of publication. PLOS allows rare exceptions to address legal and ethical concerns. See the PLOS Data Policy and FAQ for detailed information.	Yes - all data are fully available without restriction

A Data Availability Statement describing where the data can be found is required at submission. Your answers to this question constitute the Data Availability Statement and **will be published in the article**, if accepted.<br><br>**Important:** Stating 'data available on request from the author' is not sufficient. If your data are only available upon request, select 'No' for the first question and explain your exceptional situation in the text box.<br><br>Do the authors confirm that all data underlying the findings described in their manuscript are fully available without restriction?	
**Describe where the data may be found in full sentences. If you are copying our sample text, replace any instances of XXX with the appropriate details.**<br><br>• If the data are **held or will be held in a public repository**, include URLs, accession numbers or DOIs. If this information will only be available after acceptance, indicate this by ticking the box below. For example: *All XXX files are available from the XXX database (accession number(s) XXX, XXX.).*<br>• If the data are all contained **within the manuscript and/or Supporting Information files**, enter the following: *All relevant data are within the manuscript and its Supporting Information files.*<br>• If neither of these applies but you are able to provide **details of access elsewhere**, with or without limitations, please do so. For example:<br><br>*Data cannot be shared publicly because of [XXX]. Data are available from the XXX Institutional Data Access / Ethics Committee (contact via XXX) for researchers who meet the criteria for access to confidential data.*<br><br>*The data underlying the results presented in the study are available from (include the name of the third party*	All relevant data are within the manuscript and its Supporting Information files

and contact information or URL).<br>• This text is appropriate if the data are owned by a third party and authors do not have permission to share the data.<br><br>* typeset	
Additional data availability information:	Tick here if the URLs/accession numbers/DOIs will be available only after acceptance of the manuscript for publication so that we can ensure their inclusion before publication.

Powered by Editorial Manager® and ProduXion Manager® from Aries Systems Corporation

Manuscript

Click here to access/download;Manuscript;manuscript.docx

MAPPING PU'ER TEA PLANTATIONS FROM GF-1 IMAGES USING OOIA AND CART

LIANG, L.[1,2,3]– WANG, J. L. [1,2,3,*] –DENG, F. [1,2,3]–KONG, D.Y. [1,2,3]

[1] *Faculty of Geography, Yunnan Normal University, Kunming 650500, China*

[2] *Key Laboratory of Resources and Environmental Remote Sensing for Universities in Yunnan, Kunming 650500, China*

[3] *Remote Sensing Research Laboratory, Center for Geospatial Information Engineering and Technology of Yunnan Province, Kunming 650500, China*

** Corresponding author*

e-mail: jlwang@ynnu.edu.cn

Abstract. Tea is the most popular drink worldwide, and China is the largest producer of tea. Therefore, tea is an important commercial crop in China, playing a significant role in domestic and foreign markets. It is necessary to make accurate and timely maps of the distribution of tea plantation areas to for plantation management and decision making. In the present study, the town of Menghai in the Xishuangbanna Dai Autonomous Prefecture, Yunnan Province, was chosen as the study area, and GF-1 remotely sensed data from 2014-2017 were chosen as the data source. Image texture, spectral and geometry features were integrated, while feature space was built by separability and thresholds algorithms (SEaTH) with decorrelation. Object-oriented image analysis (OOIA) with a classification and regression tree (CART) algorithm was utilized to map tea plantation areas. The overall accu-racy and Kappa coefficient of the proposed method were 92.00% and 0.81, respectively, 4% and 0.15 better than the results of the maximum likelihood method. The tea plantation area increased by 14 km^2 from 2014 to 2017, with slight fluctuations during this period.

Keywords: *Pu'er tea, OOIA, Feature selection, Decision tree, Change detection*

Introduction

Tea is the most popular drink worldwide (Li et al., 2005), and the plantation and production of tea play an important role in agricultural economies and country development. Furthermore, the excessive plantation of tea has many negative impacts on environ-ments such as landslides and water and soil loss (Dihkan et al.,2013); therefore, the continuous moni-toring of tea-growing areas has significant importance.

Traditionally, related departments use manual methods to monitor tea distribu-tion, but these methods are inefficient, have a low precision, are expensive, and are challenged by the need for spatially continuous monitoring over large areas.

Remote sensing technology can obtain spatially continuous information on earth surfaces (Xu et al.,2016), which has plenty of applications in tea spatial distribution extraction and monitoring, because this technology has wide data coverage, fast data-update speeds, and low costs.

However, different tea growing area have spectral differences due to canopy structure and traits, plantation size, crop health and growing period, enlarging the within-class distance of tea in spectral features. The spectral reflectance of tea is close to that of other vegetation, such as fruit trees and bushes, narrowing the class distance. Therefore, the spectral mixing phenomenon makes it extremely difficult to extract tea plantation distributions (Rajapakse et al.,2002). For example, He (He et al.,2006) used an artificial neural network (ANN) to classify vegetation and found that the extraction of bushes and tea had a relatively low accuracy, but the extraction of other vegetation achieved a high accu-racy. One effective method of plantation distribution extraction is to use spatial in-formation based on texture features. For instance, Xu (Xu et al.,2016) built a decision tree

based on the texture features of anisotropic strength to extract the tea plantation distribution in mountain areas. Dihkan (Dihkan et al.,2013) uses the Gabor texture feature to train a support vector machine classifier (SVM) and extract tea plantations distribution in black sea areas, achieving an overall accuracy above 90%. Another method is using time series analysis to extract temporal feature based on regular annual growth cycle of tea. For example, Li (Li et al.,2019) used high-spatiotemporal-resolution VENμS images to extract temporal and spectral features and utilized decision tree to mapping tea plantation. Zhu (Zhu et al.,2019) inte-grate multi-temporal features to train a random forest classifier based on middle spital resolution satellite remote sensing data-Sentinel-2. However, high-temporal-resolution means either low spatial resolution such as MODIS or small coverage such as VENμS, which not able to map large area. Due to data limitations, most previous studies based on mid/low-resolution satellite remote sensing data were unable to effectively extract texture features in tea-growing areas, while few high-resolution-based studies used aerial remote sensing data, with high costs and a relatively small coverage.

High-resolution satellite remote sensing data contain sufficient targets details, advancing the effect of the inside texture and structure features of targets. However, too exhaustive details may cause salt-and-pepper noise in information extraction and image classification. OOIA solves this problem effectively. For instance, Mallinis (Mallinis et al.,2008) used OOIA to extract the distribution of vegetation based on Quickbird satellite remote sensing data, which had a spatial resolution of 0.61 m and improved the classification accuracy. Tang (Tang et al.,2020) integrated OOIA with convolutional neural network to mapping tea plantation, achieving comparable results and can be used with limited training samples.

Menghai town in the Xishuangbanna Dai Autonomous Prefecture, Yunnan Prov-ince, was chosen as the study area, and GF-1 remotely sensed data from 2014-2017 were chosen as the data source. In this study, OOIA and texture features were com-bined, while features selected by SEaTH, the CART algorithm and a maximum likeli-hood algorithm were compared to find the best way to extract the tea plantation area and analyze the plantation distribution and changes. In that case, the change trends of tea plantations can be analyzed to assist relevant departments in decision making.

Study area and data source

Study area

Menhai county is located in the southwestern part of Yunnan province, the southwest corner of China. The longitude of this county is between 99°56′E ~ 100°41′E, and the latitude is between 21°28′N ~ 22°28′N (*Fig. 1*). The study area has a tropical monsoon climate, with 1136-1513 ml precipitation per year. The annual average tem-perature is approximately 18.9℃ - 22.6℃, which is extremely suitable for tea growth, making this area one of the original plantations of “Pu Er tea”. As of 2017, the overall tea plantation area was approximately 710 thousand acres, making the tea industry the largest and most profitable industry in Menghai County. Menghai town is located in eastern Menghai County, which is the political, economy, and cultural center of Menghai County. The area of this town is 365.38$\boldsymbol{km^2}$,, of which 45.3$\boldsymbol{km^2}$, is tea plan-tation area, which makes the tea industry play a serious role in the local economy.

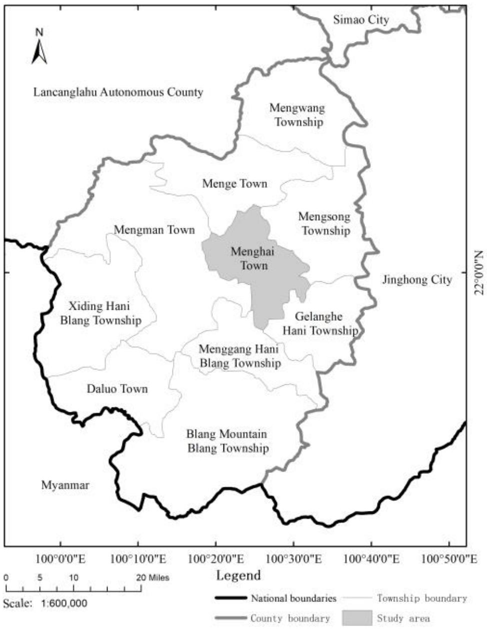

Figure 1. *Study area*

Data source and preprocessing

The PMS(Panchromatic and multispectral sensors) data products from 2014-2017, which were obtained from the GF-1 satellite data, were used in the present study (*Table 1*). The preprocesses were supported by ENVI 5.3 software and comprised of seven steps. Primarily, radiation collaboration, flash atmosphere correction, orthographical correction, image pan sharping and image mosaicking were completed. Then, 2017 data were selected as the base image to register the other three images by im-age-to-image registering (Wei et al.,2015, Sun et al.,2013, Deng et al.,2014, Deng et al.,2014). The RMS of the image registering result was con-trolled below 1. Finally, vector data of Menghai town were used to mask the image to obtain four scene images of Menghai town.

Table 1. *GF-1 SATELLITE PMS DATA PARAMETERS*

Parameters	2 m pan/8 m multiresolution	Parameters
Spectral range	Pan	0.45-0.90 µm
	Multispectral	0.45-0.52 µm
		0.52-0.59 µm
		0.63-0.69 µm
		0.77-0.89 µm
Spatial resolution	Pan	2 m
	Multispectral	8 m
Swath	60 km (2 camera)	
Temporal (sway)	4 day	
Temporal (not sway)	41day	

Methods

Research workflow

A six-step method was used to analyze the tea plantations. First, the normalized difference vegetation index (NDVI) was calculated from the preprocessed image. Second, OTSU algorithm was used to select best NDVI threshold to separate non-vegetation area from vegetation area Third step was multiresolution segmentation, but the optimal parameters first had to be obtained. The fourth step was building feature space by the selection of best features using SEaTH. The fifth step was a comparison between the performance of the maximum likelihood algorithm and the CART decision tree to determine the best method. Finally, the best-performing method was utilized to extract tea distribution information from the rest of the image and analyze the changing situation (*Fig. 2*).

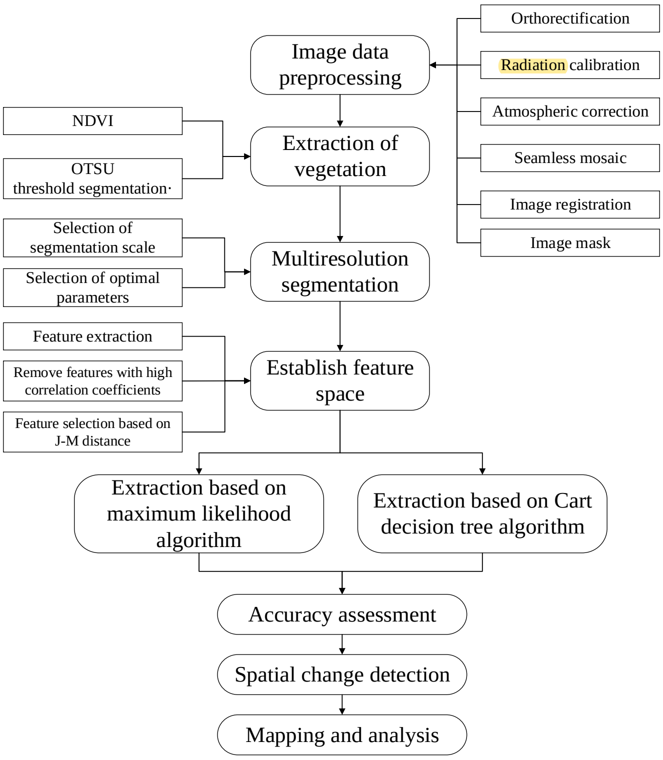

Figure 2. *Research workflow*

Extraction of vegetation

The NDVI is a well-performing index to indicate vegetation conditions on the surface. This index is obtained by the spectral reflectance of available light between 400 and 700 nm because vegetation has special reflectance and absorption characteristics in this band. The NDVI is calculated as follows:

$$NDVI = \frac{NIR-R}{NIR+R} \quad \text{(Eq.1)}$$

where NIR is the spectral reflectance of near infrared channel near 860μm, while R is the spectral reflectance of red channel near 660μm.

Because vegetation areas contain tea plantation areas, vegetation areas can be easily distinguished from non-vegetation areas by the NDVI threshold. In that case, the selection of the best-performing threshold is the most crucial stage.

The OTSU algorithm was proposed by Otsu (Otsu et al.,1979), a Japanese researcher, in 1979, for image binarization, which was widely used to select the best threshold for grayscale segmentation in digital image processing. In this algorithm, the image is splited into two classes, frontward and backward, by selecting the histogram value for

segmentation that makes the variance highest between the two classes. In other words, this algorithm calculates the lowest point between two classifications in the histogram.

NDVI was calculated from preprocessed image data from 2014 to 2017 to select the optimized threshold (*Table 2*). Figure 3 shows the histogram of NDVI in 2017. The histogram can be considered to have two peak forms, which makes the OTSU algorithm suitable. Figure 4 shows the results of the vegetation extraction for 2017, which achieved a quite accurate performance.

Table 2. *Optimal thresholds in 2014-2017*

**year**	2014	2015	2016	2017
**threshold**	0.44	0.42	0.53	0.43

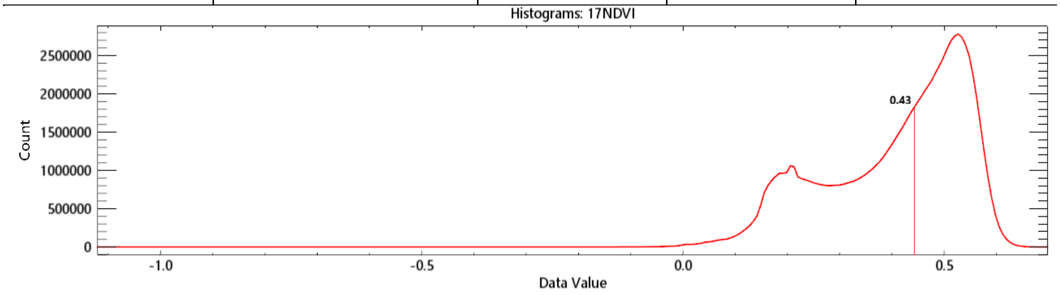

Figure 3. *NDVI histogram in 2017*

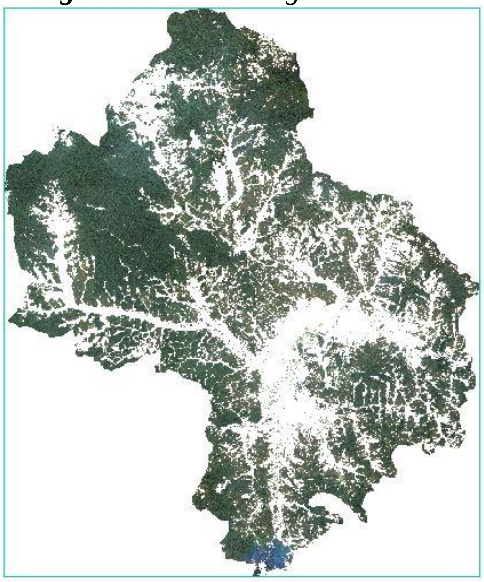

Figure 4. *2017 vegetation extraction results*

Selection of optimal parameters

To take advantage of the high-resolution feature and neighbor relationship of GF-1 satellite data and prevent noise, a multiresolution segmentation method, which combines neighbor pixels based on spectrum and shape to create image objects, was utilized(Liu et al.,2009). In this stage, the selection of segmentation scale (SS), weight of spectrum (WS1), weight of shape (WS2), weight of smoothness (WS3), and weight of compactness (WC) controlled the performance of segmentation.

The estimation of scale parameters (ESP) is an optimal parameter selection method based on local variance (LV) and the rate of change (ROC) of image homogeneity(Drǎ

gu et al.,2010). LV is the average of the neighborhood standard deviation in the entire image. Along with the increase in SS, the inner homogeneity of the image object increased, and heterogeneity with other image objects decreased, causing the LV to rise and a slower change rate. When the optimal scale was achieved, the image objects had the highest similarity with earth surface features in reality, LV peaked, and ROC increased gradually. After visual assessment, the size and shape of the results were the most similar to the tea plantation area.

In addition to the SS, the WS1 and WS2 control the segmentation results. WS2 also considers WS3 and WC. WS3 and WC are used to control the smoothness of the image object border and separation.

Based on the idea that the control variable is optimal, all SS and other parameters were traversed to integrate ESP and visual assessment to select optimal parameters. The results indicate that the optimal SS was 66, the optimal WS was 0.5, and the optimal WC was 0.7 (*Fig. 5*).

When a larger SS than the optimal SS was used (*Fig. 6*), e.g., 206, the area was too large, and details were missing, which can cause low accuracy in the classification step. When the SS was overlarge, the ROC of LV performing violent fluctuated, which is not the optimal parameter.

When a lower WC was used (*Fig. 7*), the shape of the image object was too regular, and the border was too smooth, which was not the same as reality (*Fig. 7 (b, c)*) and had a negative impact on segmentation. The optimal parameters resulted in the most complete extraction of tea plantation information, with distinct borders.

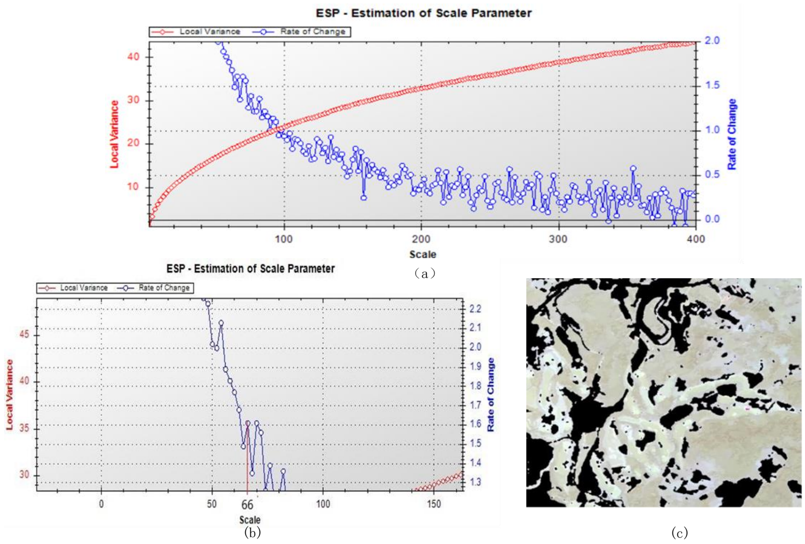

Figure 5. *Multiresolution segmentation (SS=66, WS_1=0.5, WC=0.7): (a) variance curve, (b) optimal segmentation scale and (c) image object*

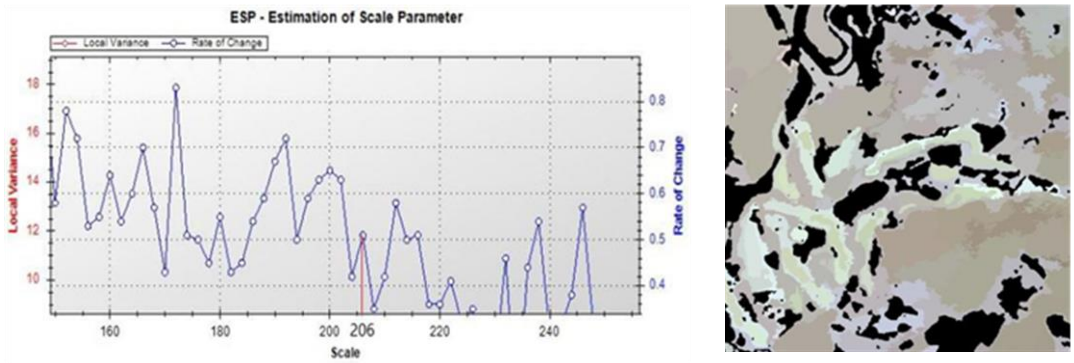

(a) (b)

__Figure 6.__ Multiresolution segmentation (SS=206, WS_1=0.5, WC=0.7): (a) variance curve and (b) image object

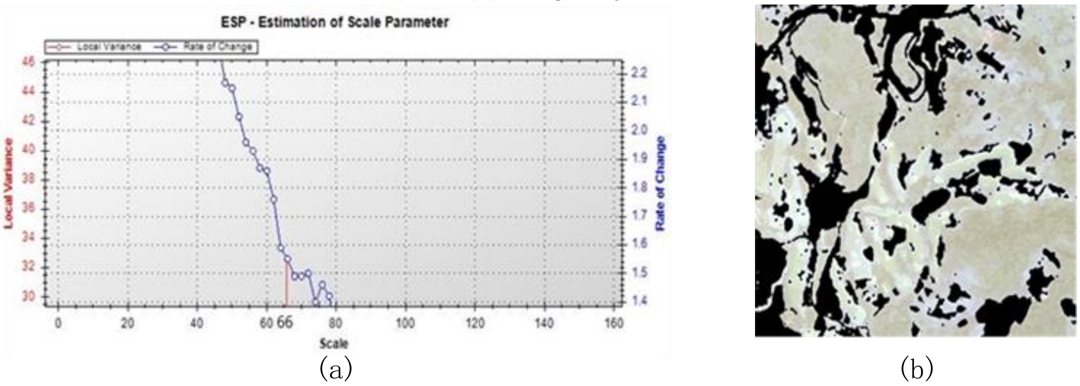

(a) (b)

__Figure 7.__ Multiresolution segmentation (SS=66, WS_1=0.5, WC=0.1): (a) variance curve and (b) image object

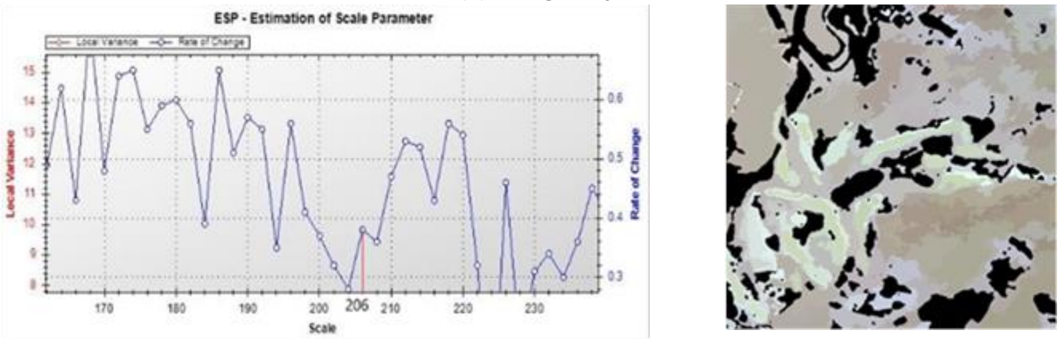

(a) (b)

__Figure 8.__ Multiresolution segmentation (SS=206, WS_1=0.5, WC=0.1): (a) variance curve and (b) image object

Feature space establishment

The eCognition software offers hundreds of features, including spectrum, shape, texture, context, and spatial relationship features. The multidimensional feature space can solve the phenomenon caused by one-dimensional spectrum extraction. However, too many features can cause dimensional disasters, which damage calculation efficiency and accuracy(Zhang et al.,2014). Therefore, high efficiency and accuracy are crucial factors for the best selection from abundant features that have high discrimination and low redundancy.

The SEaTH algorithm is a feature selection method based on J-M distance(Zhang et al.,2018), which is calculated by the statistic index of two classes, such as the mean and variance, and used to measure discrimination in one feature dimensionality. A higher J-M distance value indicates better discrimination from the two-class sample in one feature dimensionality. The J-M distance equation is:

$$J = 2(1 - e^{-B}) \quad \text{(Eq.2)}$$

$$B = \frac{1}{8(m_1 - m_2)^2} * \frac{2}{\sigma_1^2 + \sigma_2^2} + \frac{1}{2} ln \frac{\sigma_1^2 + \sigma_2^2}{2\sigma_1\sigma_2} \quad \text{(Eq.3)}$$

where m_i and σ_i^2 (i = 1 , 2) refer to the mean and variance of a two-classification sample.

The range of the J-M distance is (0-2); 0 means completely mixed, while 2 means completely separated. Because the J-M distance is unable to completely reach 2, the best result is associated with the highest J-M distance. Table 3 shows the J-M distance results calculated from different features, with results sorted by value.

***Table** 3 J-M Distances of part features*

Feature name	J-M distance
GLCM_Ang_135°	1.80
GLCM_Ang_90°	1.64
GLCM_Ang_45°	1.63
GLCM_Ang_180°	1.62
GLDV_Ang_135°	1.20
GLDV_Ang_90°	1.13
GLDV_Ang_45°	1.05
GLCM_Hom	0.74
Mean_Layer_red	0.68
GLCM_Mean	0.66

Most features with high J-M distances are different operators based on the Gray-level cooccurrence matrix (GLCM) and Gray-level difference vector (GLDV). These features have strong discrimination but high correlation, which causes high redundancy and low accuracy. Thus, features with strong correlation coefficients should be removed. Pearson's correlation coefficient was utilized to calculate the correlation coefficients, as shown in Eq.4 and 5:

$$r_{ij} = \frac{\sum_{l=1}^{K}\left(x_i^l - \overline{x_i}\right)\left(x_j^l - \overline{x_j}\right)}{\sqrt{\sum_{l=1}^{K}\left(x_i^l - \overline{x_i}\right)^2 \sum_{l=1}^{K}\left(x_j^l - \overline{x_j}\right)^2}} \quad \text{(Eq.4)}$$

$$\overline{x_i} = \frac{1}{K}\sum_{l=1}^{K} x_i^l \quad \text{(Eq.5)}$$

where x_i^l refers to the i-th feature of the l-th sample and $\overline{x_i}$ refers to the mean value of the i-th feature.

Features with high correlation coefficients and relatively low J-M distances were removed from the 20 features with the highest J-M distances. The remaining features established a feature set with four features, i.e., two texture features, one spectral feature and one geometry feature (*Table 4*).

In this feature set, the most contributory feature was GLCM Angular Second Moment (ASM), which was a function of pixel distance and angle. The texture feature is reflected by the correlation between two pixels at a certain distance and angle. The GLCM Mean and GLCM ASM equations are shown (Eq.6 and Eq.7).

$$GLCM_{MEAN} = \sum_{i,j=0}^{N-1} i(P_{i,j}) \quad \text{(Eq.6)}$$

$$GLCM_{ASM} = \sum_{i,j=0}^{N-1} P_{i,j}{}^2 \quad \text{(Eq.7)}$$

Because tea has similar spectral characteristics as other vegetation, the use of texture features can highly improve the accuracy and results of tea area extraction. Table 4 shows the best separate achievements in texture features.

Table 4 *Correlation coefficients and J-M distances of feature set*

	**GLCM 90°mean**	**Area standard deviation**	**Red band mean**	**GLCM ASM**
J-M distance	0.66	0.65	0.68	1.80
GLCM 90°mean	1	0.09	-0.44	-0.85
Area standard deviation	0.09	1	0.15	-0.08
Red band mean	-0.44	0.15	1	0.15
GLCM ASM	-0.85	-0.08	0.15	1

Classification method and accuracy assessment

The OOIA-based CART decision tree algorithm and maximum likelihood algorithm were compared using the feature set established by the above methods to extract tea from 2017 image data. The confusion matrix was calculated based on 929 non-tea samples and 255 tea samples obtained by field observation to assess accuracy(Dong et al.,2013, Jiang et al.,2015). The best algorithm was chosen by comparing the overall accuracy, Kappa coefficients, producer accuracy, and user accuracy to achieve the best performance during the extraction of tea area from multi-temporal image data.

Results

Extraction and result assessment

After the classification and accuracy assessment stage, tea plantation area (*Fig 9,10*) and accuracy (*Table 5*) were obtained. The maximum likelihood results had the lowest producer accuracy and Kappa coefficients because some other vegetation was misclassified as tea. This misclassification was successfully minimized by the CART algorithm, which improved the producer accuracy of tea to 84%. However, pine trees are widespread in the study area; because the texture of these trees is quite similar as that of tea, it is difficult to separate this vegetation from tea in remote sensing data. Thus, misclassification occurred between these two vegetation types, causing low ac-curacy, but the method could effectively separate tea from other vegetation, especially wood.

Table 5. *Accuracy assessment of 2017 classification result*

**classifier**	**Maximum likelihood**	**CART decision tree**
Overall accuracy	88%	92%
Kappa coefficient	0.66	0.73
Producer accuracy (tea)	63%	79%
Producer accuracy (other vegetation)	97%	97%
User accuracy (tea)	89%	89%

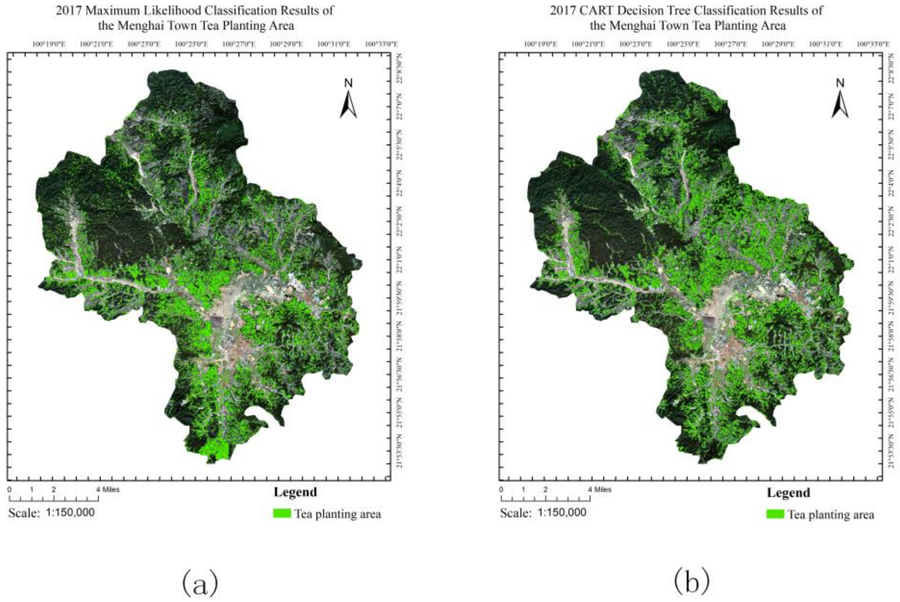

Figure 9. *Maximum likelihood and CART decision tree*

Then, this method was utilized to extract tea from three periods in 2014, 2015, and 2016, and the results contained tea-growing areas (*Fig.* 10). To examine the accuracy of the results, the traditional accuracy assessment method was used to generate a confusion matrix (*Table 6*). According to the analysis of the confusion matrix, the accuracies of 2014 and 2015 were lower than the accuracy of 2016 because of the difficulty of sample selection. Field data are difficult to obtain, while tea area is difficult to identify by visual interpretation in GF-1 image data. Some extracted tea areas were located in the deep forest, but most tea was normally planted at the edge of a forest and in the neighborhood of the residential area during field research. However, the result accuracy was satisfactory for applications.

Table 6. *Accuracy assessment of 2017 classification result*

**time**	**2014**	**2015**	**2016**
Overall accuracy	84%	86%	92%
Kappa coefficient	0.62	0.59	0.77
Producer accuracy (tea)	64%	87%	83%
Producer accuracy (other vegetation)	84%	86%	94%
User accuracy (tea)	94%	55%	80%

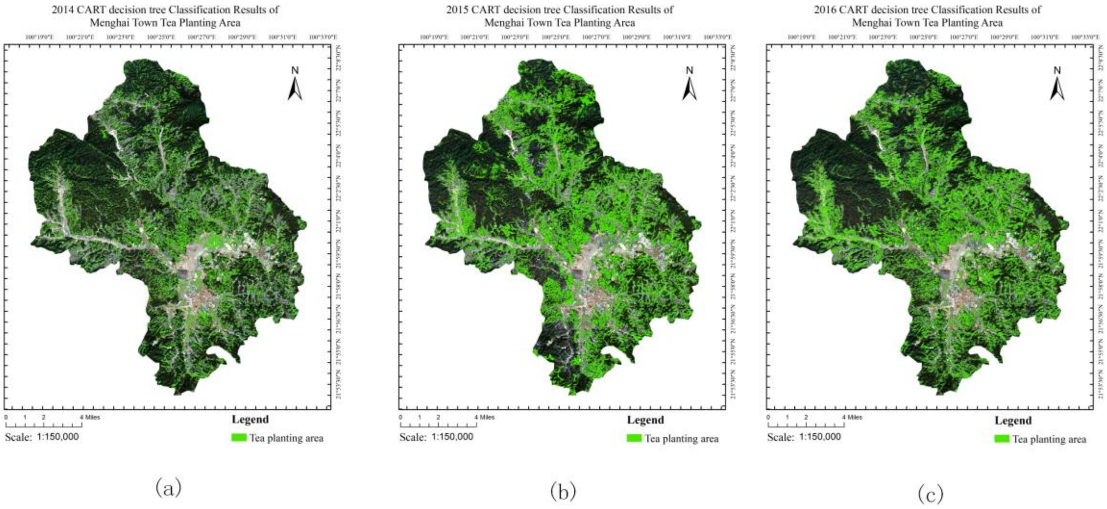

Figure 10. *Extraction results in (a)2014 (b)2015 and (c)2016*

Change detection

The eCogntion software was utilized for change detection, while the area was calculated by ARCGIS software for statistical analysis. The spatial variation distribution (*Fig. 11*) and area change situation were analyzed (*Table 7, Fig. 12, 13*). The overall pattern of spatial variation is expansion to the mid-west. The tea plantation area sharply increased between 2014 and 2015 and slowly increased between 2015 and 2016 but slightly decreased from 2016 to 2017.

Table 7. *Tea plantation changes during 2014-2017*

**time**	2014	2015	2016	2017
**area/acre**	14953.65	18130.59	18006.4	18422.41

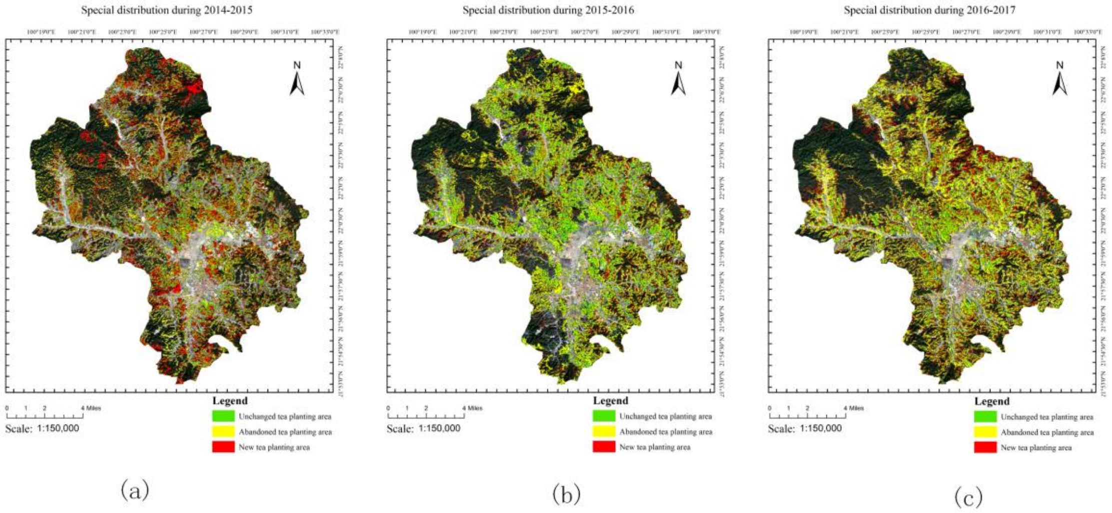

Figure 11. *Spatial distribution during (a)2014-2015 (b)2015-2016 and (c)2016-2017*

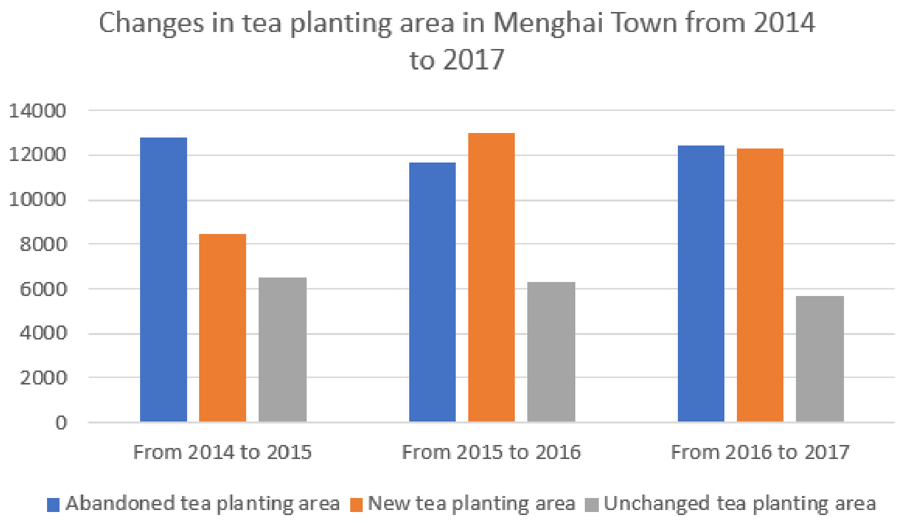

Figure 12. *Area change from 2014 to 2017*

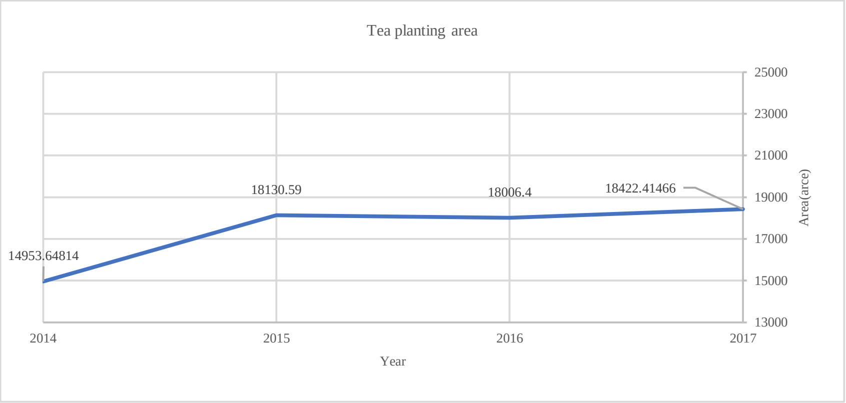

Figure 13. *Special variation from 2014 to 2017*

Discussion

In this study, our primary objective was to analyze and propose a tea plantation mapping method and monitor the tea plantation area in Menghai Town during 2014 to 2017. The NDVI thershold segmentation metohd was used to extract vegtation area from GF-1 data. The optimal segmentation parameters, such as segmentation scale (SS), weight of spectrum (WS1), weight of shape (WS2), weight of smoothness (WS3), and weight of compactness (WC), were selected by calculate and anaylze the local variance (LV) and the rate of change (ROC) of image homogeneity. The experiment results indicated that the optimal extraction of tea plantation are achieved when SS is set to 66, the WS is set to 0.5, and the WC is set to 0.7. If SS set higher, the image object is too large and details are missing. This finding indicated that for tea plantation mapping SS can not set to high because of the planting characteristics and vegetation form.

In order to build feature space for image objects to utlize the classification algorithm, SEaTH method is used to calculate the J-M distance of both spectrul and texture features between tea plantation and other vegtations. The experiment results showed that texture features, such as GLCM and GLDV have the highest J-M distance, and most of them are higher than 1.5. The highest one is GLCM in angle 135°, which is 1.80. Besides, the mean value of red band also have a relatively high J-M distance. Then decorelation method was

used to remove the corelated features by calculate the correlation coefficients in order to build feature space without redundancy. The results indicated that the mean of GLCM in angle 90°, area standard deviation, mean of red band and GLCM ASM have low corelation coefficients and relatively high J-M distance, which can be used to build features to utilize the classification methods.

The CRAT algorithm and maximum likelihood algorithm was compared at data in 2017 to find the most suitable algorithm, using the builded feature space. The experiments results showed that CRAT algorithm achieve 92% overall accuracy and 0.81 Kappa coefficient outperformed maximum likelihood algorithm 4% in overall accuracy and 0.06 in Kappa coefficient. These findings clearly showed that CART algorithm is more sutiable for the tea plantation mapping, and the combination of special feature, spectral feature and texture feature can achieve promising results in tea plantation mapping.

Then, the best performing algorithm was used to mapping tea plantation at 2014 to 2017. Accuracy assement was done by confusion matrix and kappa coefficient(*Table 6*). All overall accuracy were above 80%, which prove the effectiveness of proposed method.

Conclusion

Menghai Town of Xishuangbanna Dai Autonomous Prefecture, Yunnan Province, was chosen as the study area, and GF-1 remotely sensed data from 2014-2017 were chosen as the data source. Image texture, spectral and geometry features were integrated, while feature space was built by separability and thresholds algorithms (SEaTH) with decorrelation. Object-oriented image analysis (OOIA) with classification and a regression tree (CART) algorithm were utilized to extract tea plantation areas. The salient findings of the study are as follows:

- OOIA can successfully suppressed noise by object-based classification and improve the effect of extraction.
- The selection of segmentation scale and parameters is a critical factor in OOIA. A scale that is too small causes over segmentation, while a scale that is too large scale mixes different earth surface features in one image object.
- The feature set built by the SEaTH algorithm and decorrelation with texture features can effectively avoid spectral confusion.
- OOIA combined with the CART decision tree algorithm and texture features solves the misclassification and missing-classification issues, yielding a Kappa coefficient up to 0.81 and an overall accuracy of 82%, higher than the maximum likelihood method.
- According to the extraction results, tea plantation areas are mainly distributed in the neighborhoods of residential areas with moderate slopes because tea must be cultivated in easily drained areas and be convenient for farmers to reach. Based on variation analysis, we found that tea plantations expanded towards areas with sparse forests and moderate slopes.

Expectations

The extraction of tea plantations based on RS still needs to be discovered. Currently, methods still have flaws in accuracy and efficiency. In consideration of this, we will focus on the following aspect in feature research:

- ESP is utilized to select the segmentation scale, but other parameters still need to be selected based on a visual assessment. The parameter selection method can be

improved by automatically selecting the weight of the spectrum and the weight of compactness based on LV to achieve the optimal segmentation result.

- According to the texture and spectral confusion, different data and extraction methods can be used: consider the growing period of tea based on low revisit period RS data; multisource data fusion such as hyperspectral and high-resolution RS data to analyze the spectral and texture features; integrate novel classification algorithms such as artificial intelligence and machine learning to improve accuracy.

Acknowledgements. ------ This work was supported by Multi-government International Science and Technology Innovation Cooperation Key Project of National Key Research and Development Program of China , grant number 2018YFE0184300; the National Natural Science Foundation of P.R. China, grant number 41561048; the Program for Innovative Research Team (in Science and Technology) in the University of Yunnan Province, IRTSTYN; and the Undergraduate Research Innovation Foundation of Yunnan Normal University. The authors are grateful to the editor and anonymous referees for their valuable comments and helpful suggestions. We wish to thank professor Cheng Wang and Xiaohuang Xi for their advice.

REFERENCES

[1] Deng Shubin, Chen Qiujin, Du Huijian. ENVI Remote Sensing Image Processing Method, 2nd ed.; Beijing Higher Education Press: Beijing, China, 2014, 132-228,

[2] Deng Shubin, Chen Qiujin, Du Huijian. ENVI Remote Sensing Image Processing Method, 2nd ed.; Beijing Higher Education Press: Beijing, China, 2014, 132-228.

[3] Dihkan M, Guneroglu N, Karsli F. Remote sensing of tea plantations using an SVM classifier and pattern-based accuracy assessment technique. International Journal of Remote Sensing 2013, 34(23), 8549-8565.

[4] Dong Lianying, Xing Lixin, Pan Jun. Vegetation Classification in Hyper-spectral Image with CART Decision Tree, Journal of Jilin University, Information Science Edition 2013 ,1, 83-89.

[5] Drăgu, Lucian, Tiede D, Levick S R. ESP: a tool to estimate scale parameter for multiresolution image segmentation of remotely sensed data, International Journal of Geographical Information Science 2010, 24(6),859-871.

[6] Gonzalez R C, Woods R E, Eddins S L. Digital Image Processing Using MATLAB, 2nd ed.; Publishing House of Electronics Industry: Beijing, China, 2009, 135-136.

[7] He L, Kong F, Shen Z. Artificial Neural Network Ensemble for Land Cover Classification, World Congress on Intelligent Control & Automation 2006.

[8] Jiang Haowei, Ren Hongquan, Qin Xianfeng. Auto Interpretation and Accuracy Evaluation of Images Based on eCognition, Bulletin of Surveying and Mapping 2015 ,10, 81-84.

[9] Jun Zhu, Ziwu Pan, Hang Wang, Peijie Huang, Jiulin Sun, Fen Qin, Zhenzhen Liu. An Improved Multi-temporal and Multi-feature Tea Plantation Identification Method Using Sentinel-2 Imagery, Sensors 2019, 19(9), 2087.

[10] Li Zhuo, He Lingqi, Tea and Meteorology, 1st ed.; Meteorological Press: Beijing, China, 2005; 25-26.

[11] Li, Zhang, Li, Zhang. Mapping the Spatial Distribution of Tea Plantations Using High-Spatiotemporal-Resolution Imagery in Northern Zhejiang 2019, Forests 10:10, 856.

[12] Liu Jianhua. MAO Zheng-yuan. Review of High Spatial Resolution Remote Sensing Image Segmentation Methods, Remote Sensing Information 2009, 95-101.

[13] Liu Shuhan, Gu Xingfa, Yu Tao. Object-Oriented Classification method based on GF-1 Multi-spectral Remote Sensing image, Surveying Science 2014,12, 91-103.

[14] Mallinis G, Koutsias N, Tsakiri-Strati M, et al. Object-based classification using Quickbird imagery for delineating forest vegetation polygons in a Mediterranean test site, ISPRS Journal of Photogrammetry & Remote Sensing 2008, 63(2), 237-250.

[15] Otsu N. A Threshold Selection Method from Gray-Level Histograms, IEEE Transactions on Systems, Man, and Cybernetics 1979, 9(1), 62-66.

[16] Rajapakse R M S S, Tripathi N K, Honda K. Spectral characterization and LAI modelling for the tea (Camellia sinensis (L.) O. Kuntze) canopy, International Journal of Remote Sensing 2002, 23(18), 3569-3577

[17] Sun Jiabing. Principle and Application of Remote Sensing, 3rd ed.; Wuhan University Press: Wuhan, China, 2013, 108-224.

[18] Sven Nussbaum, Gunter Menz. Object-Based Image Analysis and Treaty Verification, Springer Netherlands 2008,51-62.

[19] Wei Yuchun. Remote Sensing Digital Image Processing Tutorial, 2nd ed.; Science Press: Beijing, China, 2015, 185-304.

[20] Xu Weiyan, Sun Rui, Jin Zhifeng. Extracting tea plantations based on ZY-3 satellite data, Transactions of the Chinese Society of Agricultural Engineering (Transactions of the CSAE) 2016, 32(Supp.1), 161-168.

[21] Zhang Jing. Classification and Feature Selection on High-dimensional and Small-sampling Data 2014, Hefei University of Technology.

[22] Zixia Tang, Mengmeng Li, Xiaoqin Wang. Mapping Tea Plantations from VHR Images Using OBIA and Convolutional Neural Networks. Remote Sensing 2020, 12:18, pages 2935.
