# Supplementary material for: Mapping Pu’er tea plantations from GF-1 images using Object-Oriented Image Analysis (OOIA) and Support Vector Machine (SVM)
Source: PLoS One. 2023 Feb 7;18(2):e0263969. doi: 10.1371/journal.pone.0263969 (PMC9904455; doi:10.1371/journal.pone.0263969)
Supplement: Attachment — Submitted filename: PONE-D-21-11402_R2_reviewed2.pdf [file pone.0263969.s006.pdf]

PLOS ONE

Mapping Pu'er Tea Plantations from GF-1 Images Using Object-Oriented Image Analysis (OOIA) and Support Vector Machine (SVM)

--Manuscript Draft--

**Manuscript Number:**	PONE-D-21-11402R2
**Article Type:**	Research Article
**Full Title:**	Mapping Pu'er Tea Plantations from GF-1 Images Using Object-Oriented Image Analysis (OOIA) and Support Vector Machine (SVM)
**Short Title:**	Mapping Pu'er tea plantations from GF-1 images using OOIA and SVM
**Corresponding Author:**	Jinliang Wang, Ph.D.<br>Yunnan Normal University<br>Kunming, Yunnan CHINA
**Keywords:**	Pu'er tea, OOIA, Feature selection, SVM, Change detection
**Abstract:**	Tea is the most popular drink worldwide, and China is the largest producer of tea. Therefore, tea is an important commercial crop in China, playing a significant role in domestic and foreign markets. It is necessary to make accurate and timely maps of the distribution of tea plantation areas for plantation management and decision making. In the present study, the town of Menghai in the Xishuangbanna Dai Autonomous Prefecture, Yunnan Province, China, was chosen as the study area, and GF-1 remotely sensed data from 2014-2017 were chosen as the data source. Image texture, spectral and geometrical features were integrated, while feature space was built by SEparability and THresholds algorithms (SEaTH) with decorrelation. Object-Oriented Image Analysis (OOIA) with a Support Vector Machine (SVM) algorithm was utilized to map tea plantation areas. The overall accuracy and Kappa coefficient of the proposed method were 93.14% and 0.81, respectively, 3.61% and 0.05, 6.99% and 0.14, 6.44% and 0.16 better than the results of CART method, Maximum likelihood method and CNN based method. The tea plantation area increased by 4,095.36 acre from 2014 to 2017, while the fastest-growing period is 2015 to 2016.
**Order of Authors:**	lei liang
	Jinliang Wang, Ph.D.
	fei deng
	deyang kong
**Response to Reviewers:**	Dear Reviewer:<br>Thank you for your comments concerning our manuscript entitled “Mapping Pu'er tea plantations from GF-1 images using OOIA and SVM” (ID: PONE-D-21-11402R1). Those comments are valuable and helpful for revising and improving our paper, as well as the important guiding significance to our researches. We have studied comments carefully and have made correction which we hope meet with approval. The responses to the reviewer’s comments can be found in attachment files.
**Additional Information:**	
**Question**	**Response**
**Financial Disclosure**<br><br>Enter a financial disclosure statement that describes the sources of funding for the work included in this submission. Review the submission guidelines for detailed requirements. View published research articles from *PLOS ONE* for specific examples.	The author(s) received no specific funding for this work.

Powered by Editorial Manager® and ProduXion Manager® from Aries Systems Corporation

This statement is required for submission and **will appear in the published article** if the submission is accepted. Please make sure it is accurate.<br><br>**Unfunded studies**<br>Enter: *The author(s) received no specific funding for this work.*<br><br>**Funded studies**<br>Enter a statement with the following details:<br>• Initials of the authors who received each award<br>• Grant numbers awarded to each author<br>• The full name of each funder<br>• URL of each funder website<br>• Did the sponsors or funders play any role in the study design, data collection and analysis, decision to publish, or preparation of the manuscript?<br>• **NO** - Include this sentence at the end of your statement: *The funders had no role in study design, data collection and analysis, decision to publish, or preparation of the manuscript.*<br>• **YES** - Specify the role(s) played.<br><br>* typeset	
**Competing Interests**<br><br>Use the instructions below to enter a competing interest statement for this submission. On behalf of all authors, disclose any competing interests that could be perceived to bias this work—acknowledging all financial support and any other relevant financial or non-financial competing interests.<br><br>This statement is required for submission and **will appear in the published article** if the submission is accepted. Please make sure it is accurate and that any funding sources listed in your Funding Information later in the submission form are also declared in your Financial Disclosure statement.<br><br>View published research articles from *PLOS ONE* for specific examples.	The authors have declared that no competing interests exist.

**NO authors have competing interests**<br><br>Enter: *The authors have declared that no competing interests exist.*<br><br>**Authors with competing interests**<br><br>Enter competing interest details beginning with this statement:<br><br>*I have read the journal's policy and the authors of this manuscript have the following competing interests: [insert competing interests here]*<br><br>* typeset	
**Ethics Statement**<br><br>Enter an ethics statement for this submission. This statement is required if the study involved:<br><br>• Human participants<br>• Human specimens or tissue<br>• Vertebrate animals or cephalopods<br>• Vertebrate embryos or tissues<br>• Field research<br><br>Write "N/A" if the submission does not require an ethics statement.<br><br>General guidance is provided below. Consult the submission guidelines for detailed instructions. **Make sure that all information entered here is included in the Methods section of the manuscript.**	N/A

**Format for specific study types**<br><br>**Human Subject Research (involving human participants and/or tissue)**<br>• Give the name of the institutional review board or ethics committee that approved the study<br>• Include the approval number and/or a statement indicating approval of this research<br>• Indicate the form of consent obtained (written/oral) or the reason that consent was not obtained (e.g. the data were analyzed anonymously)<br><br>**Animal Research (involving vertebrate animals, embryos or tissues)**<br>• Provide the name of the Institutional Animal Care and Use Committee (IACUC) or other relevant ethics board that reviewed the study protocol, and indicate whether they approved this research or granted a formal waiver of ethical approval<br>• Include an approval number if one was obtained<br>• If the study involved *non-human primates*, add *additional details* about animal welfare and steps taken to ameliorate suffering<br>• If anesthesia, euthanasia, or any kind of animal sacrifice is part of the study, include briefly which substances and/or methods were applied<br><br>**Field Research**<br><br>Include the following details if this study involves the collection of plant, animal, or other materials from a natural setting:<br>• Field permit number<br>• Name of the institution or relevant body that granted permission	
**Data Availability**<br><br>Authors are required to make all data underlying the findings described fully available, without restriction, and from the time of publication. PLOS allows rare exceptions to address legal and ethical concerns. See the PLOS Data Policy and FAQ for detailed information.	Yes - all data are fully available without restriction

A Data Availability Statement describing where the data can be found is required at submission. Your answers to this question constitute the Data Availability Statement and **will be published in the article**, if accepted.<br><br>**Important:** Stating 'data available on request from the author' is not sufficient. If your data are only available upon request, select 'No' for the first question and explain your exceptional situation in the text box.<br><br>Do the authors confirm that all data underlying the findings described in their manuscript are fully available without restriction?	
**Describe where the data may be found in full sentences. If you are copying our sample text, replace any instances of XXX with the appropriate details.**<br><br>• If the data are **held or will be held in a public repository**, include URLs, accession numbers or DOIs. If this information will only be available after acceptance, indicate this by ticking the box below. For example: *All XXX files are available from the XXX database (accession number(s) XXX, XXX.*).<br>• If the data are all contained **within the manuscript and/or Supporting Information files**, enter the following: *All relevant data are within the manuscript and its Supporting Information files.*<br>• If neither of these applies but you are able to provide **details of access elsewhere**, with or without limitations, please do so. For example:<br><br>*Data cannot be shared publicly because of [XXX]. Data are available from the XXX Institutional Data Access / Ethics Committee (contact via XXX) for researchers who meet the criteria for access to confidential data.*<br><br>*The data underlying the results presented in the study are available from (include the name of the third party*	All relevant data are within the manuscript and its Supporting Information files

and contact information or URL).<br>• This text is appropriate if the data are owned by a third party and authors do not have permission to share the data.<br><br>* typeset	
Additional data availability information:	

Powered by Editorial Manager® and ProduXion Manager® from Aries Systems Corporation

Manuscript Click here to access/download;Manuscript;Manuscript.docx

Mapping Pu'er tea plantations from GF-1 images using Object-Oriented Image Analysis (OOIA) and Support Vector Machine (SVM)

LIANG, L.[1,2,3]– WANG, J. L. [1,2,3,*] –DENG, F. [1,2,3]–KONG, D.Y. [1,2,3]

1Faculty of Geography, Yunnan Normal University, Kunming 650500, China
2Key Laboratory of Resources and Environmental Remote Sensing for Universities in Yunnan, Kunming 650500, China
3Remote Sensing Research Laboratory, Center for Geospatial Information Engineering and Technology of Yunnan Province, Kunming 650500, China
* Corresponding author
E-mail: jlwang@ynnu.edu.cn

Mapping Pu'er tea plantations from GF-1 images using Object-Oriented Image Analysis (OOIA) and Support Vector Machine (SVM)

LIANG, L.[1,2,3]– WANG, J. L. [1,2,3,*] –DENG, F. [1,2,3]–KONG, D.Y. [1,2,3]

[1] *Faculty of Geography, Yunnan Normal University, Kunming 650500, China*

[2] *Key Laboratory of Resources and Environmental Remote Sensing for Universities in Yunnan, Kunming 650500, China*

[3] *Remote Sensing Research Laboratory, Center for Geospatial Information Engineering and Technology of Yunnan Province, Kunming 650500, China*

** Corresponding author*
e-mail: jlwang@ynnu.edu.cn

Abstract

Tea is the most popular drink worldwide, and China is the largest producer of tea. Therefore, tea is an important commercial crop in China, playing a significant role in domestic and foreign markets. It is necessary to make accurate and timely maps of the distribution of tea plantation areas for plantation management and decision making. In the present study, the town of Menghai in the Xishuangbanna Dai Autonomous Prefecture, Yunnan Province, China, was chosen as the study area, and GF-1 remotely sensed data from 2014-2017 were chosen as the data source. Image texture, spectral and geometrical features were integrated, while feature space was built by SEparability and THresholds algorithms (SEaTH) with decorrelation. Object-Oriented Image Analysis (OOIA) with a Support Vector Machine (SVM) algorithm was utilized to map tea plantation areas. The overall accuracy and Kappa coefficient of the proposed method were 93.14% and 0.81, respectively, 3.61% and 0.05, 6.99% and 0.14, 6.44% and 0.16 better than the results of CART method, Maximum likelihood method and CNN based method. The tea plantation area increased by 4,095.36 acre from 2014 to 2017, while the fastest-growing period is 2015 to 2016.

Keywords: *Pu'er tea, OOIA, Feature selection, SVM, Change detection*

Introduction

Tea is the most popular drink worldwide[1], and the plantation and production of tea play an important role in agricultural economies and country development. Furthermore, the excessive plantation of tea has many negative impacts on environments such as landslides and water and soil loss[2]; therefore, the continuous monitoring of tea-growing areas has significant importance.

Traditionally, related departments use visual interpretation methods to monitor tea distribution from remote sensing image, but these methods are inefficient, have a low precision, are expensive, and are challenged by the need for spatially continuous monitoring over large areas.

Remote sensing technology can obtain spatially continuous information on earth surfaces[3], which has plenty of applications in tea spatial distribution extraction and monitoring, because this technology has wide data coverage, fast data-update speeds, and low costs.

However, different tea growing areas have spectral differences due to canopy structure and traits, plantation size, crop health and growing period, enlarging the within-class distance of tea in spectral features. The spectral reflectance of tea is close to that of other vegetation, such as fruit trees and bushes, narrowing the class distance. Therefore, the spectral mixing phenomenon makes it extremely difficult to extract tea plantation distributions [4]. For example, He[5] used an artificial neural network (ANN) to classify vegetation using Landsat Thematic Mapper (TM) images found that the extraction of bushes and tea had a relatively low accuracy, but the extraction of other vegetation achieved a high accuracy. One effective method of plantation distribution extraction is to use spatial information based on texture features. For instance, Xu[3] built a decision tree based on the texture features of anisotropic strength to extract the tea plantation distribution in mountain areas, using ZY-3 satellite data. Dihkan[2] extracted the Gabor texture feature from multispectral airborne digital image to train a support vector machine classifier (SVM) and extract tea plantations distribution in black sea areas, achieving an overall accuracy above 90%. Another method is using time series analysis to extract temporal feature based on regular annual growth cycle of tea. For example, Li[6] used high-spatiotemporal-resolution VENμS images to extract temporal and spectral features, and utilized decision tree to mapping tea plantation. Zhu[7] integrates multi-temporal features to train a random forest classifier based on middle spital resolution satellite remote sensing data Sentinel-2. However, high-temporal-resolution means either low spatial resolution such as MODIS or small coverage such as VENμS, which not able to map extensive area. Because of data limitations, most previous studies based on mid/low-resolution satellite remote sensing data were unable to effectively extract texture features in tea-growing areas, while few high spatial resolution-based studies used aerial remote sensing data, with high costs and a relatively small coverage.

High spatial resolution satellite remote sensing data contain sufficient targets details, advancing the effect of the inside texture and structure features of targets. However, too exhaustive details may cause salt-and-pepper noise in information extraction and image classification. OOIA solves this problem effectively. For instance, Mallinis[8] used OOIA to extract the distribution of vegetation based on Quickbird satellite remote sensing data, which had a spatial resolution of 0.61 m and improved the classification accuracy. Tang[9] integrated OOIA with convolutional neural network to mapping tea plantation, achieving comparable results and can be used with limited training samples.

Menghai town in the Xishuangbanna Dai Autonomous Prefecture, Yunnan Province, was chosen as the study area, and GF-1 remotely sensed data from 2014 to 2017 were chosen as the data source. In this study, OOIA and texture features were combined, while features selected by SEaTH, the CART algorithm and SVM algorithm were compared to find the best way to extract the tea plantation area and analyze the plantation distribution and changes. In that case, the change trends of tea plantations can be analyzed to assist relevant departments in decision making.

Study area and data source

Study area

Menhai county is in the southwestern part of Yunnan province, the southwest corner of China. The longitude of this county is between 99°56′E ~ 100°41′E, and the latitude is

between 21°28′N ~ 22°28′N (Fig. 1). The study area has a tropical monsoon climate, with 1136-1513 ml precipitation per year. The annual average temperature is approximately 18.9℃ - 22.6℃, which is extremely suitable for tea growth, making this area one of the original plantations of "Pu Er tea". As of 2017, the overall tea plantation area was approximately 710 thousand acres, making the tea industry the largest and most profitable industry in Menghai County. Menghai town is in eastern Menghai County, which is the political, economy, and cultural center of Menghai County. The area of this town is 365.38$\boldsymbol{km^2}$, of which 45.3$\boldsymbol{km^2}$, is tea plantation area, which shows the tea industry plays a serious role in the local economy.

Fig 1. Study area

Data source and preprocessing

The PMS(Panchromatic and multispectral sensors) data products from 2014 to 2017, which were obtained from the GF-1 satellite data, were used in the present study (Table 1). The preprocesses were supported by ENVI 5.3 software and consisting of seven steps. Primarily, radiometric calibration, FLAASH atmosphere correction, orthorectification, image pan sharping and image mosaicking were completed. Then, 2017 data were selected as the base image to register the other three images by image-to-image registering [10-13]. The RMS of the image registering result was controlled below 1 pixel. Finally, vector data of Menghai town were used to mask the image to obtain four scene images of Menghai town.

Table 1. GF-1 satellite PMS data parameters

**Parameters**	**2 m pan/8 m multiresolution**	
**Spectral range**	Pan	0.45-0.90 μm
	Multispectral	0.45-0.52 μm
		0.52-0.59 μm
		0.63-0.69 μm
		0.77-0.89 μm
**Spatial resolution**	Pan	2 m
	Multispectral	8 m
**Swath**	60 km (2 camera)	
**Temporal (sway)**	4 day	
**Temporal (not sway)**	41day	

Methods

Research workflow

A six-step method was used to analyze the tea plantations. First, the normalized difference vegetation index (NDVI) was calculated from the preprocessed image. Second, OTSU algorithm was used to select best NDVI threshold to separate non-vegetation area from vegetation area. The third step were selection of optimal segmentation and multiresolution segmentation. The fourth step was building feature space by the selection of best features using SEaTH. The fifth step was a comparison between the performing

of SVM method, CART method, Maximum likelihood method and CNN based method to determine the best method. Finally, the best-performing method was utilized to extract tea distribution information from the rest of the image and analyze the changing situation (*Fig. 2*).

Fig 2. Research workflow

Extraction of vegetation

The NDVI is a well-performing index to indicate vegetation conditions on the surface. This index is obtained by the spectral reflectance of available light and near infrared between 400 and 860 nm because vegetation has special reflectance and absorption characteristics in these bands. The NDVI is calculated as follows:

$$NDVI = \frac{NIR-R}{NIR+R} \quad (1)$$

where NIR is the spectral reflectance of near infrared channel near 860μm, while R is the spectral reflectance of red channel near 660μm.

Because vegetation areas contain tea plantation areas, vegetation areas can be easily distinguished from non-vegetation areas by the NDVI threshold. In that case, the selection of the best-performing threshold is the most crucial stage.

The OTSU algorithm was proposed by Otsu[14], a Japanese researcher, in 1979, for image binarization, which was widely used to select the best threshold for grayscale segmentation in digital image processing. In this algorithm, the image is split into two classes, frontward and backward, by selecting the histogram value for segmentation that makes the variance highest between the two classes. In other words, this algorithm calculates the lowest point between two classifications in the histogram.

NDVI was calculated from preprocessed image data from 2014 to 2017 to select the optimized threshold (*Table 2*). Figure 3 shows the histogram of NDVI in 2017. The histogram can be considered having two peak forms, which makes the OTSU algorithm suitable. Figure 4 shows the results of the vegetation extraction for 2017, which achieved a quite accurate performance.

Table 2. Optimal thresholds in 2014-2017

**year**	**2014**	**2015**	**2016**	**2017**
**threshold**	0.44	0.42	0.53	0.43

Figure 3. NDVI histogram in 2017

Figure 4. 2017 Vegetation extraction results. The base map of the LandSat8 OLI images with the following composition:R (4),G (3) and B (2).The LandSat8 OLI images were downloaded from USGS National Map Viewer.

Selection of optimal parameters

To take advantage of the high-resolution feature and neighbor relationship of GF-1 satellite data and prevent noise, a multiresolution segmentation method, which combines neighbor pixels based on spectrum and shape to create image objects, was utilized[15]. In this stage, the selection of segmentation scale (SS), weight of spectrum (WS1), weight

of shape (WS2), weight of smoothness (WS3), and weight of compactness (WC) controlled the performance of segmentation.

The estimation of scale parameters (ESP) is an optimal parameter selection method based on local variance (LV) and the rate of change (ROC) of image homogeneity[16]. LV is the average of the neighborhood standard deviation in the entire image. Along with the increase in SS, the inner homogeneity of the image object increased, and heterogeneity with other image objects decreased, causing the LV to rise and a slower change rate. When the optimal scale was achieved, the image objects had the highest similarity with earth surface features in reality, LV peaked, and ROC increased gradually. After visual assessment, the size and shape of the results were the most similar to the tea plantation area.

In addition to the SS, the WS1 and WS2 control the segmentation results. WS2 also considers WS3 and WC. WS3 and WC are used to control the smoothness of the image object border and separation.

Based on the idea that the control variable is optimal, all SS and other parameters were traversed to integrate ESP and visual assessment to select optimal parameters. The results indicate that the optimal SS was 66, the optimal WS was 0.5, and the optimal WC was 0.7 (*Fig. 5*).

When a larger SS than the optimal SS was used (*Fig. 6*), e.g., 206, the area was too large, and details were missing, which can cause low accuracy in the classification step. When the SS was overlarge, the ROC of LV performing violent fluctuated, which is not the optimal parameter.

When a lower WC was used (*Fig. 7*), the shape of the image object was too regular, and the border was too smooth, which was not the same as reality (*Fig. 7 (b, c)*) and had a negative impact on segmentation. The optimal parameters resulted in the most complete extraction of tea plantation information, with distinct borders.

Figure 5. Multiresolution segmentation (SS=66, WS_1=0.5, WC=0.7): (a) variance curve, (b) optimal segmentation scale and (c) image object

Figure 6. Multiresolution segmentation (SS=206, WS_1=0.5, WC=0.7): (a) variance curve and (b) image object

Figure 7. Multiresolution segmentation (SS=66, WS_1=0.5, WC=0.1): (a) variance curve and (b) image object

Figure 8. Multiresolution segmentation (SS=206, WS_1=0.5, WC=0.1): (a) variance curve and (b) image object

Feature space establishment

The eCognition software offers hundreds of features, including spectrum, shape, texture, context, and spatial relationship features. The multidimensional feature space can solve the phenomenon caused by one-dimensional spectrum extraction. However, too many features can cause dimensional disasters, which damage calculation efficiency and

accuracy[17]. Therefore, high efficiency and accuracy are crucial factors for the best selection from abundant features that have high discrimination and low redundancy.

The SEaTH algorithm is a feature selection method based on Jeffries Matusita(J-M) distance[17], which is calculated by the statistic index of two classes, such as the mean and variance, and used to measure discrimination in one feature dimensionality. A higher J-M distance value indicates better discrimination from the two class sample in one feature dimensionality. The J-M distance equation is:

$$J = 2(1 - e^{-B}) \quad (2)$$

$$B = \frac{1}{8(m_1 - m_2)^2} * \frac{2}{\sigma_1^2 + \sigma_2^2} + \frac{1}{2} ln \frac{\sigma_1^2 + \sigma_2^2}{2\sigma_1\sigma_2} \quad (3)$$

where m_i and σ_i^2 (i = 1 , 2) refer to the mean and variance of a two-classification sample.

The range of the J-M distance is (0-2); 0 means completely mixed, while 2 means completely separated. Because the J-M distance is unable to completely reach 2, the best result is associated with the highest J-M distance.

Since multi-class classification often achieves better than binary classification. We split non-tea class by forest and cropland, and calculate J-M distance between tea and both of them. Table 3 shows the J-M distance results calculated from different features, with results sorted by value.

Table 3 J-M Distances of part features

	J-M distance（tea and corpland）	J-M distance（tea and forest）
Red band mean	0.75	1.05
GLCM Homogeneity all dir	0.44	0.84
GLCM Homogeneity 135	0.35	0.68
Avrage. area represented by segments	0.20	0.72
Stddev of area represented by segments	0.53	0.68
GLCM Homogeneity 45	0.48	0.59
Max. diff.	0.20	0.75

Most features with high J-M distances are different operators based on the Gray-level cooccurrence matrix (GLCM) and Gray-level difference vector (GLDV). These features have strong discrimination but high correlation, which causes high redundancy and low accuracy. Thus, features with strong correlation coefficients should be removed. Pearson's correlation coefficient was utilized to calculate the correlation coefficients, as shown in Eq.4 and 5:

$$r_{ij} = \frac{\sum_{l=1}^{K}\left(x_i^l - \overline{x_i}\right)\left(x_j^l - \overline{x_j}\right)}{\sqrt{\sum_{l=1}^{K}\left(x_i^l - \overline{x_i}\right)^2 \sum_{l=1}^{K}\left(x_j^l - \overline{x_j}\right)^2}} \quad (4)$$

$$\overline{x_i} = \frac{1}{K}\sum_{l=1}^{K} x_i^l \quad (5)$$

where x_i^l refers to the i-th feature of the l-th sample and $\overline{x_i}$ refers to the mean value of the i-th feature.

Features with high correlation coefficients and relatively low J-M distances were removed from the 20 features with the highest J-M distances. The remaining features established a feature set with ten features. (*Table 4*).

In this feature set, the most contributory features were texture features such as GLCM Angular Second Moment (ASM), GLCM homogeneity, GLCM dissimilarity. The texture feature is reflected by the correlation between two pixels at a certain distance and angle. Those features were calculated inside image object, using all pixels inside the image object.The equations of used texture features are shown (Eq.6-10).

$$GLCM_{Homogeneity} = \sum_{i,j=0}^{N-1} P_{i,j}/(1+(i-j)^2) \quad (6)$$

$$GLCM_{ASM} = \sum_{i,j=0}^{N-1} {P_{i,j}}^2 \quad (7)$$

$$GLCM_{Dissimilarity} = \sum_{i,j=0}^{N-1} P_{i,j}|i-j| \quad (8)$$

$$GLCM_{StdDev} = \sqrt{\sum_{i,j=0}^{N-1} P_{i,j}(i,j-u_{i,j})} \quad (9)$$

$$GLDV_{Mean} = \sum_{i,j=0}^{N-1} k(V_k) \quad (10)$$

Because tea has similar spectral characteristics as other vegetation, the use of texture features can highly improve the accuracy and results of tea area extraction. Besides, the geometry features also have strong discrimination. The width of an image object is calculated using the length-to-width ratio. The Stddev of Area Represented by Segments is based on the standard deviation of triangles created by Delaunay triangulation. The number of neighbors calculated by the number of the direct neighbors of an image object. Finally, spectral features such as red band mean and max difference were used to build feature set. The Table 4 shows the best separate achievements in texture features.

Table 4 Correlation coefficients and J-M distances of feature set

	**GLCM homogeneity**	**Width**	**GLDV mean**	**Max. diff**	**GLCM dissimilarity**	**GLCM StdDev**	**Number of neighbors**	**Stddev of area represented by segments**	**Red band mean**	**GLCM ASM**
**J-M distance（tea and forest）**	0.85	0.04	0.21	0.76	0.21	0.09	0.01	0.68	1.06	0.16
**J-M distance（tea and corpland）**	0.44	0.86	0.76	0.20	0.76	0.78	0.91	0.54	0.75	0.89
**GLCM homogeneity**	1.00	0.50	-0.71	0.03	-0.71	-0.64	0.34	0.65	-0.01	-0.42
**Width**	0.50	1.00	-0.63	0.40	-0.63	-0.72	0.81	0.53	-0.49	-0.34
**GLDV mean**	-0.71	-0.63	1.00	-0.43	1.00	0.84	-0.55	-0.49	0.35	0.72
**Max. diff**	0.03	0.40	-0.43	1.00	-0.43	-0.59	0.52	0.00	-0.74	-0.30
**GLCM dissimilarity**	-0.71	-0.63	1.00	-0.43	1.00	0.84	-0.55	-0.49	0.35	0.72
**GLCM StdDev**	-0.64	-0.72	0.84	-0.59	0.84	1.00	-0.67	-0.50	0.53	0.53

**Number of neighbors**	0.34	0.81	-0.55	0.52	-0.55	-0.67	1.00	0.34	-0.58	-0.31
**Stddev of area represented by segments**	0.65	0.53	-0.49	0.00	-0.49	-0.50	0.34	1.00	-0.04	-0.23
**Red band mean**	-0.01	-0.49	0.35	-0.74	0.35	0.53	-0.58	-0.04	1.00	0.19
**GLCM ASM**	-0.42	-0.34	0.72	-0.30	0.72	0.53	-0.31	-0.23	0.19	1.00

Classification method and accuracy assessment

Maximum likelihood algorithm, OOIA-based CART decision tree algorithm, CNN-based algorithm[9] and SVM algorithm were compared using the feature set established by the above methods to extract tea from 2017 image data. In case of CNN-based algorithm, features was obtain by CNN and selected by Gini index, and classifer was built by random forest, following the method proposed by Tang[9]. In case of SVM algorithm, the Radial Basis Function (RBF) was used as kernel function in order to deal with linear inseparable case.

The samples were gathered by field research and visual interpretation. The number and construction of samples were shown in Table 5. We split them for training and testing. So, the confusion matrix was calculated based on 2108 non-tea samples and 303 tea samples obtained by field observation to assess accuracy[18-20]. The best algorithm was chosen by comparing the overall accuracy, kappa coefficients, producer accuracy, and user accuracy to achieve the best performance during the extraction of tea area from multi-temporal image data.

Table 5. Training and validation samples

	Tea	Not tea	
		Forest	Corpland
**Training**	990	2927	2003
**Testing**	303	1254	854

Results

Extraction and result assessment

After the classification and accuracy assessment stage, tea plantation area (*Fig 9,10*) and accuracy (*Table 6*) were obtained. The CNN result achieve relatively accuracy because of over-fitting problem in small sample case. The loss the training loss is converging to 0.01, while the validation loss is still not converging, causing low accuracy in validation set and even low accuracy in test set. The maximum likelihood results had the lowest user accuracy and Kappa coefficients because some other vegetation was mis-classified as tea. This misclassification was successfully minimized by the SVM algorithm, which improved the user accuracy of tea to 87.7%. However, the producer accuracy of tea of SVM results slightly lower then maximum likelihood and CART results. Since pine trees are widespread in the study area; which have similar the texture

with tea, it is difficult to separate this vegetation from tea in optical remote sensing data. Thus, misclassification occurred between these two vegetation types, causing low accuracy, but the method could effectively separate tea from other vegetation, especially wood.

Table 6. Accuracy assessment of 2017 classification result

	Maximum likelihood	CART	CNN	SVM
**Overall accuracy**	86.15%	90.75%	86.7%	93.14%
**Kappa coefficient**	0.67	0.76	0.65	0.81
**Producer accuracy (tea)**	93.28%	88.02%	76.66%	83.81%
**Producer accuracy (other vegetation)**	83.83%	91.63%	90.01%	96.16%
**User accuracy (tea)**	65.28%	77.43%	71.43%	87.7%
**User accuracy (other vegetation)**	97.45%	95.91%	92.2%	94.81%

Fig 9. Tea extraction result. The base map of the LandSat8 OLI images with the following composition:R (4),G (3) and B (2).The LandSat8 OLI images were downloaded from USGS National Map Viewer.

Then, proposed method was utilized to extract tea from three periods in 2014, 2015, and 2016, and the results contained tea-growing areas (*Fig.* 10). To examine the accuracy of the results, the traditional accuracy assessment method was used to generate a confusion matrix (*Table 6*). Some extracted tea areas were in the deep forest, but most tea was normally planted at the edge of a forest and in the neighborhood of the residential area during field research. However, the result accuracy was satisfactory for applications.

Table 7. Accuracy assessment of 2014-2016 SVM classification result

	2014	2015	2016
**Overall accuracy**	90.43%	90.65%	93.14%
**Kappa coefficient**	0.71	0.75	0.81
**Producer accuracy (tea)**	87.24%	73.84%	89%
**Producer accuracy (other vegetation)**	91.16%	97.31%	94.5%
**User accuracy (tea)**	69.23%	91.6%	84.17%
**User accuracy (other vegetation)**	96.6%	90.37	96.31%

Fig 10. Extraction results in (a)2014 (b)2015 and (c)2016. The base map of the LandSat8 OLI images with the following composition:R (4),G (3) and B (2).The LandSat8 OLI images were downloaded from USGS National Map Viewer.

Change detection

The ENVI software was utilized for change detection, while the area was calculated by Arc GIS software for statistical analysis. The spatial variation distribution (*Fig. 11*) and area change situation were analyzed (*Table 7, Fig. 12, 13*). The overall pattern of spatial variation is expansion to the mid-west and east. The tea plantation area sharply increased between 2015 and 2016 and slowly increased between 2014 and 2015. The increasing rate was much slower between 2016 and 2017.

Table 8. Tea plantation area changes during 2014-2017

Time	2014	2015	2016	2017
**Area/acre**	13757.64	14705.58	17765.30	17853.00

Fig 11. Spatial distribution during (a)2014-2015 (b)2015-2016 and (c)2016-2017. The base map of the LandSat8 OLI images with the following composition:R (4),G (3) and B (2).The LandSat8 OLI images were downloaded from USGS National Map Viewer.

Fig 12. Spatial variation from 2014 to 2017

Fig 13. Area change from 2014 to 2017

Discussion

In this study, our primary objective was to analyze and propose a tea plantation mapping method and monitor the tea plantation area in Menghai Town from 2014 to 2017. The optimal segmentation parameters, such as segmentation scale (SS), weight of spectrum (WS1), weight of shape (WS2), weight of smoothness (WS3), and weight of compactness (WC), were selected by calculate and analyze the local variance (LV) and the rate of change (ROC) of image homogeneity. The experiment results indicated that the optimal extraction of tea plantation are achieved when SS is set to 66, the WS is set to 0.5, and the WC is set to 0.7. The SS determines the size of the image object, which means higher SS causing larger image object and missing details. We found that for tea plantation mapping SS can not set too high because of the planting characteristics and vegetation form. WS1 is based on the standard deviation of the spectral colors, while WS2 is based on the deviation of a compact(or smooth) shape. WS1 determines to what degree spectrum influences the segmentation compared to shape, which against WS2. In Menghai town, most tea plantations have regular shape, so high WS2 can achieve better segmentation results. The value of WC gives it a relative weighting against WS3. The plantation form of tea in Menghai town is relatively compact, which make properly higher WC segment tea plantation better[21].

In order to build feature space for image objects to utilize the classification algorithm, SEaTH method is used to calculate the J-M distance of both spectrum and texture features between tea plantation and other vegetations. The experiment results showed that texture features, such as GLCM and GLDV, have the highest J-M distance. Since tea plantation in this area have more regular plantation form which significantly different with other vegetation, texture features are more suitable to separate tea from other vegetation. Besides, the mean value of the red band also have a relatively high J-M distance. Because vegetation coverage in tea plantation area normally lower than other vegetation in this area such as forest, the absorption effect of the red band in tea plantation area is relatively lower than other vegetation, causing high discrimination in the mean value of red band [22].

Then decorrelation method was used to remove the correlated features by calculating the correlation coefficients in order to build feature space without redundancy. The results indicated that although J-M distance of most texture features are high, the correlation coefficients are also high, which causes redundancy. Figure 14 shows the correlation coefficients of some texture features.

Fig 14. Correlation coefficients of texture features

The accuracy of maximum likelihood algorithm, CRAT algorithm, CNN-based method(Tang et al.,2020) and SVM algorithm were evaluated at data in 2017 to find the most suitable algorithm. Besides, the performing of binary classification(tea and other vegetation) and multi-classification(tea, cropland and forest) was assessed. Table 9 shows the accuracy assessment results. SVM with multi-classification achieving 93.14% overall accuracy , 0.81 kappa coefficient and 87.7% user accuracy outperforming other methods. The experiment results also indicates that multi-classification method achieving better accuracy than binary classification method in all methods. Multi-classification CART improves 4.15% of overall accuracy and 9.97% of user accuracy on binary classification, which higher than SVM, because SVM only uses support vector to build classifier, which are more robust than CART in binary classification. Besides, multi-classification CNN slightly improves the accuracy of tea extraction on binary classification CNN, which are 0.01 of kappa coefficient and 4.16% of producer accuracy of tea.

Table 9. Accuracy assessment of 2017 binary classification and multi-classification result

	**Maximum likelihood(binary)**	**Maximum likelihood(multi)**	**CART (binary)**	**CART (multi)**	**CNN (binary)**	**CNN (multi)**	**SVM (binary)**	**SVM (multi)**
**Overall accuracy**	82.5%	86.15%	86.6%	90.75%	86.9%	86.7%	91.94%	93.14%
**Kappa coefficient**	0.60	0.67	0.66	0.76	0.64	0.65	0.77	0.81
**Producer accuracy (tea)**	93.35%	93.28%	86.60%	88.02%	72.5%	76.66%	79.87%	83.81%
**Producer accuracy (other vegetation)**	78.97%	83.83%	86.40%	91.63%	91.6%	90.01%	95.87%	96.16%
**User accuracy (tea)**	59.12%	65.28%	67.46%	77.43%	73.7%	71.43%	86.31%	87.7%
**User accuracy (other vegetation)**	97.32%	97.45%	95.18%	95.91%	91.1%	92.2%	93.6%	94.81%

Then, the best performing algorithm was used to mapping tea plantation at 2014 to 2017. Accuracy assessment was done by confusion matrix and kappa coefficient(*Table 6*). All overall accuracy were above 90%, which proves the effectiveness of proposed method.

Conclusion

Menghai Town of Xishuangbanna Dai Autonomous Prefecture, Yunnan Province, Chian,was chosen as the study area, and GF-1 remotely sensed data from 2014 to 2017 were chosen as the data source. Image texture, spectral and geometry features were

integrated, while feature space was built by separability and thresholds algorithms (SEaTH) with decorrelation. Object-oriented image analysis (OOIA) with SVM algorithm was utilized to extract tea plantation areas. The salient findings of the study are as follows:

- OOIA can successfully suppress noise by object-based classification and improve the effect of extraction.
- The selection of segmentation scale and parameters is a critical factor in OOIA. LV and ROC can be used as criteria to evaluate parameters in order to generate meanful image objects. Scale parameter that is too small causes over segmentation, while a scale that is too large scale mixes different earth surface features in one image object.
- The feature space built by the SEaTH algorithm and decorrelation with texture features can effectively avoid spectral confusion.
- OOIA combined with the SVM algorithm and texture features solves the misclassification and missing-classification issues, yielding a Kappa coefficient up to 0.81 and an overall accuracy of 93.14%, higher than CART algorithm. Multi-classification method achieve better performance than binary classification in most algorithm.
- According to the extraction results, tea plantation areas are mainly distributed in the neighborhoods of residential areas with moderate slopes, because tea must be cultivated in easily drained areas and be convenient for farmers to reach. Based on variation analysis, we found that tea plantations expanded towards areas with sparse forests and moderate slopes.

Expectations

The extraction method of tea plantations based on RS still needs to be discovered. Currently, methods still have flaws in accuracy and efficiency. In consideration of this, we will focus on the following aspect in feature research:

- ESP is utilized to select the segmentation scale, but other parameters still need to be selected based on a visual assessment. The parameter selection method can be improved by automatically selecting the weight of the spectrum and the weight of compactness based on LV to achieve the optimal segmentation result.
- In order to overcome texture and spectral confusion, different data and extraction methods can be used: consider the growing period of tea based on low revisit period RS data; multi-source data fusion such as hyperspectral and high-resolution RS data to analyze the spectral and texture features; integrate with LiDAR data to obtain canopy height and shape information to reduce spectrum fixing; use few sample learning method to overcome overfitting problem of deep learning and CNN.

Acknowledgements

The authors are grateful to the editor and anonymous referees for their valuable comments and helpful suggestions. We wish to thank professor Cheng Wang and Xiaohuang Xi for their advice.

References

1. Li Z, He LQ, Tea and Meteorology. 1st ed. Beijing, China: Meteorological Press; 2005. pp. 25-26.
2. Dihkan M, Guneroglu N, Karsli F. Remote sensing of tea plantations using an SVM classifier and pattern-based accuracy assessment technique. International Journal of Remote Sensing. 2013; 34(23): 8549-8565.
3. Xu WY, Sun R, Jin ZF. Extracting tea plantations based on ZY-3 satellite data. Transactions of the Chinese Society of Agricultural Engineering (Transactions of the CSAE). 2016; 32(Supp.1): 161-168.
4. Rajapakse RS, Tripathi NK, Honda K. Spectral characterization and LAI modelling for the tea (Camellia sinensis (L.) O. Kuntze) canopy. International Journal of Remote Sensing. 2002; 23(18), 3569-3577.
5. He L, Kong F, Shen Z. Artificial Neural Network Ensemble for Land Cover Classification, World Congress on Intelligent Control & Automation 2006.
6. Li, Z, Li, Z. Mapping the Spatial Distribution of Tea Plantations Using High-Spatiotemporal-Resolution Imagery in Northern Zhejiang. Forests. 2019; 10(10): 856.
7. Jun Z, Ziwu P, Hang W, Peijie H, Jiulin S, Fen Q, et al. An Improved Multi-temporal and Multi-feature Tea Plantation Identification Method Using Sentinel-2 Imagery. Sensors. 2019; 19(9): 2087.
8. Mallinis G, Koutsias N, Tsakiri-Strati M, et al. Object-based classification using Quickbird imagery for delineating forest vegetation polygons in a Mediterranean test site. ISPRS Journal of Photogrammetry & Remote Sensing. 2008; 63(2), 237-250.
9. Zixia T, Mengmeng L Xiaoqin W. Mapping Tea Plantations from VHR Images Using OBIA and Convolutional Neural Networks. Remote Sensing. 2020; 12(18): 2935.
10. Wei YH. Remote Sensing Digital Image Processing Tutorial, 2nd ed. Beijing, China: Science Press; 2015. pp. 185-304.
11. Sun JB. Principle and Application of Remote Sensing, 3rd ed. Wuhan, China: Wuhan University Press; 2013. pp. 108-224.
12. Deng SB, Chen QJ, Du HJ. ENVI Remote Sensing Image Processing Method, 2nd ed. Beijing, China: Beijing Higher Education Press; 2014. pp. 132-228,
13. Gonzalez RC, Woods RE, Eddins SL. Digital Image Processing Using MATLAB, 2nd ed.Beijing, China: Publishing House of Electronics Industry; 2009. pp. 135-136.
14. Otsu N. A Threshold Selection Method from Gray-Level Histograms. IEEE Transactions on Systems Man, and Cybernetics. 1979; 9(1), 62-66.
15. Liu JH. Mao ZY. Review of High Spatial Resolution Remote Sensing Image Segmentation Methods. Remote Sensing Information. 2009; 11(6): 95-101.
16. Drǎgu, L, Tiede D, Levick SR. ESP: a tool to estimate scale parameter for multiresolution image segmentation of remotely sensed data. International Journal of Geographical Information Science. 2010; 24(6): 859-871.
17. Zhang J. Classification and Feature Selection on High-dimensional and Small-sampling Data. 2014; Hefei University of Technology.
18. Dong LY, Xing LX, Pan J. Vegetation Classification in Hyper-spectral Image with CART Decision Tree. Journal of Jilin University Information Science Edition. 2013; 1: 83-89.
19. Jiang HW, Ren HQ, Qin XF. Auto Interpretation and Accuracy Evaluation of Images Based on eCognition. Bulletin of Surveying and Mapping. 2015; 10, 81-84.
20. Sven N, Gunter M. Object-Based Image Analysis and Treaty Verification. Springer Netherlands. 2008; 51-62.
21. Benz UC, Hofmann P, Willhauck G. Multi-Resolution, Object-Oriented Fuzzy Analysis of Remote Sensing Data for GIS-Ready Information. ISPRS Journal of Photogrammetry & Remote Sensing. 2004; 58: 239–258.

22. Henchiri M, Liu Q, Essifi B. Spatio-Temporal Patterns of Drought and Impact on Vegetation in North and West Africa Based on Multi-Satellite Data. Remote Sensing. 2020; 12(23): 3869.

Supporting Information

S1 File. 2014TeaExtractionResult.
S2 File. 2015TeaExtractionResult.
S3 File. 2016TeaExtractionResult.
S4 File. 2017TeaExtractionResult.

Click here to access/download;Figure;Fig1.tif

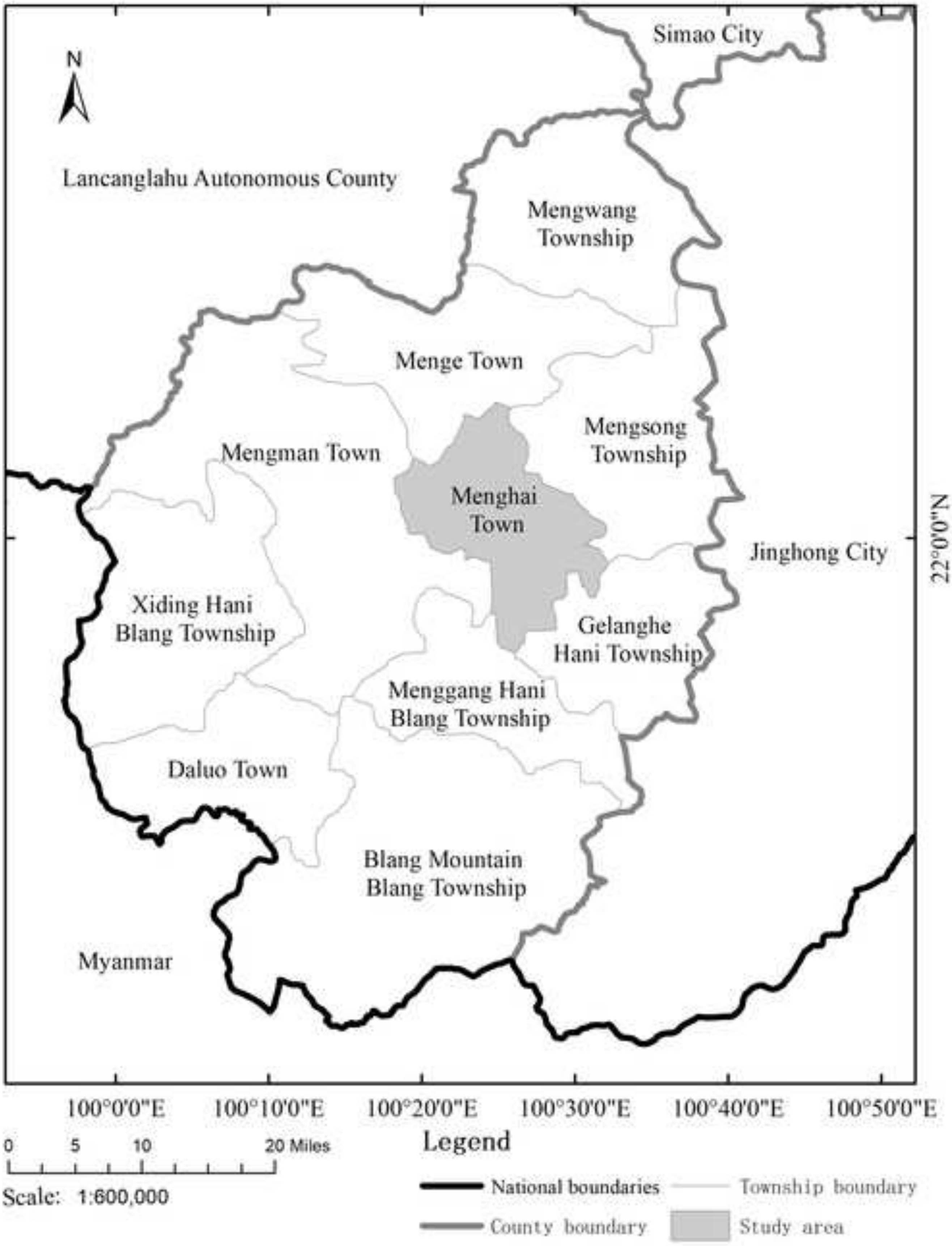

Click here to access/download;Figure;Fig2.tif

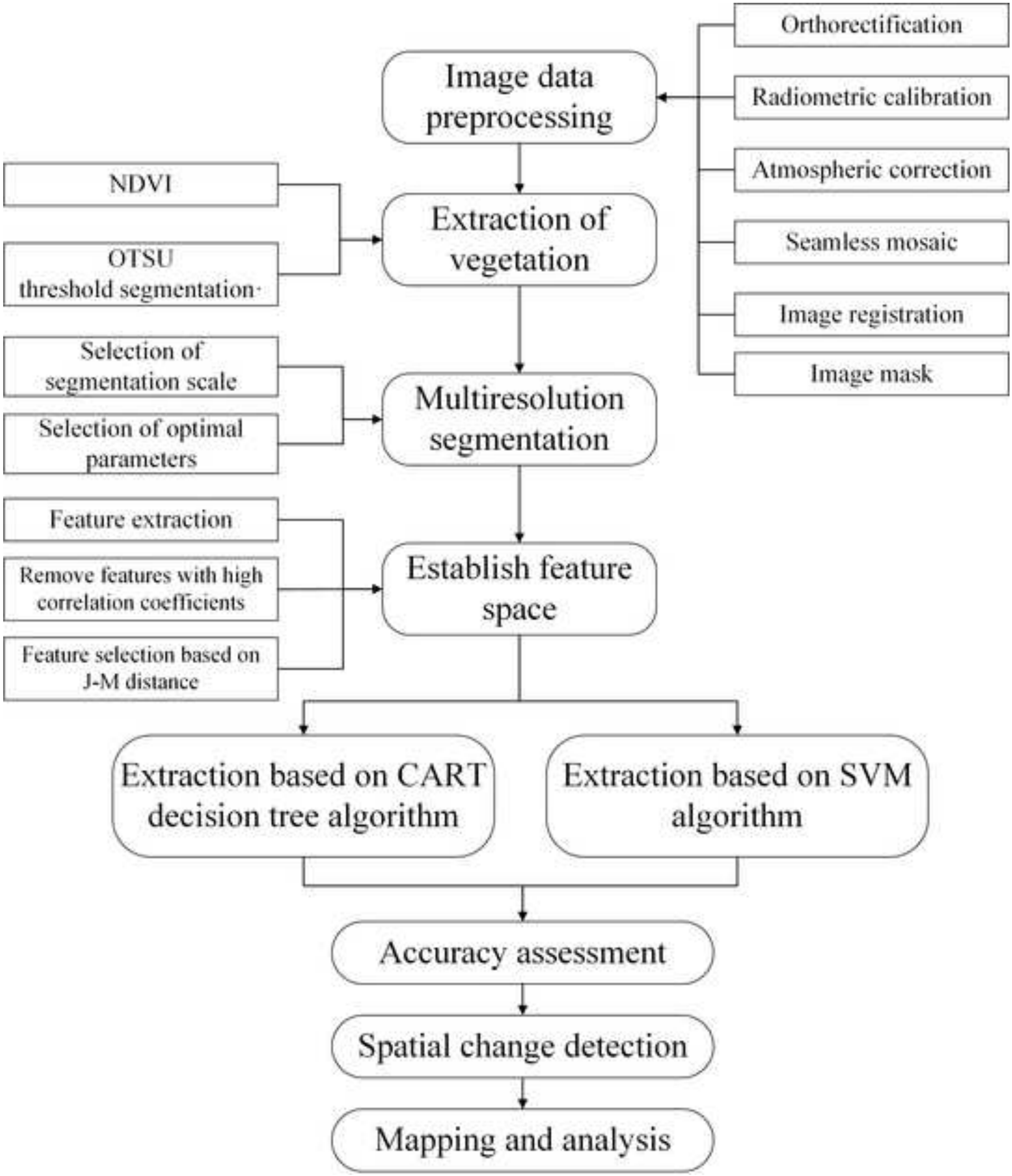

Click here to access/download;Figure;Fig3.tif

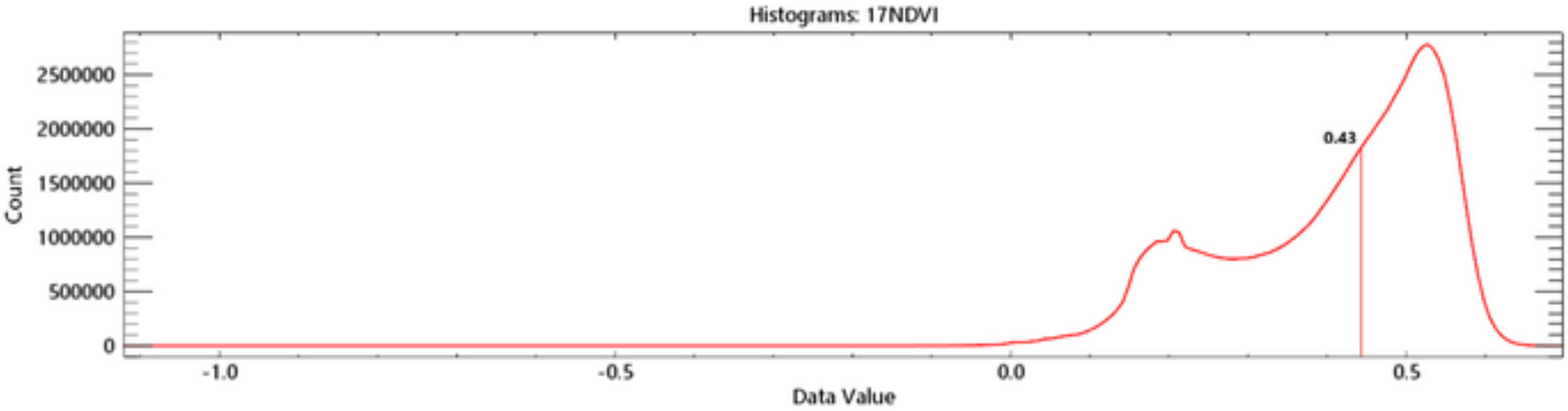

Click here to access/download;Figure;Fig5.tif

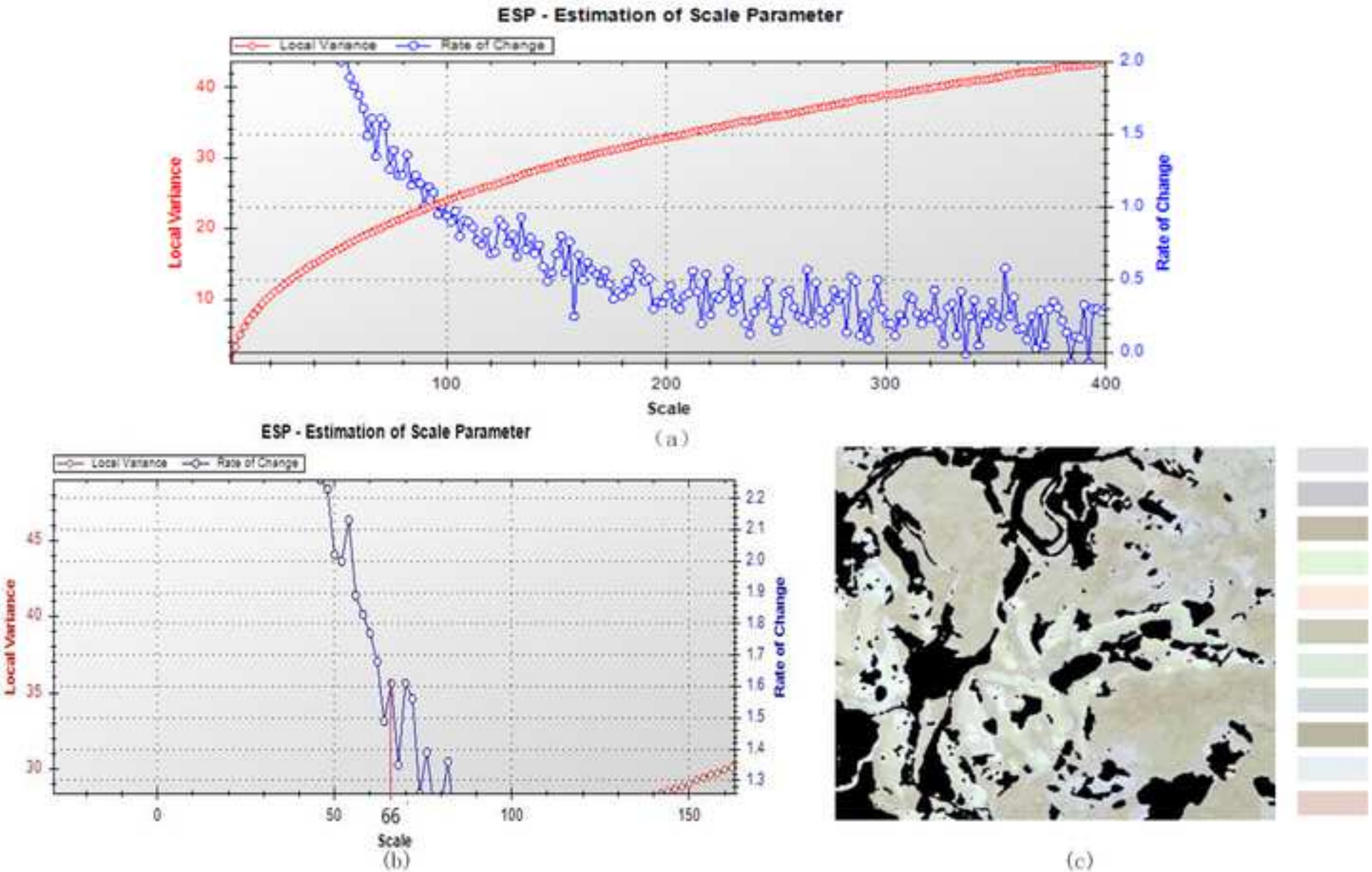

Click here to access/download;Figure;Fig6.tif

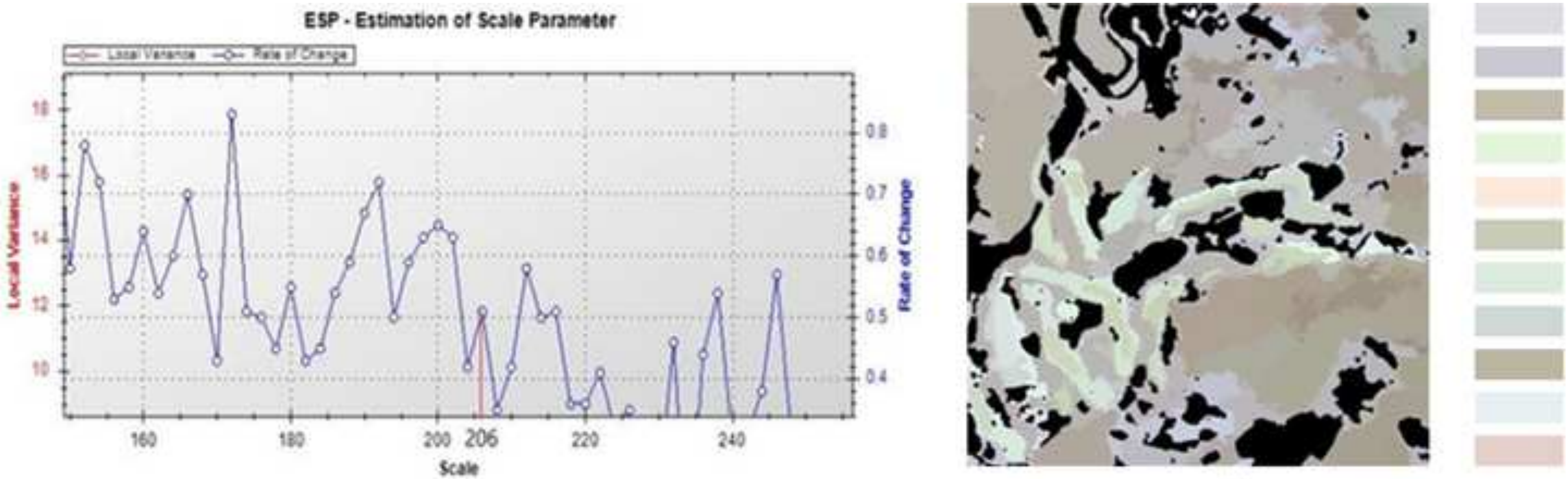

(a)

(b)

Click here to access/download;Figure;Fig7.tif

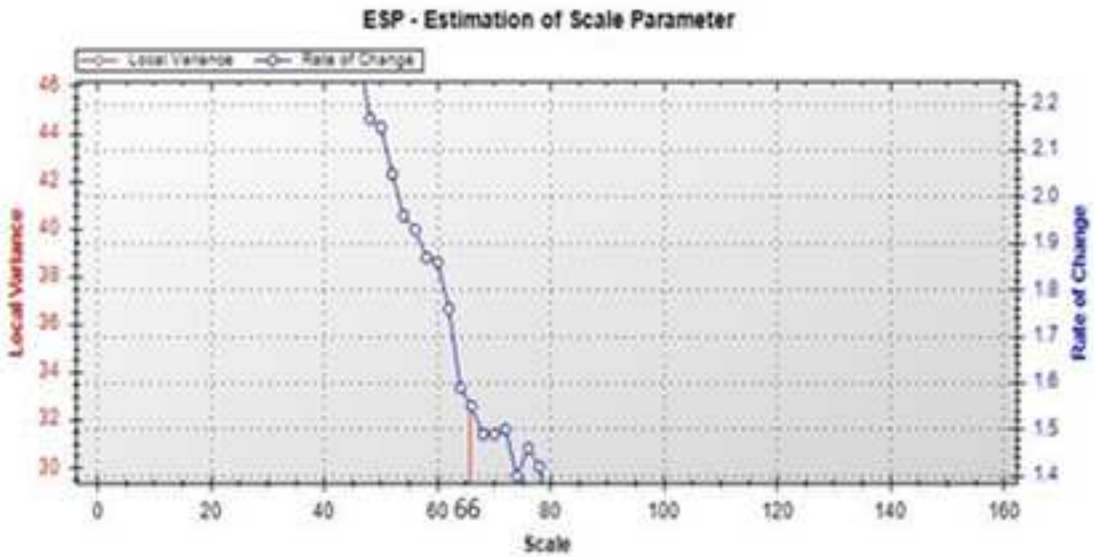

(a)

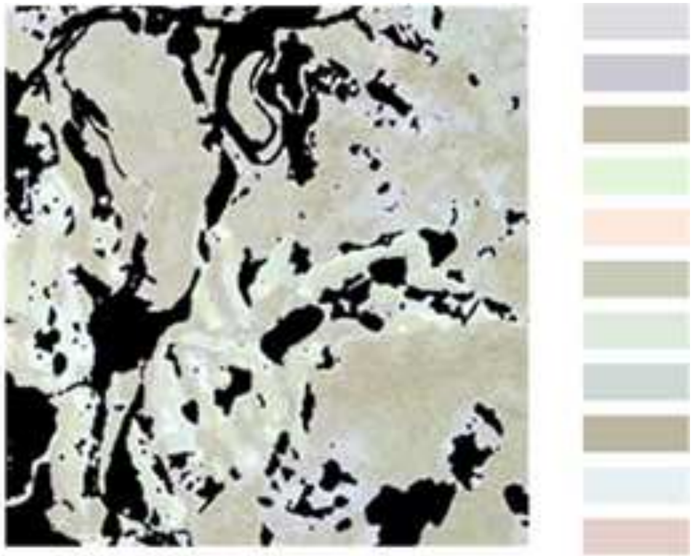

(b)

Click here to access/download;Figure;Fig8.tif

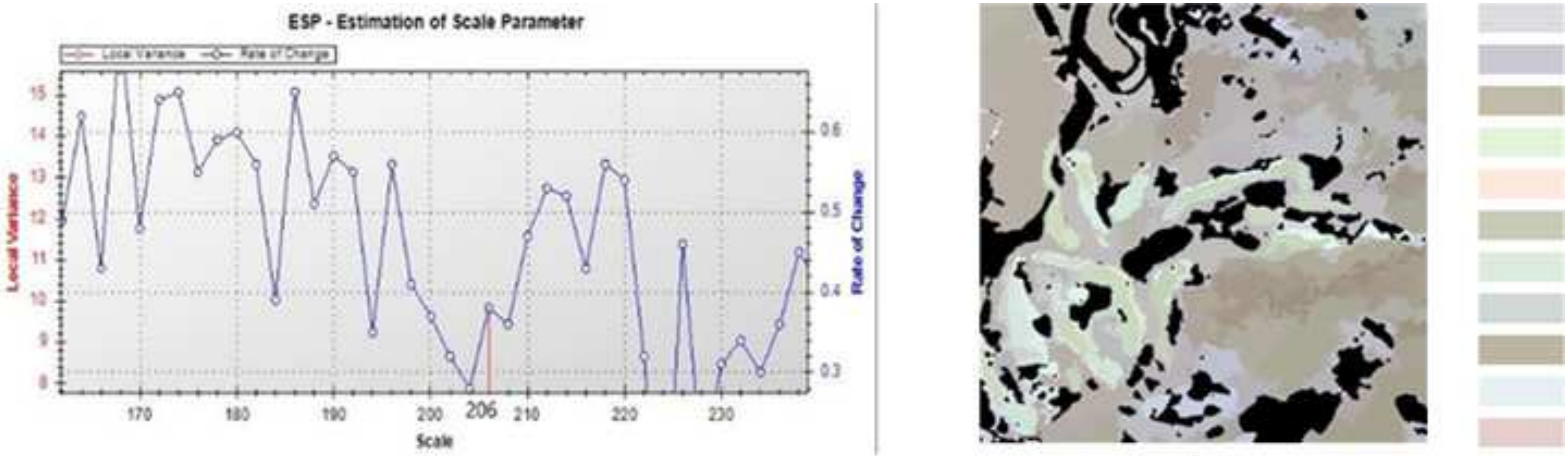

(a) (b)

Click here to access/download;Figure;Fig4.tif

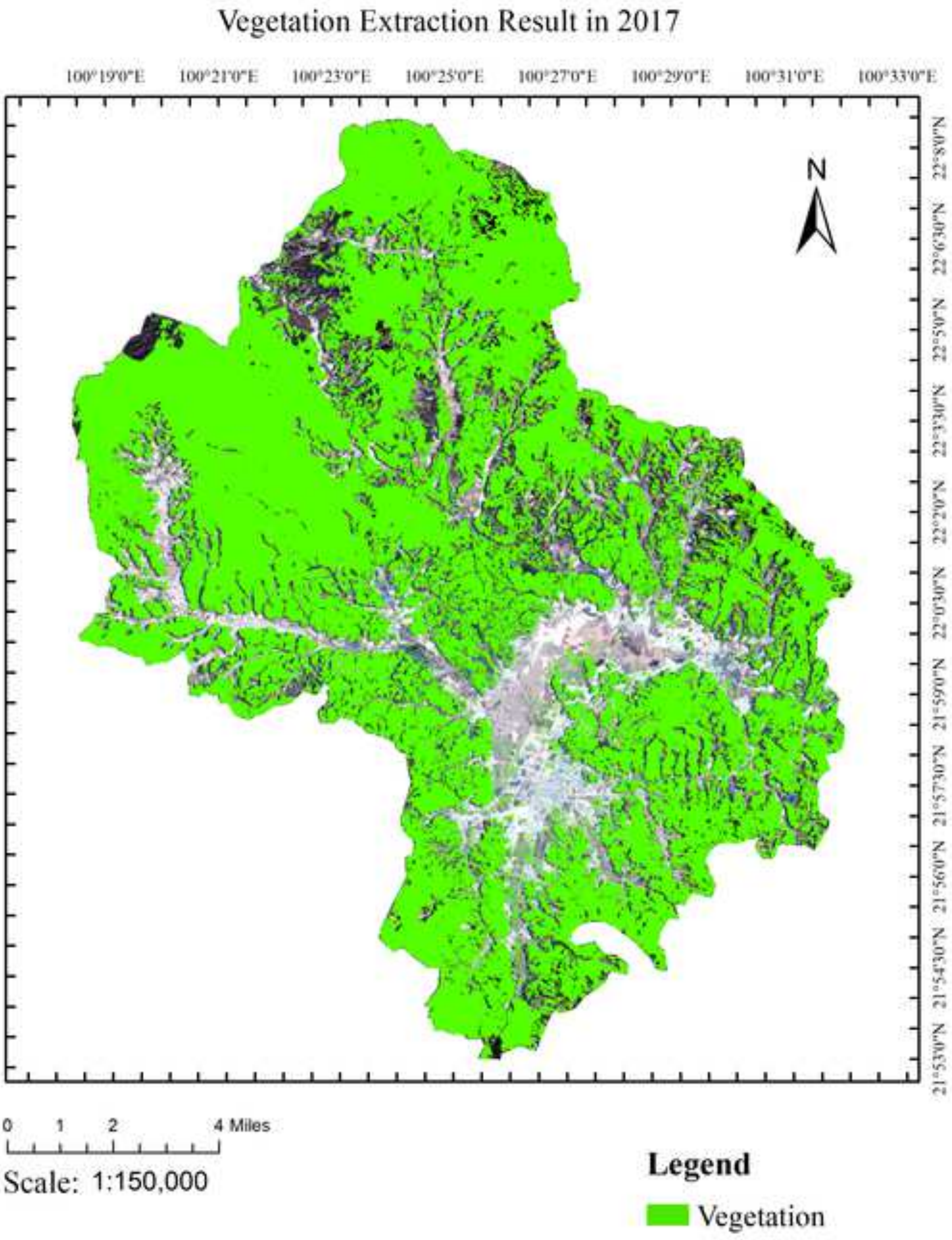

Click here to access/download;Figure;Fig9.tif

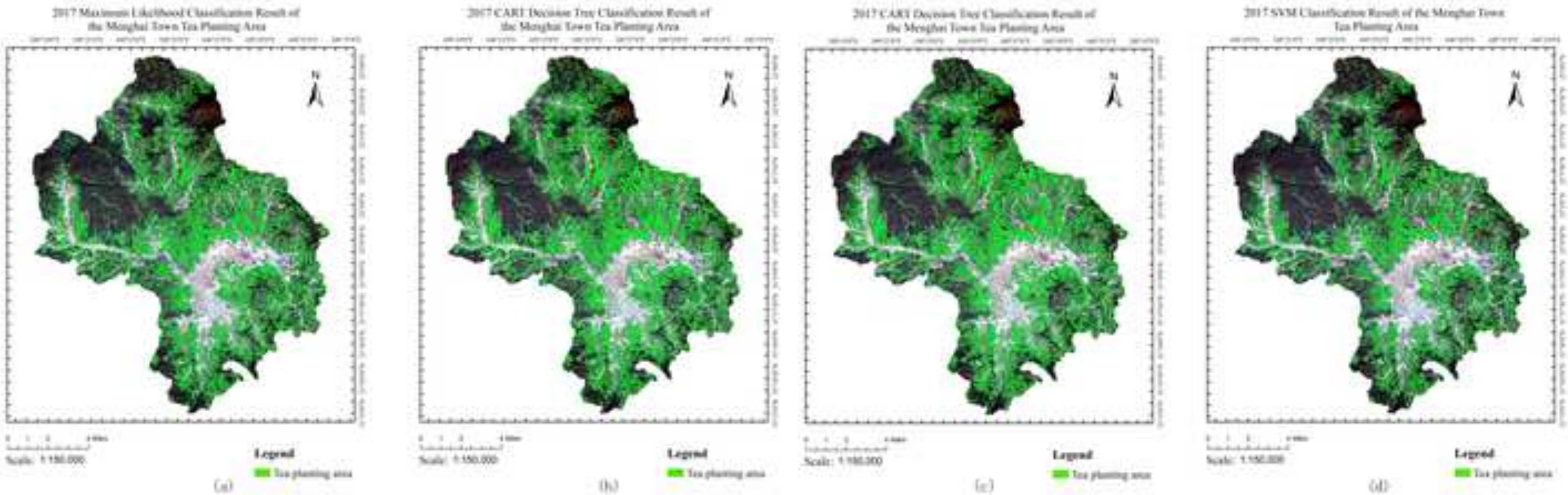

Click here to access/download;Figure;Fig10.tif

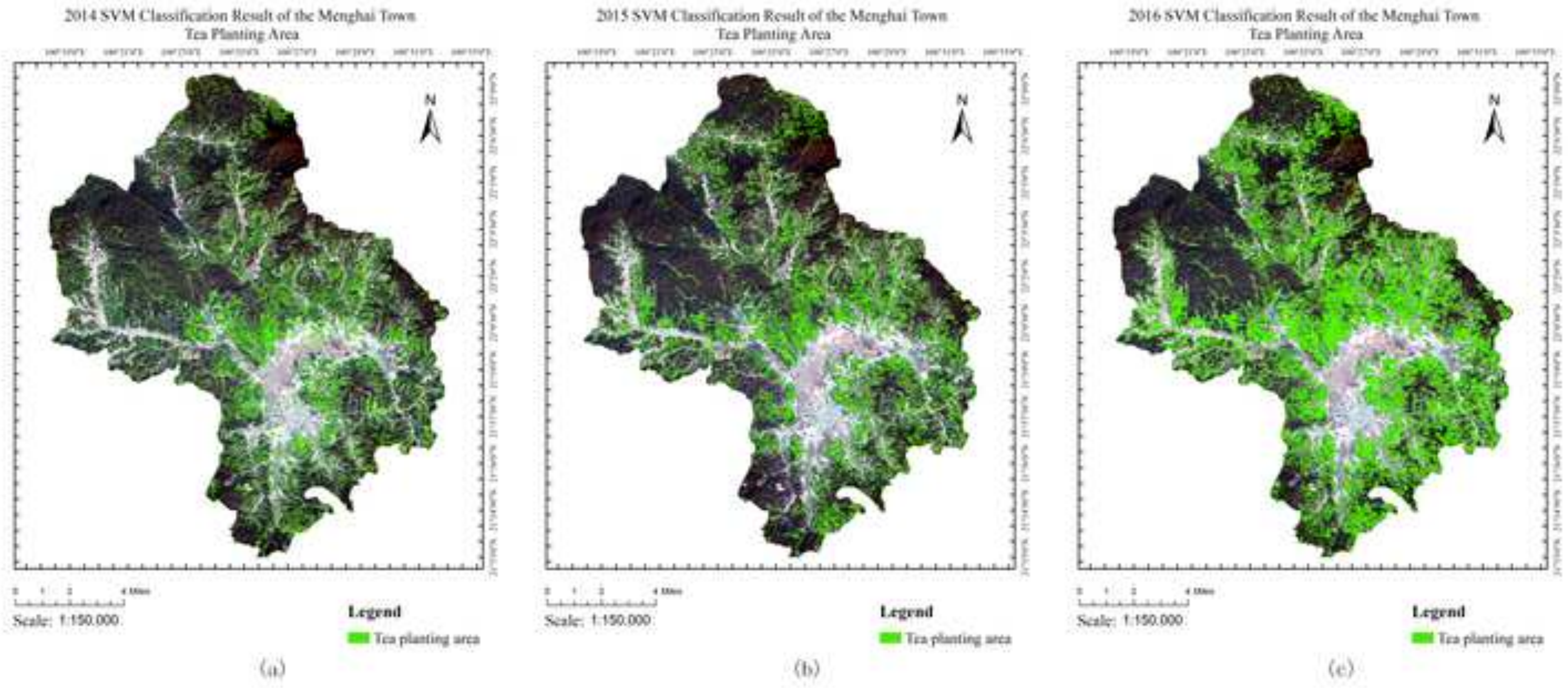

Click here to access/download;Figure;Fig11.tif

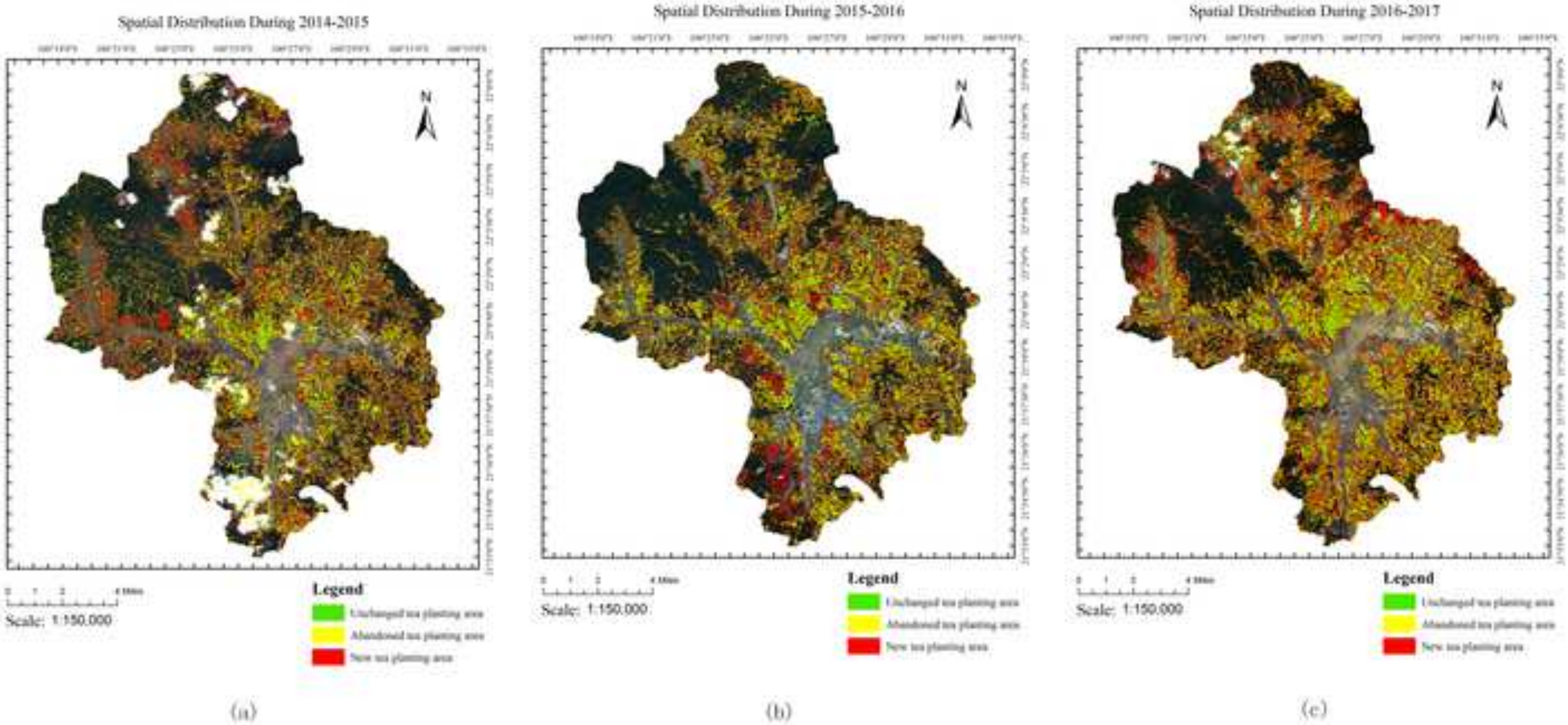

Click here to access/download;Figure;Fig12.tif

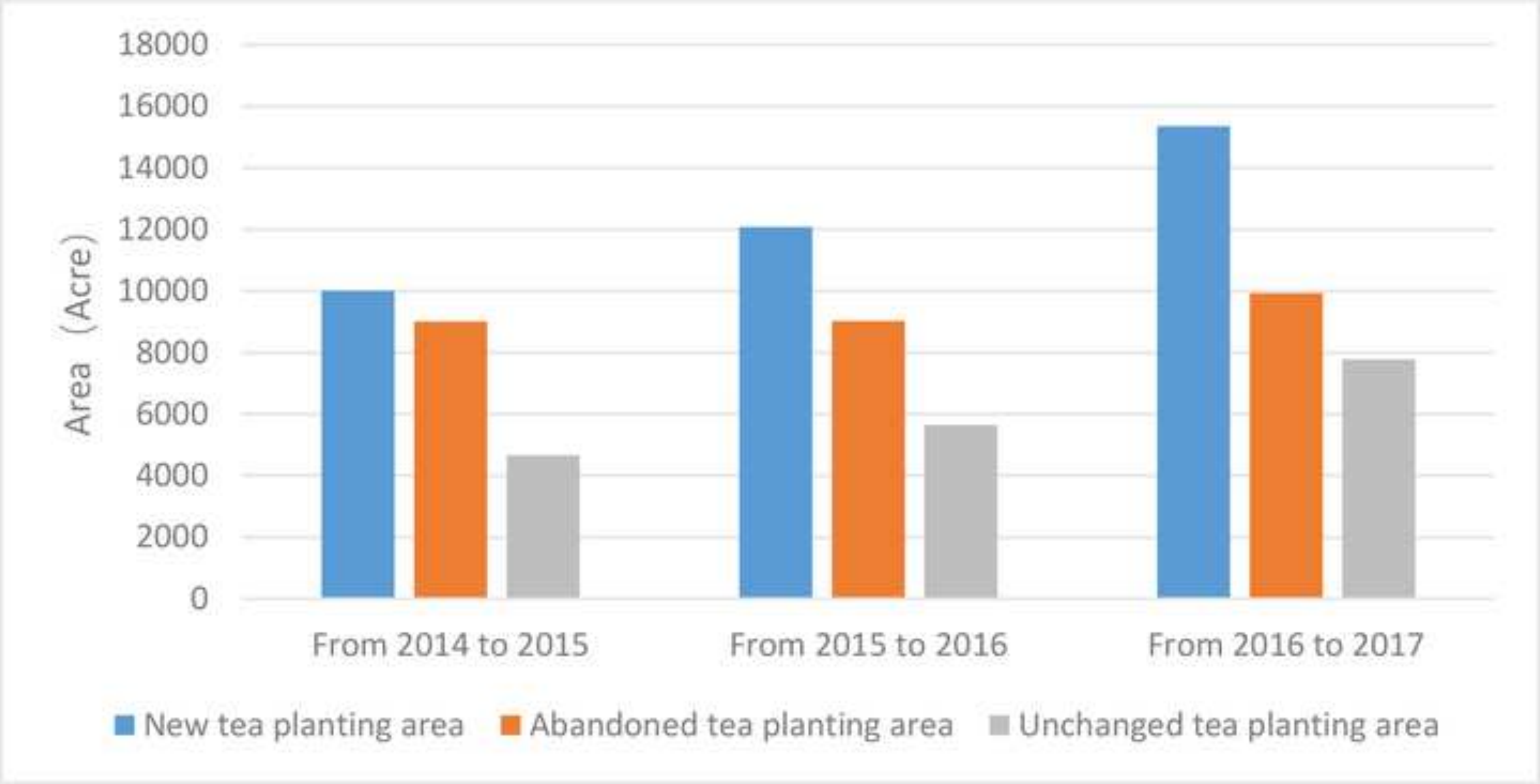

Click here to access/download;Figure;Fig13.tif

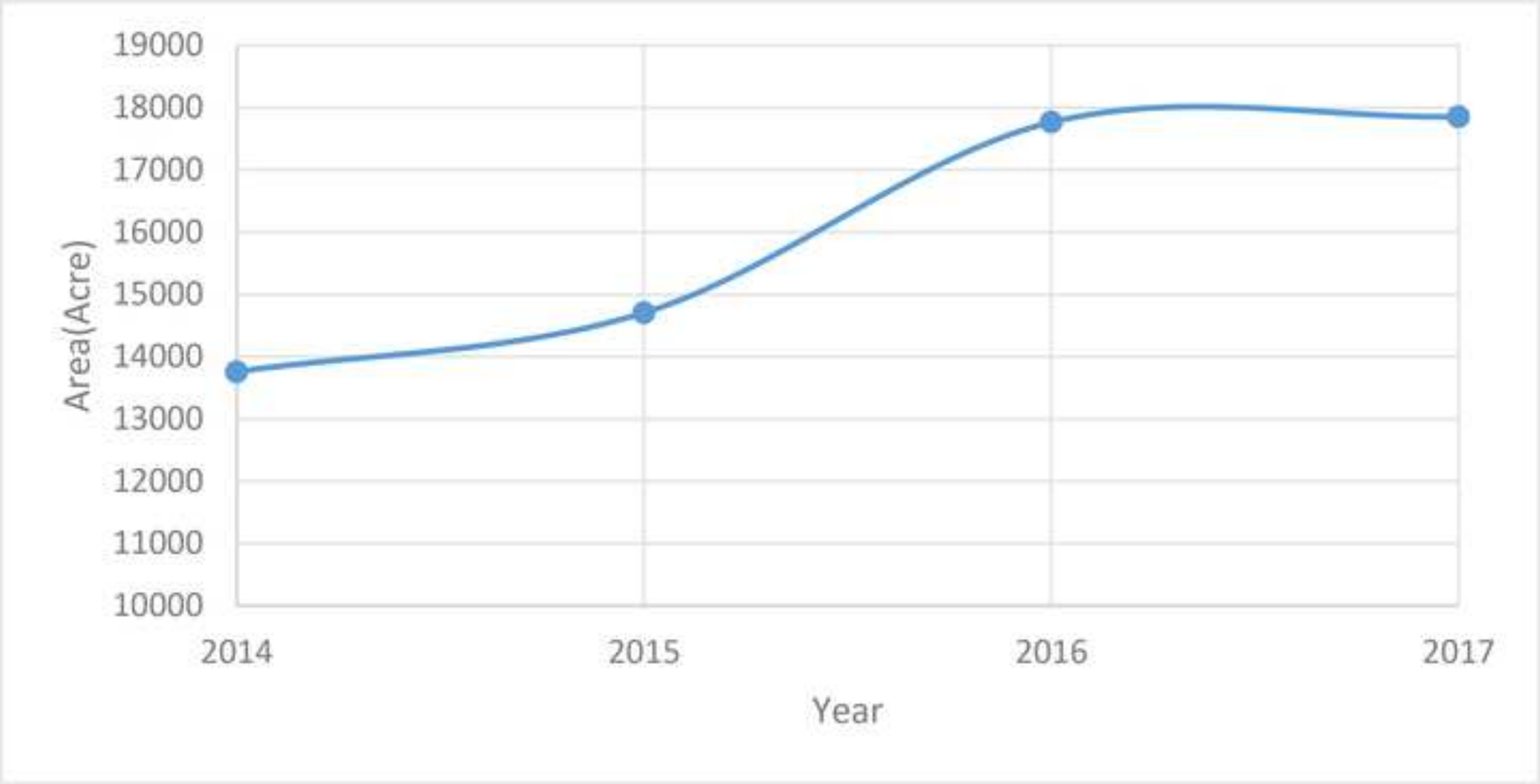

Click here to access/download;Figure;Fig14.tif

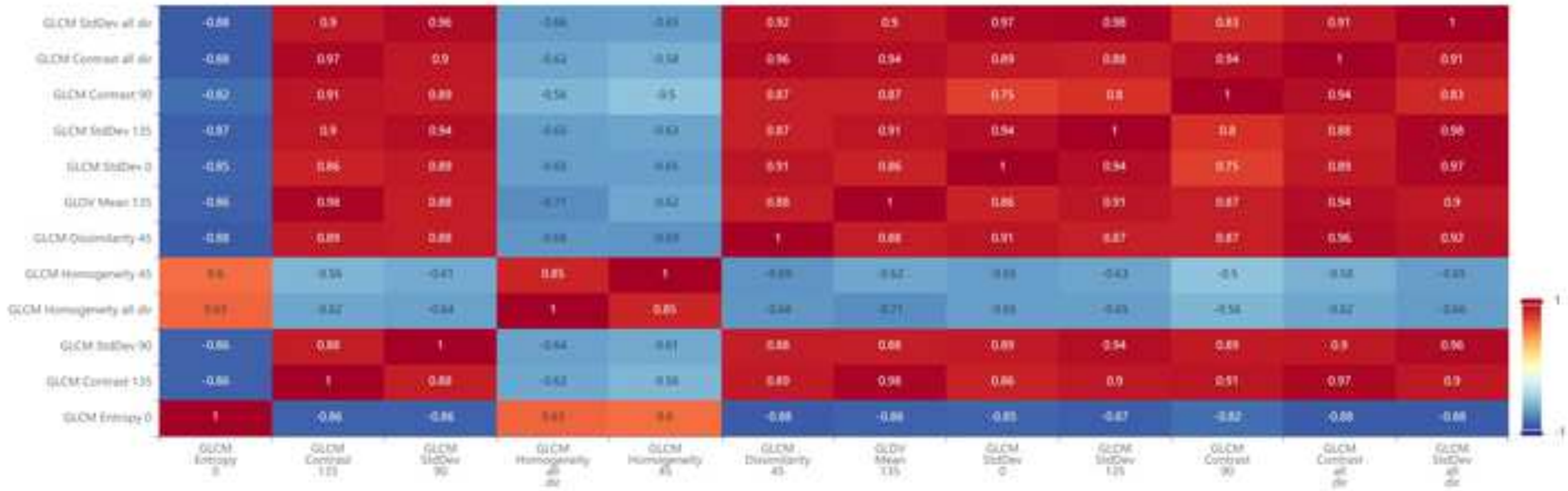

Click here to access/download
Supporting Information - Compressed/ZIP File Archive
2014TeaExtractionResult.zip

Click here to access/download
Supporting Information - Compressed/ZIP File Archive
2015TeaExtractionResult.zip

Click here to access/download

Supporting Information - Compressed/ZIP File Archive

2016TeaExtractionResult.zip

Click here to access/download
Supporting Information - Compressed/ZIP File Archive
2017TeaExtractionResult.zip

Revised Manuscript with Track Changes

Style Definition: 05 Main Section Title: Font: 18 pt
Style Definition: 09 References Title: Font: 18 pt, Left
Style Definition: 10 Figure Titles: Font: Not Italic, Indent: First line: 0.15"

Mapping Pu'er tea plantations from GF-1 images using Object-Oriented Image Analysis (OOIA) and Support Vector Machine (SVM)

LIANG, L.[1,2,3]– WANG, J. L.[1,2,3,*] –DENG, F.[1,2,3]–KONG, D.Y.[1,2,3]

1Faculty of Geography, Yunnan Normal University, Kunming 650500, China
2Key Laboratory of Resources and Environmental Remote Sensing for Universities in Yunnan, Kunming 650500, China
3Remote Sensing Research Laboratory, Center for Geospatial Information Engineering and Technology of Yunnan Province, Kunming 650500, China
* Corresponding author
E-mail: jlwang@ynnu.edu.cn

Mapping Pu'er tea plantations from GF-1 images using Object-Oriented Image Analysis (OOIA) and Support Vector Machine (SVM)

~~Mapping Pu'er Tea Plantations From Gf-1 Images Using Ooia And CART~~

LIANG, L.[1,2,3]– WANG, J. L.[1,2,3,]* –DENG, F.[1,2,3]–KONG, D.Y.[1,2,3]

[1] *Faculty of Geography, Yunnan Normal University, Kunming 650500, China*

[2] *Key Laboratory of Resources and Environmental Remote Sensing for Universities in Yunnan, Kunming 650500, China*

[3] *Remote Sensing Research Laboratory, Center for Geospatial Information Engineering and Technology of Yunnan Province, Kunming 650500, China*

** Corresponding author*
e-mail: jlwang@ynnu.edu.cn

Abstract~~.~~

Tea is~~-~~ the most popular drink worldwide, and China is the largest producer of tea. Therefore, tea is an important commercial crop in China, playing a significant role in domestic and foreign markets. It is necessary to make accurate and timely maps of the distribution of tea plantation areas ~~to~~ for plantation management and decision making. In the present study, the town of Menghai in the Xishuangbanna Dai Autonomous Prefecture, Yunnan Province, China, was chosen as the study area, and GF-1 remotely sensed data from 2014-2017 were chosen as the data source. Image texture, spectral and geometrical ~~geometry~~ features were integrated, while feature space was built by SEparability and THresholds algorithms~~separability and thresholds algorithms~~ (SEaTH) with decorrelation. Object-Oriented Image Analysis~~Object-oriented image analysis~~ (OOIA) with a Support Vector Machine ~~classification and regression tree~~ (~~CART~~SVM) algorithm was utilized to map tea~~-~~ plantation areas. The overall accu~~-~~racy and Kappa coefficient of the proposed method were 9~~2.00~~3.14% and 0.81, respectively, ~~4~~3.61% and 0.05~~.~~, 6.99~~14~~% and 0.1~~54~~, 6.44% and 0.16 better than the results of ~~the maximum likelihood~~CART method, ~~and~~ Maximum likelihood method and CNN based method. The tea plantation area increased by 4,095.36 acre~~14 km2~~ from 2014 to 2017, while the fastest-growing period is 2015 to 2016~~, with slight fluctuations during this period~~.

Keywords: *Pu'er tea, OOIA, Feature selection, ~~Decision tree~~SVM, Change detection*

Introduction

Tea is the most popular drink worldwide ~~(Li et al., 2005)~~[1], and the plantation and production of tea play~~-~~ an important role in agricultural economies and country development. Furthermore, the excessive plantation of tea has many negative impacts on environ~~-~~ments such as landslides and water and soil loss ~~(Dihkan et al.,2013)~~[2]; therefore, the continuous moni~~-~~toring of tea-growing areas has significant importance.

Traditionally, related departments use ~~manual~~ visual interpretation methods to monitor tea distribu~~-~~tion from remote sensing image, but these methods are inefficient, have a low

Formatted: Font: 18 pt

Formatted: Font: 12 pt, (Asian) Chinese (PRC), Do not check spelling or grammar

Formatted: Indent: First line: 0.5"

Formatted: Font: 12 pt, (Asian) Chinese (PRC), Do not check spelling or grammar, Not Superscript/ Subscript

Formatted: Font: 12 pt, (Asian) Chinese (PRC), Do not check spelling or grammar

Formatted: Font: 12 pt, (Asian) Chinese (PRC), (Other) English (United Kingdom), Do not check spelling or grammar

precision, are expensive, and are challenged by the need for spatially continuous monitoring over large areas.

Remote sensing technology can obtain spatially continuous information on earth surfaces[3] (Xu et al.,2016), which has plenty of applications in tea spatial distribution extraction and monitoring, because this technology has wide data coverage, fast data-update speeds, and low costs.

However, different tea growing areas have spectral differences due to canopy structure and traits, plantation size, crop health and growing period, enlarging the within-class distance of tea in spectral features. The spectral reflectance of tea is close to that of other vegetation, such as fruit trees and bushes, narrowing the class distance. Therefore, the spectral mixing phenomenon makes it extremely difficult to extract tea plantation distributions (Rajapakse et al.,2002)[4]. For example, He[5] used an artificial neural network (ANN) to classify vegetation using Landsat Thematic Mapper (TM) images found that the extraction of bushes and tea had a relatively low accuracy, but the extraction of other vegetation achieved a high accuracy. One effective method of plantation distribution extraction is to use spatial information based on texture features. For instance, Xu[3] built a decision tree based on the texture features of anisotropic strength to extract the tea plantation distribution in mountain areas, using ZY-3 satellite data. Dihkan[2] extracted the Gabor texture feature from multispectral airborne digital image to train a support vector machine classifier (SVM) and extract tea plantations distribution in black sea areas, achieving an overall accuracy above 90%.For example, He (He et al.,2006) used an artificial neural network (ANN) to classify vegetation and found that the extraction of bushes and tea had a relatively low accuracy, but the extraction of other vegetation achieved a high accu-racy. One effective method of plantation distribution extraction is to use spatial in-formation based on texture features. For instance, Xu (Xu et al.,2016) built a decision tree based on the texture features of anisotropic strength to extract the tea plantation distribution in mountain areas. Dihkan (Dihkan et al.,2013) uses the Gabor texture feature to train a support vector machine classifier (SVM) and extract tea plantations distribution in black sea areas, achieving an overall accuracy above 90%. Another method is using time series analysis to extract temporal feature based on regular annual growth cycle of tea. For example, Li (Li et al.,2019)[6] used high-spatiotemporal-resolution VENμS images to extract temporal and spectral features, -and utilized decision tree to mapping tea plantation. Zhu (Zhu et al.,2019)[7] inte-grates multi-temporal features to train a random forest classifier based on middle spital resolution satellite remote sensing data- Sentinel-2. However, high-temporal-resolution means either low spatial resolution such as MODIS or small coverage such as VENμS, which not able to map large extensive area. Due toBecause of data limitations, most previous studies based on mid/low-resolution satellite remote sensing data were unable to effectively extract texture features in tea-growing areas, while few high spatial resolutionhigh-resolution-based studies used aerial remote sensing data, with high costs and a relatively small coverage.

High spatial resolutionHigh-resolution satellite remote sensing data contain sufficient targets details, advancing the effect of the inside texture and structure features of targets. However, too exhaustive details may cause salt-and-pepper noise in information extraction and image classification. OOIA solves this problem effectively. For instance, Mallinis (Mallinis et al.,2008) [8] used OOIA to extract the distribution of vegetation based on Quickbird satellite remote sensing data, which had a spatial resolution of 0.61 m and improved the classification accuracy. Tang (Tang et al.,2020)[9] integrated OOIA

with convolutional neural network to mapping tea plantation, achieving comparable results and can be used with limited training samples.

Menghai town in the Xishuangbanna Dai Autonomous Prefecture, Yunnan Prov~~-~~ince, was chosen as the study area, and GF-1 remotely sensed data from 2014 to ~~-~~2017 were chosen as the data source. In this study, OOIA and texture features were com~~-~~bined, while features selected by SEaTH, the CART algorithm and ~~a maximum likeli-hood~~SVM algorithm were compared to find the best way to extract the tea plantation area and analyze the plantation distribution and changes. In that case, the change trends of tea plantations can be analyzed to assist relevant departments in decision making.

Study area and data source

Study area

Menhai county is ~~located~~ in the southwestern part of Yunnan province, the southwest corner of China. The longitude of this county is between 99°56′E ~ 100°41′E, and the latitude is between 21°28′N ~ 22°28′N (Fig. 1). The study area has a tropical monsoon climate, with 1136-1513 ml precipitation per year. The annual average tem~~-~~perature is approximately 18.9℃ - 22.6℃, which is extremely suitable for tea growth, making this area one of the original plantations of "Pu Er tea". As of 2017, the overall tea plantation area was approximately 710 thousand acres, making the tea industry the largest and most profitable industry in Menghai County. Menghai town is ~~located~~ in eastern Menghai County, which is the political, economy, and cultural center of Menghai County. The area of this town is 365.38 km^2, ~~,,~~ of which 45.3 km^2~~-~~, is tea plan~~-~~tation area, which show~~make~~s the tea industry plays a serious role in the local economy.

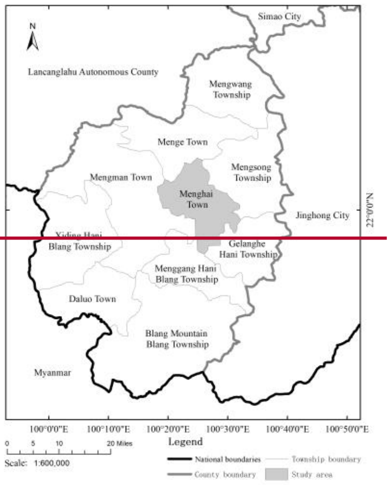

Fig~~ure~~ 1. Study area

Data source and preprocessing

Formatted: Font: 16 pt, Not Italic

Formatted: Font: Not Italic

Formatted: Font: Bold

Formatted: Font: 16 pt, Not Italic

The PMS(Panchromatic and multispectral sensors) data products from 2014 to -2017, which were obtained from the GF-1 satellite data, were used in the present study (Table 1). The preprocesses were supported by ENVI 5.3 software and ~~comprised~~ consisting of seven steps. Primarily, radiometric calibration~~radiation collaboration~~, FLAASH~~flash~~ atmosphere correction, orthorectification~~orthographical correction~~, image pan sharping and image mosaicking were completed. Then, 2017 data were selected as the base image to register the other three images by im-age-to-image registering ~~(Wei et al.,2015, Sun et al.,2013, Deng et al.,2014, Deng et al.,2014)~~[10-13]. The RMS of the image registering result was con-trolled below 1 pixel. Finally, vector data of Menghai town were used to mask the image to obtain four scene images of Menghai town.

Table 1. GF-1 ~~SATELLITE~~ satellite PMS ~~DATA~~ data ~~PARAMETERS~~parameters

Parameters	2 m pan/8 m multiresolution ~~Parameters~~	
**Spectral range**	Pan	0.45-0.90 μm
	Multispectral	0.45-0.52 μm
		0.52-0.59 μm
		0.63-0.69 μm
		0.77-0.89 μm
**Spatial resolution**	Pan	2 m
	Multispectral	8 m
**Swath**	60 km (2 camera)	
**Temporal (sway)**	4 day	
**Temporal (not sway)**	41day	

Methods

Research workflow

A six-step method was used to analyze the tea plantations. First, the normalized difference vegetation index (NDVI) was calculated from the preprocessed image. Second, OTSU algorithm was used to select best NDVI threshold to separate non-vegetation area from vegetation area. The ~~T~~third step were selection of optimal segmentation and~~as~~ multiresolution segmentation~~, but the optimal parameters first had to be obtained~~. The fourth step was building feature space by the selection of best features using SEaTH. The fifth step was a comparison between the ~~performance~~ performing of SVM method, ~~the maximum likelihood algorithm and~~ CART method, Maximum likelihood method and CNN based method~~the CART decision tree~~ to determine the best method. Finally, the best-performing method was utilized to extract tea distribution information from the rest of the image and analyze the changing situation (*Fig. 2*).

Formatted: Font: Not Italic

Formatted: Normal, Indent: First line: 0", Space Before: 12 pt, After: 6 pt

Formatted: Font: 11 pt, Bold, (Asian) Chinese (PRC), (Other) English (United Kingdom), Do not check spelling or grammar

Formatted Table

Formatted: Font: Bold, (Asian) Chinese (PRC), (Other) English (United Kingdom), Do not check spelling or grammar

Formatted: Font: Bold, (Asian) Chinese (PRC), (Other) English (United Kingdom), Do not check spelling or grammar

Formatted: Font: Bold, (Asian) Chinese (PRC), (Other) English (United Kingdom), Do not check spelling or grammar

Formatted: Font: Bold, (Asian) Chinese (PRC), (Other) English (United Kingdom), Do not check spelling or grammar

Formatted: Font: Bold, (Asian) Chinese (PRC), (Other) English (United Kingdom), Do not check spelling or grammar

Formatted: Font: Bold, (Asian) Chinese (PRC), (Other) English (United Kingdom), Do not check spelling or grammar

Formatted: Font: Bold, (Asian) Chinese (PRC), (Other) English (United Kingdom), Do not check spelling or grammar

Formatted: Font: Bold, (Asian) Chinese (PRC), (Other) English (United Kingdom), Do not check spelling or grammar

Formatted: Font: Bold, (Asian) Chinese (PRC), (Other) English (United Kingdom), Do not check spelling or grammar

Formatted: Font: Bold, (Asian) Chinese (PRC), (Other) English (United Kingdom), Do not check spelling or grammar

Formatted: Font: 16 pt, Not Italic

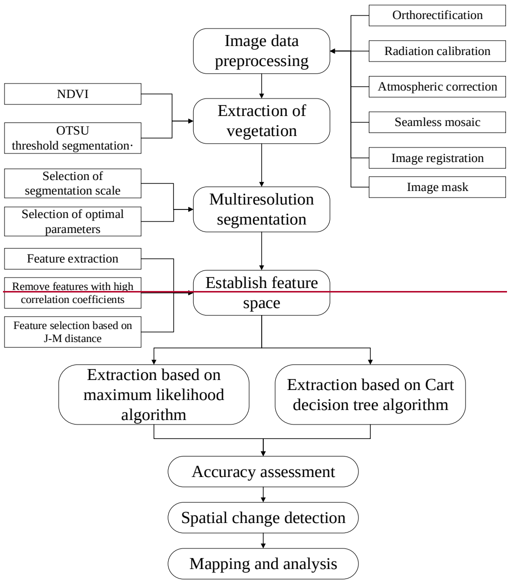

Fig~~ure~~ 2. Research workflow

Extraction of vegetation

The NDVI is a well-performing index to indicate vegetation conditions on the surface. This index is obtained by the spectral reflectance of available light and near infrared between 400 and ~~700~~ 860 nm because vegetation has special reflectance and absorption characteristics in th~~ei~~se bands. The NDVI is calculated as follows:

$$NDVI = \frac{NIR-R}{NIR+R} \qquad (~~Eq.~~1)$$

where NIR is the spectral reflectance of near infrared channel near 860μm, while R is the spectral reflectance of red channel near 660μm.

Because vegetation areas contain tea plantation areas, vegetation areas can be easily distinguished from non-vegetation areas by the NDVI threshold. In that case, the selection of the best-performing threshold is the most crucial stage.

The OTSU algorithm was proposed by Otsu ~~(Otsu et al.,1979)~~[14], a Japanese researcher, in 1979, for image binarization, which was widely used to select the best threshold for grayscale segmentation in digital image processing. In this algorithm, the image is split~~ed~~ into two classes, frontward and backward, by selecting the histogram value for segmentation that makes the variance highest between the two classes. In other words, this algorithm calculates the lowest point between two classifications in the histogram.

NDVI was calculated from preprocessed image data from 2014 to 2017 to select the optimized threshold (*Table 2*). Figure 3 shows the histogram of NDVI in 2017. The histogram can be considered ~~to~~ havinge two peak forms, which makes the OTSU algorithm suitable. Figure 4 shows the results of the vegetation extraction for 2017, which achieved a quite accurate performance.

Table 2. Optimal thresholds in 2014-2017

Formatted: Font: 16 pt, Font color: Black, English (United States)

Formatted: 06 Main Text Format

Formatted: Font: 16 pt

Formatted: Normal, Indent: First line: 0", Space Before: 12 pt, After: 6 pt

Formatted: Font: 11 pt, Bold, (Asian) Chinese (PRC), (Other) English (United Kingdom), Do not check spelling or grammar

year	2014	2015	2016	2017
threshold	0.44	0.42	0.53	0.43

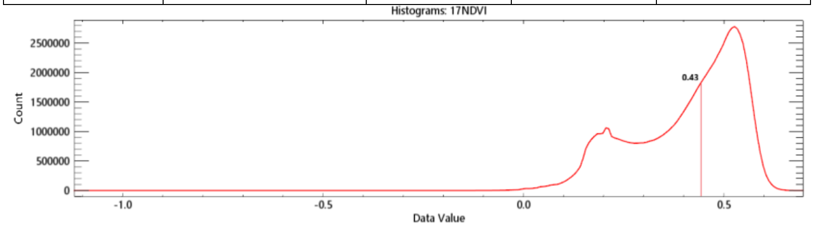

Figure 3. NDVI histogram in 2017

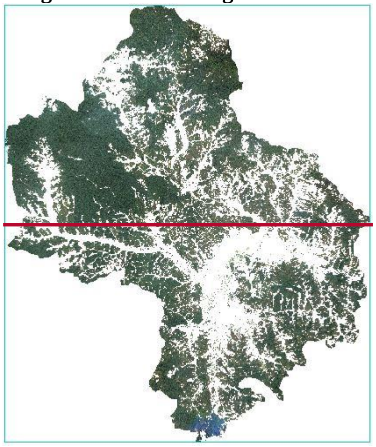

Figure 4. 2017 V~~v~~egetation extraction results. The base map of the LandSat8 OLI images with the following composition:R (4),G (3) and B (2).The LandSat8 OLI images were downloaded from USGS National Map Viewer.

Selection of optimal parameters

To take advantage of the high-resolution feature and neighbor relationship of GF-1 satellite data and prevent noise, a multiresolution segmentation method, which combines neighbor pixels based on spectrum and shape to create image objects, was utilized~~(Liu et al.,2009)~~[15]. In this stage, the selection of segmentation scale (SS), weight of spectrum (WS1), weight of shape (WS2), weight of smoothness (WS3), and weight of compactness (WC) controlled the performance of segmentation.

The estimation of scale parameters (ESP) is an optimal parameter selection method based on local variance (LV) and the rate of change (ROC) of image homogeneity~~(Drăgu et al.,2010)~~[16]. LV is the average of the neighborhood standard deviation in the entire image. Along with the increase in SS, the inner homogeneity of the image object increased, and heterogeneity with other image objects decreased, causing the LV to rise and a slower change rate. When the optimal scale was achieved, the image objects had the highest similarity with earth surface features in reality, LV peaked, and ROC increased gradually. After visual assessment, the size and shape of the results were the most similar to the tea plantation area.

Formatted: Font: Bold
Formatted Table
Formatted: Font: Not Bold
Formatted: Font: 16 pt, Font color: Black, English (United States)
Formatted: 06 Main Text Format
Formatted: Font: 16 pt

In addition to the SS, the WS1 and WS2 control the segmentation results. WS2 also considers WS3 and WC. WS3 and WC are used to control the smoothness of the image object border and separation.

Based on the idea that the control variable is optimal, all SS and other parameters were traversed to integrate ESP and visual assessment to select optimal parameters. The results indicate that the optimal SS was 66, the optimal WS was 0.5, and the optimal WC was 0.7 (*Fig. 5*).

When a larger SS than the optimal SS was used (*Fig. 6*), e.g., 206, the area was too large, and details were missing, which can cause low accuracy in the classification step. When the SS was overlarge, the ROC of LV performing violent fluctuated, which is not the optimal parameter.

When a lower WC was used (*Fig. 7*), the shape of the image object was too regular, and the border was too smooth, which was not the same as reality (*Fig. 7 (b, c)*) and had a negative impact on segmentation. The optimal parameters resulted in the most complete extraction of tea plantation information, with distinct borders.

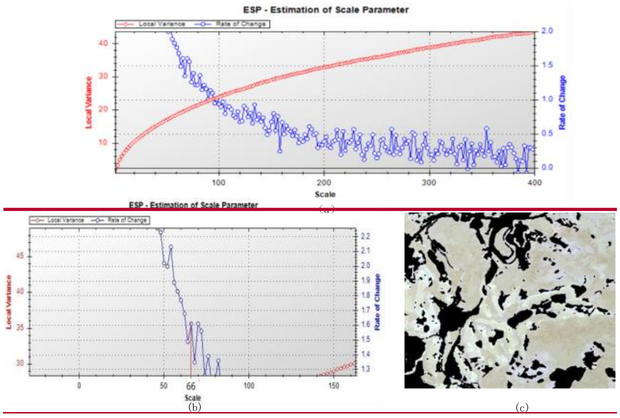

Figure 5. Multiresolution segmentation (SS=66, WS_1=0.5, WC=0.7): (a) variance curve, (b) optimal segmentation scale and (c) image object

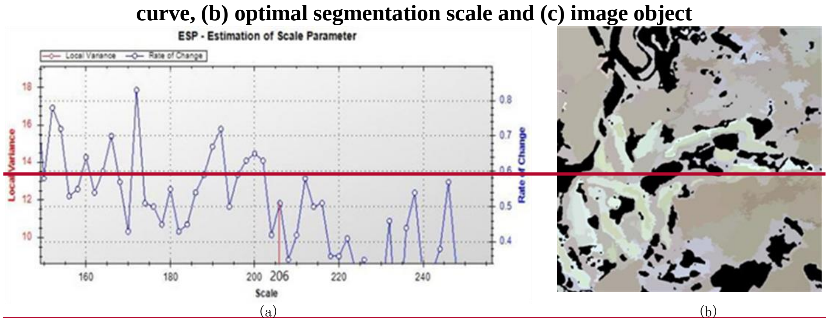

Figure 6. Multiresolution segmentation (SS=206, WS_1=0.5, WC=0.7): (a) variance curve and (b) image object

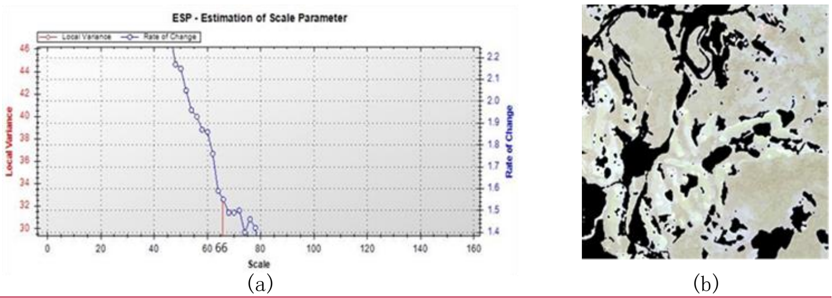

(a) (b)

Figure 7. Multiresolution segmentation (SS=66, WS_1=0.5, WC=0.1): (a) variance curve and (b) image object

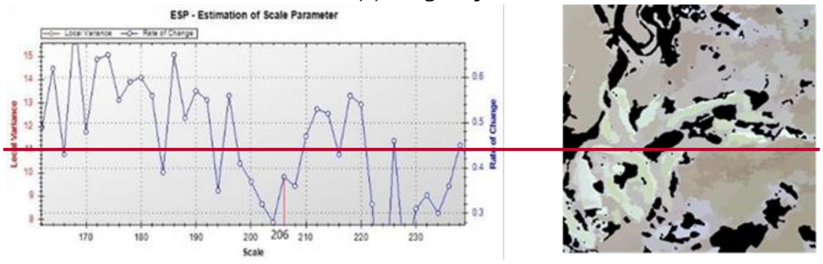

(a) (b)

Figure 8. Multiresolution segmentation (SS=206, WS_1=0.5, WC=0.1): (a) variance curve and (b) image object

Feature space establishment

The eCognition software offers hundreds of features, including spectrum, shape, texture, context, and spatial relationship features. The multidimensional feature space can solve the phenomenon caused by one-dimensional spectrum extraction. However, too many features can cause dimensional disasters, which damage calculation efficiency and accuracy~~(Zhang et al.,2014)~~[17]. Therefore, high efficiency and accuracy are crucial factors for the best selection from abundant features that have high discrimination and low redundancy.

The SEaTH algorithm is a feature selection method based on Jeffries Matusita(J-M) distance~~(Zhang et al.,2018)~~[17], which is calculated by the statistic index of two classes, such as the mean and variance, and used to measure discrimination in one feature dimensionality. A higher J-M distance value indicates better discrimination from the two ~~-~~class sample in one feature dimensionality. The J-M distance equation is:

$$J = 2(1 - e^{-B}) \quad (\text{~~Eq.~~}2)$$

$$B = \frac{1}{8(m_1 - m_2)^2} * \frac{2}{\sigma_1^2 + \sigma_2^2} + \frac{1}{2} ln \frac{\sigma_1^2 + \sigma_2^2}{2\sigma_1\sigma_2} \quad (\text{~~Eq.~~}3)$$

where m_i and σ_i^2 (i = 1 , 2) refer to the mean and variance of a two-classification sample.

The range of the J-M distance is (0-2); 0 means completely mixed, while 2 means completely separated. Because the J-M distance is unable to completely reach 2, the best result is associated with the highest J-M distance.

Formatted: Font: 16 pt, Not Italic, English (United States)

Formatted: 06 Main Text Format, Line spacing: single

Formatted: Font: 16 pt, Not Italic

Formatted: Normal, Indent: First line: 1 ch, Line spacing: Multiple 1.05 li

Since multi-class classification often achieves better than binary classification. We split non-tea class by forest and cropland, and calculate J-M distance between tea and both of them. Table 3 shows the J-M distance results calculated from different features, with results sorted by value.

Table 3 J-M Distances of part features

	J-M distance（tea and corpland）	J-M distance（tea and forest）
Red band mean	0.75	1.05
GLCM Homogeneity all dir	0.44	0.84
GLCM Homogeneity 135	0.35	0.68
Avrage. area represented by segments	0.20	0.72
Stddev of area represented by segments	0.53	0.68
GLCM Homogeneity 45	0.48	0.59
Max. diff.	0.20	0.75

Feature name	J-M distance
GLCM_Ang_135°	1.80
GLCM_Ang_90°	1.64
GLCM_Ang_45°	1.63
GLCM_Ang_180°	1.62
GLDV_Ang_135°	1.20
GLDV_Ang_90°	1.13
GLDV_Ang_45°	1.05
GLCM_Hom	0.74
Mean_Layer_red	0.68
GLCM_Mean	0.66

Most features with high J-M distances are different operators based on the Gray-level cooccurrence matrix (GLCM) and Gray-level difference vector (GLDV). These features have strong discrimination but high correlation, which causes high redundancy and low accuracy. Thus, features with strong correlation coefficients should be removed. Pearson's correlation coefficient was utilized to calculate the correlation coefficients, as shown in Eq.4 and 5:

$$r_{ij} = \frac{\sum_{l=1}^{K}\left(x_i^l - \overline{x_i}\right)\left(x_j^l - \overline{x_j}\right)}{\sqrt{\sum_{l=1}^{K}\left(x_i^l - \overline{x_i}\right)^2 \sum_{l=1}^{K}\left(x_j^l - \overline{x_j}\right)^2}} \qquad \text{(Eq.4)}$$

$$\overline{x_i} = \frac{1}{K}\sum_{l=1}^{K} x_i^l \qquad \text{(Eq.5)}$$

where x_i^l refers to the i-th feature of the l-th sample and $\overline{x_i}$ refers to the mean value of the i-th feature.

Features with high correlation coefficients and relatively low J-M distances were removed from the 20 features with the highest J-M distances. The remaining features established a feature set with four ten features., i.e., two texture features, one spectral feature and one geometry feature (*Table 4*).

In this feature set, the most contributory features was were texture features such as GLCM Angular Second Moment (ASM), GLCM homogeneity, GLCM dissimilarity., which was a function of pixel distance and angle. The texture feature is reflected by the

correlation between two pixels at a certain distance and angle. Those features were calculated inside image object, using all pixels inside the image object.The equations of ~~GLCM Mean and GLCM ASM~~used texture features ~~equations~~ are shown (Eq.6~~- and Eq.7~~10).

$$GLCM_{Homogeneity} = \sum_{i,j=0}^{N-1} P_{i,j}/(1+(i-j)^2) \qquad (6)$$

$$GLCM_{ASM} = \sum_{i,j=0}^{N-1} P_{i,j}{}^2 \qquad (7)$$

$$GLCM_{Dissimilarity} = \sum_{i,j=0}^{N-1} P_{i,j}|i-j| \qquad (8)$$

$$GLCM_{StdDev} = \sqrt{\sum_{i,j=0}^{N-1} P_{i,j}(i,j-u_{i,j})} \qquad (9)$$

$$GLDV_{Mean} = \sum_{i,j=0}^{N-1} k(V_k) \qquad (10)$$

Because tea has similar spectral characteristics as other vegetation, the use of texture features can highly improve the accuracy and results of tea area extraction. Besides, the geometry features also have strong discrimination. The width of an image object is calculated using the length-to-width ratio. The Stddev of Area Represented by Segments is based on the standard deviation of triangles created by Delaunay triangulation. The number of neighbors calculated by the number of the direct neighbors of an image object. Finally, spectral features such as red band mean and max difference were used to build feature set. The Table 4 shows the best separate achievements in texture features.

Table 4 Correlation coefficients and J-M distances of feature set

	GLCM homogeneity	Width	GLDV mean	Max. diff	GLCM dissimilarity	GLCM StdDev	Number of neighbors	Stddev of area represented by segments	Red band mean	GLCM ASM
J-M distance (tea and forest)	0.85	0.04	0.21	0.76	0.21	0.09	0.01	0.68	1.06	0.16
J-M distance (tea and corpland)	0.44	0.86	0.76	0.20	0.76	0.78	0.91	0.54	0.75	0.89
GLCM homogeneity	1.00	0.50	-0.71	0.03	-0.71	-0.64	0.34	0.65	-0.01	-0.42
Width	0.50	1.00	-0.63	0.40	-0.63	-0.72	0.81	0.53	-0.49	-0.34
GLDV mean	-0.71	-0.63	1.00	-0.43	1.00	0.84	-0.55	-0.49	0.35	0.72
Max. diff	0.03	0.40	-0.43	1.00	-0.43	-0.59	0.52	0.00	-0.74	-0.30
GLCM dissimilarity	-0.71	-0.63	1.00	-0.43	1.00	0.84	-0.55	-0.49	0.35	0.72
GLCM StdDev	-0.64	-0.72	0.84	-0.59	0.84	1.00	-0.67	-0.50	0.53	0.53

Number of neighbors	0.34	0.81	-0.55	0.52	-0.55	-0.67	1.00	0.34	-0.58	-0.31
Stddev of area represented by segments	0.65	0.53	-0.49	0.00	-0.49	-0.50	0.34	1.00	-0.04	-0.23
Red band mean	-0.01	-0.49	0.35	-0.74	0.35	0.53	-0.58	-0.04	1.00	0.19
GLCM ASM	-0.42	-0.34	0.72	-0.30	0.72	0.53	-0.31	-0.23	0.19	1.00

	~~GLCM 90°mean~~	~~Area standard deviation~~	~~Red band mean~~	~~GLCM ASM~~
~~J-M distance~~	~~0.66~~	~~0.65~~	~~0.68~~	~~1.80~~
~~GLCM 90°mean~~	~~1~~	~~0.09~~	~~-0.44~~	~~-0.85~~
~~Area standard deviation~~	~~0.09~~	~~1~~	~~0.15~~	~~-0.08~~
~~Red band mean~~	~~-0.44~~	~~0.15~~	~~1~~	~~0.15~~
~~GLCM ASM~~	~~-0.85~~	~~-0.08~~	~~0.15~~	~~1~~

Classification method and accuracy assessment

~~The~~ Maximum likelihood algorithm, OOIA-based CART decision tree algorithm, CNN-based algorithm~~(Tang et al.,2020)~~[9] and ~~maximum likelihood~~SVM algorithm were compared using the feature set established by the above methods to extract tea from 2017 image data. In case of CNN-based algorithm, features was obtain by CNN and selected by Gini index, and classifer was built by random forest, following the method proposed by Tang~~(Tang et al.,2020)~~[9]. In case of SVM algorithm, the Radial Basis Function (RBF) was used as kernel function in order to deal with linear inseparable case.

The samples were gathered by field research and visual interpretation. The number and construction of samples were shown in Table 5. We split them ~~by 80/20~~ for training and ~~validation~~testing. So, ~~T~~the confusion matrix was calculated based on ~~929~~ 2108 non-tea samples and ~~255~~ 303 tea samples obtained by field observation to assess accuracy~~(Dong et al.,2013, Jiang et al.,2015)~~[18-20]. The best algorithm was chosen by comparing the overall accuracy, k~~K~~appa coefficients, producer accuracy, and user accuracy to achieve the best performance during the extraction of tea area from multi-temporal image data.

Table 5. Training and validation samples

	Tea	Not tea	
		Forest	Corpland
Training	990	2927	2003
~~Validation~~Testing	303	1254	854

Results

Extraction and result assessment

After the classification and accuracy assessment stage, tea plantation area (*Fig 9,10*) and accuracy (*Table ~~5~~6*) were obtained. The CNN result achieve relatively accuracy because of over-fitting problem in small sample case. The loss the training loss is converging to 0.01, while the validation loss is still not converging, causing low accuracy in validation set and even low accuracy in test set. The ~~maximum likelihood~~maximum likelihood results had the lowest ~~producer~~ user accuracy and Kappa coefficients because some other vegetation was mis-classified as tea. This misclassification was successfully minimized by the ~~CART~~ SVM algorithm, which improved the ~~producer~~ user accuracy of tea to ~~84~~87.7%. However, the producer accuracy of tea of SVM results slightly lower then maximum likelihood and CART results. ~~However~~Since~~,~~ pine trees are widespread in the study area; ~~because~~ which have similar the texture with ~~of these trees is quite similar as that of~~ tea, it is difficult to separate this vegetation from tea in optical remote sensing data. Thus, misclassification occurred between these two vegetation types, causing low ac-curacy, but the method could effectively separate tea from other vegetation, especially wood.

Table ~~6~~5~~.~~ Accuracy assessment of 2017 classification result

~~classifier~~	Maximum likelihood	CART	CNN	SVM
**Overall accuracy**	86.15%	90.75%	86.7%	93.14%
**Kappa coefficient**	0.67	0.76	0.65	0.81
**Producer accuracy (tea)**	93.28%	88.02%	76.66%	83.81%
**Producer accuracy (other vegetation)**	83.83%	91.63%	90.01%	96.16%
**User accuracy (tea)**	65.28%	77.43%	71.43%	87.7%
**User accuracy (other vegetation)~~User accuracy (tea)~~**	97.45%	95.91%	92.2%	94.81%

Formatted: 05 Main Section Title, Left, Indent: First line: 0 ch, Line spacing: single

Formatted: Font: 16 pt, Not Italic, English (United States)

Formatted: 06 Main Text Format, Line spacing: single, Tab stops: Not at 3" + 6.5"

Formatted: Font: Not Italic

Formatted: Font: Bold, Not Italic

Formatted Table

Formatted: Font: 10 pt, Bold

Formatted: 06 Main Text Format, Centered

Formatted: Font: Bold

Formatted: Font: 10 pt, Bold

Formatted: 06 Main Text Format, Centered

Formatted: Font: Bold

Formatted: Font: 10 pt, Bold

Formatted: 06 Main Text Format, Centered

Formatted: Font: Bold

Formatted: Font: 10 pt, Bold

Formatted: 06 Main Text Format, Centered

Formatted: Font: Bold

Formatted: Font: 10 pt, Bold

Formatted: 06 Main Text Format, Centered

Formatted: Font: Bold

Formatted: Font: 10 pt, Bold

Formatted: 06 Main Text Format, Centered

Formatted: Font: Bold

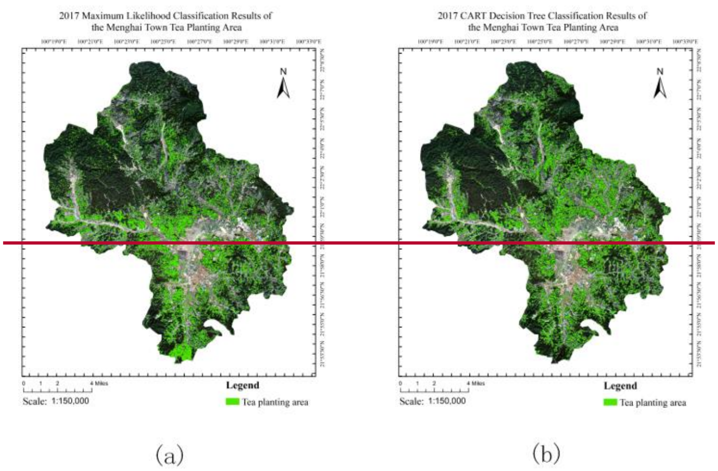

Fig~~ure~~ 9. ~~**Maximum likelihood**~~ **Tea extraction result.** The base map of the LandSat8 OLI images with the following composition:R (4),G (3) and B (2).The LandSat8 OLI images were downloaded from USGS National Map Viewer.~~**and CART decision tree**~~

Then, ~~this~~ proposed method was utilized to extract tea from three periods in 2014, 2015, and 2016, and the results contained tea-growing areas (*Fig.* 10). To examine the accuracy of the results, the traditional accuracy assessment method was used to generate a confusion matrix (*Table 6*). ~~According to the analysis of the confusion matrix, the accuracies of 2014 and 2015 were lower than the accuracy of 2016 because of the difficulty of sample selection. Field data are difficult to obtain, while tea area is difficult to identify by visual interpretation in GF-1 image data.~~ Some extracted tea areas were ~~located~~ in the deep forest, but most tea was normally planted at the edge of a forest and in the neighborhood of the residential area during field research. However, the result accuracy was satisfactory for applications.

Table ~~7~~6. Accuracy assessment of ~~2017~~ 2014-2016 SVM classification result

~~**time**~~	**2014**	**2015**	**2016**
**Overall accuracy**	~~84~~90.43%	~~86~~90.65%	~~92~~93.14%
**Kappa coefficient**	~~0.62~~0.71	~~0.59~~0.75	~~0.77~~0.81
**Producer accuracy (tea)**	~~64~~87.24%	~~87~~73.84%	~~83~~89%
**Producer accuracy (other vegetation)**	~~84~~91.16%	~~86~~97.31%	~~94~~94.5%
**User accuracy (tea)**	~~94~~69.23%	~~55~~91.6%	~~80~~84.17%
**User accuracy (other vegetation)**	96.6%	90.37	96.31%

Formatted: Font: 11 pt, Bold, English (United Kingdom)
Formatted: (Asian) Chinese (PRC), (Other) English (United Kingdom), Do not check spelling or grammar
Formatted: Font: Bold, (Asian) Chinese (PRC), (Other) English (United Kingdom), Do not check spelling or grammar
Formatted: Font: Bold, Not Italic
Formatted: Font: Not Bold
Formatted: Font: Bold, Not Italic
Formatted: Font: Not Italic
Formatted: Font: Bold, Not Italic
Formatted Table
Formatted: Font: 10 pt, Bold
Formatted: 06 Main Text Format, Centered
Formatted: Font: Bold
Formatted: Font: 10 pt, Bold
Formatted: 06 Main Text Format, Centered
Formatted: Font: Bold
Formatted: Font: 10 pt, Bold
Formatted: 06 Main Text Format, Centered
Formatted: Font: Bold
Formatted: Font: 10 pt, Bold
Formatted: 06 Main Text Format, Centered
Formatted: Font: Bold
Formatted: Font: 10 pt, Bold
Formatted: 06 Main Text Format, Centered
Formatted: Font: Bold
Formatted: Font: 10 pt, Bold
Formatted: 06 Main Text Format, Centered
Formatted: Font: Bold

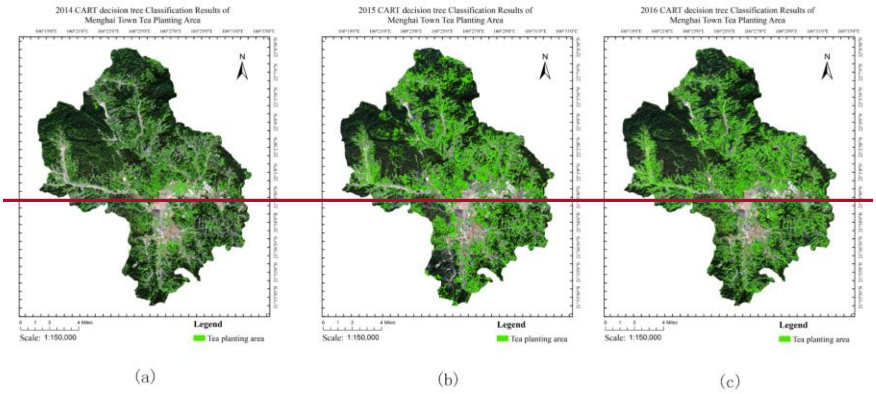

Fig~~ure~~ 10. Extraction results in (a)2014 (b)2015 and (c)2016. The base map of the LandSat8 OLI images with the following composition:R (4),G (3) and B (2).The LandSat8 OLI images were downloaded from USGS National Map Viewer.

Change detection

The ~~eCogntion~~ ENVI software was utilized for change detection, while the area was calculated by Arc ~~RC~~GIS software for statistical analysis. The spatial variation distribution (*Fig. 11*) and area change situation were analyzed (*Table 7*, *Fig. 12*, *13*). The overall pattern of spatial variation is expansion to the mid-west and east. The tea plantation area sharply increased between ~~2014~~ 2015 and ~~2015~~ 2016 and slowly increased between ~~2015~~ 2014 and ~~2016~~ 2015~~but slightly decreased from 2016 to 2017~~. The increasing rate was much slower between 2016 and 2017.

Table ~~8~~7. Tea plantation area changes during 2014-2017

T~~t~~ime	2014	2015	2016	2017
A~~a~~rea/acre	~~14953.65~~13757.64	~~18130.59~~14705.58	~~18006.4~~17765.30	~~18422.41~~17853.00

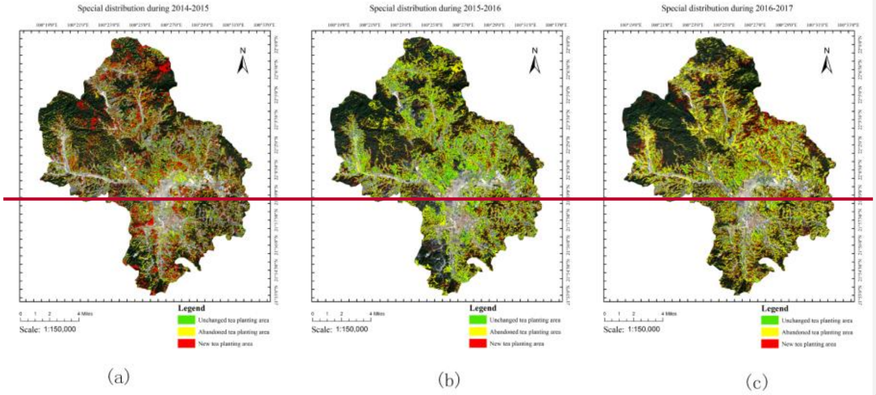

Fig~~ure~~ 11. Spatial distribution during (a)2014-2015 (b)2015-2016 and (c)2016-2017. The base map of the LandSat8 OLI images with the following composition:R (4),G (3) and B (2).The LandSat8 OLI images were downloaded from USGS National Map Viewer.

Formatted: Font: Not Italic

Formatted: Font: Not Bold

Formatted: 10 Figure Titles, Left, Indent: First line: 0 ch, Line spacing: single, Tab stops: Not at 3" + 6.5"

Formatted: Font: 16 pt, Not Italic

Formatted: Font: 16 pt, Bold, Font color: Black, Do not check spelling or grammar

Formatted: Font: Not Italic

Formatted: Font: Bold, Not Italic

Formatted Table

Formatted: Font: Bold

Formatted: (Asian) Chinese (PRC), (Other) English (United Kingdom), Do not check spelling or grammar

Formatted: Font: Bold, (Asian) Chinese (PRC), (Other) English (United Kingdom), Do not check spelling or grammar

Formatted: Font: Bold, Not Italic

Formatted: Font: Not Bold

Formatted: Font: 11 pt, Bold, English (United Kingdom)

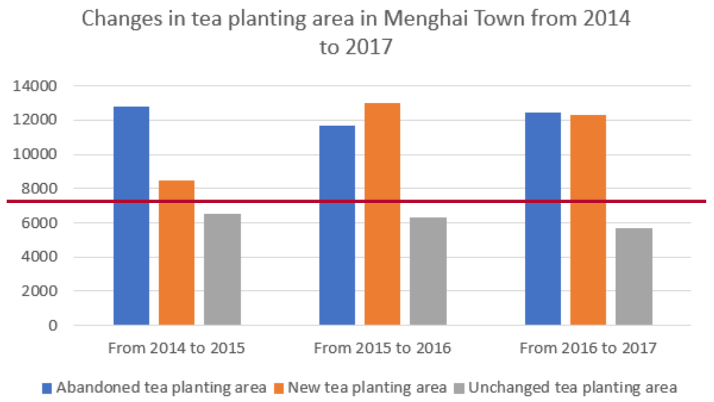

Fig~~ure~~ 12. Spatial variation from 2014 to 2017~~Area change from 2014 to 2017~~

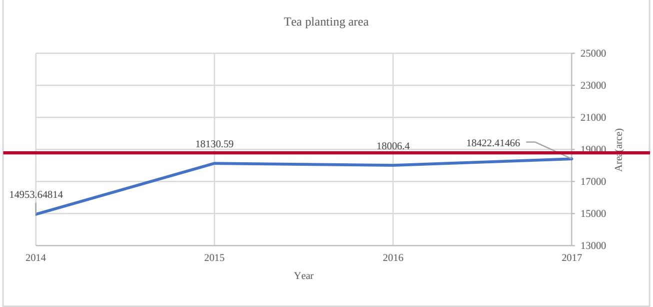

Fig~~ure~~ 13. Area change from 2014 to 2017~~Special variation from 2014 to 2017~~

Discussion

In this study, our primary objective was to analyze and propose a tea plantation mapping method and monitor the tea plantation area in Menghai Town ~~during~~ from 2014 to 2017. ~~The NDVI thershold segmentation metohd was used to extract vegtation area from GF-1 data.~~ The optimal segmentation parameters, such as segmentation scale (SS), weight of spectrum (WS1), weight of shape (WS2), weight of smoothness (WS3), and weight of compactness (WC), were selected by calculate and ana~~y~~lyze the local variance (LV) and the rate of change (ROC) of image homogeneity. The experiment results indicated that the optimal extraction of tea plantation are achieved when SS is set to 66, the WS is set to 0.5, and the WC is set to 0.7. The SS determines the size of the image object, which~~If~~ means ~~SS set~~ higher SS causing larger~~, the~~ image object and missing ~~is too large and~~ details~~ are missing~~. ~~This~~ We ~~finding~~ found ~~indicated~~ that for tea plantation mapping SS can not set too high because of the planting characteristics and vegetation form. WS1 is based on the standard deviation of the spectral colors, while WS2 is based on the deviation of a compact(or smooth) shape. WS1 determines to what degree spectrum influences the segmentation compared to shape, which against WS2. In Menghai town, most tea plantations have regular shape, so high WS2 can achieve better segmentation results. The value of WC gives it a relative weighting against WS3. The

Formatted: English (United States)

Formatted: (Asian) Chinese (PRC), (Other) English (United Kingdom), Do not check spelling or grammar

Formatted: Font: Bold, (Asian) Chinese (PRC), (Other) English (United Kingdom), Do not check spelling or grammar

Formatted: Font: Bold, Not Italic

Formatted: Font: 11 pt, Bold, English (United Kingdom)

Formatted: (Asian) Chinese (PRC), (Other) English (United Kingdom), Do not check spelling or grammar

Formatted: Font: Bold, (Asian) Chinese (PRC), (Other) English (United Kingdom), Do not check spelling or grammar

Formatted: Font: Bold, Not Italic

Formatted: Font: 11 pt, Bold, English (United Kingdom)

plantation form of tea in Menghai town is relatively compact, which make properly higher WC segment tea plantation better[21].

In order to build feature space for image objects to utilize the classification algorithm, SEaTH method is used to calculate the J-M distance of both spectrum~~l~~ and texture features between tea plantation and other vegetations. The experiment results showed that texture features, such as GLCM and GLDV, have the highest J-M distance~~, and most of them are higher than 1.5~~. Since tea plantation in this area have more regular plantation form which significantly different with other vegetation, texture features are more suitable to separate tea from other vegetation. ~~The highest one is GLCM in angle 135°, which is 1.80.~~ Besides,~~-~~ the mean value of the red band also have a relatively high J-M distance. Because vegetation coverage in tea plantation area normally lower than other vegetation in this area such as forest, the absorption effect of the red band in tea plantation area is relatively lower than other vegetation, causing high discrimination in the mean value of red band [22].

Then decorrelation method was used to remove the correlated features by calculating~~e~~ the correlation coefficients in order to build feature space without ~~-~~redundancy. The results indicated that although J-M distance of most texture features are high, the correlation coefficients are also high, which causes redundancy. Figure 14 shows the correlation coefficients of some texture features. ~~The results indicated that the mean of GLCM in angle 90°, area standard deviation, mean of red band and GLCM ASM have low corelation coefficients and relatively high J-M distance, which can be used to build features to utilize the classification methods.~~

Fig 14. Correlation coefficients of texture features

The accuracy of ~~The~~ maximum likelihood algorithm, CRAT algorithm~~ and~~, CNN-based method(Tang et al.,2020) and ~~maximum likelihood~~SVM algorithm ~~was~~ were ~~compared~~ evaluated at data in 2017 to find the most suitable algorithm~~, using the builded feature space~~. Besides, the performing of binary classification(tea and other vegetation) and multi-classification(tea, cropland and forest) was assessed. Table 9 shows the accuracy assessment results. ~~The experiments results showed that CRAT~~ SVM with multi-classification~~algorithm~~ achieve~~e~~ing ~~92~~93.14% overall accuracy ~~and~~ , 0.81 ~~k~~Kappa coefficient and 87.7% user accuracy outperforming~~ed~~ ~~maximum likelihood algorithm 4% in overall accuracy and 0.06 in Kappa coefficient~~other methods. The experiment results also indicates that multi-classification method achieving better accuracy than binary classification method in all methods. Multi-classification CART improves 4.15% of overall accuracy and 9.97% of user accuracy on binary classification, which higher than SVM, because SVM only uses support vector to build classifier, which are more robust than CART in binary classification. Besides, multi-classification CNN slightly improves the accuracy of tea extraction on binary classification CNN, which are 0.01 of kappa coefficient and 4.16% of producer accuracy of tea. ~~These findings clearly showed that CART algorithm is more sutiable for the tea plantation mapping, and the combination of special feature, spectral feature and texture feature can achieve promising results in tea plantation mapping.~~

Table 9. Accuracy assessment of 2017 binary classification and multi-classification result ~~Correlation coefficients of texture features~~

~~C~~	Maximum likelihood(binary)	Maximum likelihood(multi)	CART (binary)	CART (multi)	CNN (binary)	CNN (multi)	SVM (binary)	SVM (multi)

Formatted: (Asian) Chinese (PRC), (Other) English (United Kingdom), Do not check spelling or grammar

Formatted: Font: Bold, (Asian) Chinese (PRC), (Other) English (United Kingdom), Do not check spelling or grammar

Formatted: Font: Not Italic

Formatted: Font: Bold, Not Italic

Formatted: Font: Bold, Not Italic, English (United Kingdom)

Formatted: Font: Bold, Not Italic

Formatted Table

**Overall accuracy**	82.5%	86.15%	86.6%	90.75%	86.9%	86.7%	91.94%	93.14%
**Kappa coefficient**	0.60	0.67	0.66	0.76	0.64	0.65	0.77	0.81
**Producer accuracy (tea)**	93.35%	93.28%	86.60%	88.02%	72.5%	76.66%	79.87%	83.81%
**Producer accuracy (other vegetation)**	78.97%	83.83%	86.40%	91.63%	91.6%	90.01%	95.87%	96.16%
**User accuracy (tea)**	59.12%	65.28%	67.46%	77.43%	73.7%	71.43%	86.31%	87.7%
**User accuracy (other vegetation)**	97.32%	97.45%	95.18%	95.91%	91.1%	92.2%	93.6%	94.81%

Then, the best performing algorithm was used to mapping tea plantation at 2014 to 2017. Accuracy assessment was done by confusion matrix and kappa coefficient(*Table 6*). All overall accuracy were above ~~80~~90%, which proves the effectiveness of proposed method.

Conclusion

Menghai Town of Xishuangbanna Dai Autonomous Prefecture, Yunnan Province, Chian,was chosen as the study area, and GF-1 remotely sensed data from 2014 to ~~-~~2017 were chosen as the data source. Image texture, spectral and geometry features were integrated, while feature space was built by separability and thresholds algorithms (SEaTH) with decorrelation. Object-oriented image analysis (OOIA) with ~~classification and a regression tree (CART)~~SVM algorithm ~~were~~ was utilized to extract tea plantation areas. The salient findings of the study are as follows:

- OOIA can successfully suppress~~ed~~ noise by object-based classification and improve the effect of extraction.
- The selection of segmentation scale and parameters is a critical factor in OOIA. LV and ROC can be used as criteria to evaluate parameters in order to generate meanful image objects. ~~A s~~Scale parameter that is too small causes over segmentation, while a scale that is too large scale mixes different earth surface features in one image object.
- The feature ~~set~~ space built by the SEaTH algorithm and decorrelation with texture features can effectively avoid spectral confusion.
- OOIA combined with the ~~CART decision tree~~SVM algorithm and texture features solves the misclassification and missing-classification issues, yielding a Kappa coefficient up to 0.81 and an overall accuracy of ~~82~~93.14%, higher than ~~the maximum~~

Formatted: Font: Bold, (Asian) Chinese (PRC)

Formatted: Font: Bold, (Asian) Chinese (PRC)

Formatted: Font: Bold, (Asian) Chinese (PRC)

Formatted: Font: Bold, (Asian) Chinese (PRC)

Formatted: Font: Bold, (Asian) Chinese (PRC)

Formatted: Font: Bold, (Asian) Chinese (PRC)

Formatted: Font: 18 pt

~~likelihood~~CART algorithm~~method~~. Multi-classification method achieve better performance than binary classification in most algorithm.

- According to the extraction results, tea plantation areas are mainly distributed in the neighborhoods of residential areas with moderate slopes, ~~–~~because tea must be cultivated in easily drained areas and be convenient for farmers to reach. Based on variation analysis, we found that tea plantations expanded towards areas with sparse forests and moderate slopes.

Expectations

The extraction method of tea plantations based on RS still needs to be discovered. Currently, methods still have flaws in accuracy and efficiency. In consideration of this, we will focus on the following aspect in feature research:

- ESP is utilized to select the segmentation scale, but other parameters still need to be selected based on a visual assessment. The parameter selection method can be improved by automatically selecting the weight of the spectrum and the weight of compactness based on LV to achieve the optimal segmentation result.
- ~~According to~~In order to overcome ~~the~~ texture and spectral confusion, different data and extraction methods can be used: consider the growing period of tea based on low revisit period RS data; multi-source data fusion such as hyperspectral and high-resolution RS data to analyze the spectral and texture features; integrate with LiDAR data to obtain canopy height and shape information to reduce spectrum fixing; ~~integrate novel classification algorithms such as artificial intelligence and machine learning to improve accuracy~~use few sample learning method to overcome overfitting problem of deep learning and CNN.

Acknowledgements~~.~~

~~——This work was supported by Multi-government International Science and Technology Innovation Cooperation Key Project of National Key Research and Development Program of China , grant number 2018YFE0184300; the National Natural Science Foundation of P.R. China, grant number 41561048; the Program for Innovative Research Team (in Science and Technology) in the University of Yunnan Province, IRTSTYN; and the Undergraduate Research Innovation Foundation of Yunnan Normal University.~~ The authors are grateful to the editor and anonymous referees for their valuable comments and helpful suggestions. We wish to thank professor Cheng Wang and Xiaohuang Xi for their advice.

References~~References~~

1. Li Z, He LQ, Tea and Meteorology. 1st ed. Beijing, China: Meteorological Press; 2005. pp. 25-26.

Formatted: Font: 18 pt, Not Italic, (Asian) Chinese (PRC), (Other) Hungarian, Kern at 12 pt

Formatted: Font: 18 pt

Formatted: Font: 12 pt, (Asian) Chinese (PRC), (Other) Hungarian, Do not check spelling or grammar

Formatted: Indent: First line: 0.5"

Formatted: Font: 12 pt, (Asian) Chinese (PRC), Do not check spelling or grammar

Formatted: Font: 12 pt, (Asian) Chinese (PRC), Do not check spelling or grammar

Formatted: Font: 12 pt, (Asian) Chinese (PRC), Do not check spelling or grammar

Formatted: Font: 12 pt, (Asian) Chinese (PRC), (Other) Hungarian, Do not check spelling or grammar

Formatted: Font: 12 pt, (Asian) Chinese (PRC), Do not check spelling or grammar

Formatted: 04 Text of Abstract + Félkövér Char, Not All caps

2. Dihkan M, Guneroglu N, Karsli F. Remote sensing of tea plantations using an SVM classifier and pattern-based accuracy assessment technique. International Journal of Remote Sensing. 2013; 34(23): 8549-8565.
3. Xu WY, Sun R, Jin ZF. Extracting tea plantations based on ZY-3 satellite data. Transactions of the Chinese Society of Agricultural Engineering (Transactions of the CSAE). 2016; 32(Supp.1): 161-168.
4. Rajapakse RS, Tripathi NK, Honda K. Spectral characterization and LAI modelling for the tea (Camellia sinensis (L.) O. Kuntze) canopy. International Journal of Remote Sensing. 2002; 23(18), 3569-3577.
5. He L, Kong F, Shen Z. Artificial Neural Network Ensemble for Land Cover Classification, World Congress on Intelligent Control & Automation 2006.
6. Li, Z, Li, Z. Mapping the Spatial Distribution of Tea Plantations Using High-Spatiotemporal-Resolution Imagery in Northern Zhejiang. Forests. 2019; 10(10): 856.
7. Jun Z, Ziwu P, Hang W, Peijie H, Jiulin S, Fen Q, et al. An Improved Multi-temporal and Multi-feature Tea Plantation Identification Method Using Sentinel-2 Imagery. Sensors. 2019; 19(9): 2087.
8. Mallinis G, Koutsias N, Tsakiri-Strati M, et al. Object-based classification using Quickbird imagery for delineating forest vegetation polygons in a Mediterranean test site. ISPRS Journal of Photogrammetry & Remote Sensing. 2008; 63(2), 237-250.
9. Zixia T, Mengmeng L Xiaoqin W. Mapping Tea Plantations from VHR Images Using OBIA and Convolutional Neural Networks. Remote Sensing. 2020; 12(18): 2935.
10. Wei YH. Remote Sensing Digital Image Processing Tutorial, 2nd ed. Beijing, China: Science Press; 2015. pp. 185-304.
11. Sun JB. Principle and Application of Remote Sensing, 3rd ed. Wuhan, China: Wuhan University Press; 2013. pp. 108-224.
12. Deng SB, Chen QJ, Du HJ. ENVI Remote Sensing Image Processing Method, 2nd ed. Beijing, China: Beijing Higher Education Press; 2014. pp. 132-228,
13. Gonzalez RC, Woods RE, Eddins SL. Digital Image Processing Using MATLAB, 2nd ed.Beijing, China: Publishing House of Electronics Industry; 2009. pp. 135-136.
14. Otsu N. A Threshold Selection Method from Gray-Level Histograms. IEEE Transactions on Systems Man, and Cybernetics. 1979; 9(1), 62-66.
15. Liu JH. Mao ZY. Review of High Spatial Resolution Remote Sensing Image Segmentation Methods. Remote Sensing Information. 2009; 11(6): 95-101.
16. Drǎgu, L, Tiede D, Levick SR. ESP: a tool to estimate scale parameter for multiresolution image segmentation of remotely sensed data. International Journal of Geographical Information Science. 2010; 24(6): 859-871.
17. Zhang J. Classification and Feature Selection on High-dimensional and Small-sampling Data. 2014; Hefei University of Technology.
18. Dong LY, Xing LX, Pan J. Vegetation Classification in Hyper-spectral Image with CART Decision Tree. Journal of Jilin University Information Science Edition. 2013; 1: 83-89.
19. Jiang HW, Ren HQ, Qin XF. Auto Interpretation and Accuracy Evaluation of Images Based on eCognition. Bulletin of Surveying and Mapping. 2015; 10, 81-84.
20. Sven N, Gunter M. Object-Based Image Analysis and Treaty Verification. Springer Netherlands. 2008; 51-62.
21. Benz UC, Hofmann P, Willhauck G. Multi-Resolution, Object-Oriented Fuzzy Analysis of Remote Sensing Data for GIS-Ready Information. ISPRS Journal of Photogrammetry & Remote Sensing. 2004; 58: 239–258.
22. Henchiri M, Liu Q, Essifi B. Spatio-Temporal Patterns of Drought and Impact on Vegetation in North and West Africa Based on Multi-Satellite Data. Remote Sensing. 2020; 12(23): 3869.

~~Deng Shubin, Chen Qiujin, Du Huijian. ENVI Remote Sensing Image Processing Method, 2nd ed.; Beijing Higher Education Press: Beijing, China, 2014, 132-228,~~

Supporting Information

S1 File. 2014TeaExtractionResult. Deng Shubin, Chen Qiujin, Du Huijian. ENVI Remote Sensing Image Processing Method, 2nd ed.; Beijing Higher Education Press: Beijing, China, 2014, 132-228.

S2 File. 2015TeaExtractionResult.

S3 File. 2016TeaExtractionResult.

[1] **S4 File. 2017TeaExtractionResult.**Dihkan M, Guneroglu N, Karsli F. Remote sensing of tea plantations using an SVM classifier and pattern based accuracy assessment technique. International Journal of Remote Sensing 2013, 34(23), 8549-8565.

[2] Dong Lianying, Xing Lixin, Pan Jun. Vegetation Classification in Hyper-spectral Image with CART Decision Tree, Journal of Jilin University, Information Science Edition 2013 ,1, 83-89.

[3] Drǎgu, Lucian, Tiede D, Levick S R. ESP: a tool to estimate scale parameter for multiresolution image segmentation of remotely sensed data, International Journal of Geographical Information Science 2010, 24(6),859-871.

[4] Gonzalez R C, Woods R E, Eddins S L. Digital Image Processing Using MATLAB, 2nd ed.; Publishing House of Electronics Industry: Beijing, China, 2009, 135-136.

[5] He L, Kong F, Shen Z. Artificial Neural Network Ensemble for Land Cover Classification, World Congress on Intelligent Control & Automation 2006.

[6] Jiang Haowei, Ren Hongquan, Qin Xianfeng. Auto Interpretation and Accuracy Evaluation of Images Based on eCognition, Bulletin of Surveying and Mapping 2015 ,10, 81-84.

[7] Jun Zhu, Ziwu Pan, Hang Wang, Peijie Huang, Jiulin Sun, Fen Qin, Zhenzhen Liu. An Improved Multi-temporal and Multi-feature Tea Plantation Identification Method Using Sentinel-2 Imagery, Sensors 2019, 19(9), 2087.

[8] Li Zhuo, He Lingqi, Tea and Meteorology, 1st ed.; Meteorological Press: Beijing, China, 2005; 25-26.

[9] Li, Zhang, Li, Zhang. Mapping the Spatial Distribution of Tea Plantations Using High-Spatiotemporal-Resolution Imagery in Northern Zhejiang 2019, Forests 10:10, 856.

[10] Liu Jianhua. MAO Zheng-yuan. Review of High Spatial Resolution Remote Sensing Image Segmentation Methods, Remote Sensing Information 2009, 95-101.

[11] Liu Shuhan, Gu Xingfa, Yu Tao. Object-Oriented Classification method based on GF-1 Multi-spectral Remote Sensing image, Surveying Science 2014,12, 91-103.

[12] Mallinis G, Koutsias N, Tsakiri-Strati M, et al. Object-based classification using Quickbird imagery for delineating forest vegetation polygons in a Mediterranean test site, ISPRS Journal of Photogrammetry & Remote Sensing 2008, 63(2), 237-250.

[13] Otsu N. A Threshold Selection Method from Gray-Level Histograms, IEEE Transactions on Systems, Man, and Cybernetics 1979, 9(1), 62-66.

[14] Rajapakse R M S S, Tripathi N K, Honda K. Spectral characterization and LAI modelling for the tea (Camellia sinensis (L.) O. Kuntze) canopy, International Journal of Remote Sensing 2002, 23(18), 3569-3577

[15] Sun Jiabing. Principle and Application of Remote Sensing, 3rd ed.; Wuhan University Press: Wuhan, China, 2013, 108-224.

[16] Sven Nussbaum, Gunter Menz. Object-Based Image Analysis and Treaty Verification, Springer Netherlands 2008,51-62.

[17] Wei Yuchun. Remote Sensing Digital Image Processing Tutorial, 2nd ed.; Science Press: Beijing, China, 2015, 185-304.

Formatted: No bullets or numbering

Formatted: No bullets or numbering

Formatted: No bullets or numbering

~~[18] Xu Weiyan, Sun Rui, Jin Zhifeng. Extracting tea plantations based on ZY-3 satellite data, Transactions of the Chinese Society of Agricultural Engineering (Transactions of the CSAE) 2016, 32(Supp.1), 161-168.~~

~~[19] Zhang Jing. Classification and Feature Selection on High-dimensional and Small-sampling Data 2014, Hefei University of Technology.~~

~~[20] Zixia Tang, Mengmeng Li, Xiaoqin Wang. Mapping Tea Plantations from VHR Images Using OBIA and Convolutional Neural Networks. Remote Sensing 2020, 12:18, pages 2935.~~

Revised Manuscript with Track Changes

Mapping Pu'er tea plantations from GF-1 images using Object-Oriented Image Analysis (OOIA) and Support Vector Machine (SVM)

LIANG, L.[1,2,3]– WANG, J. L.[1,2,3,*] –DENG, F.[1,2,3]–KONG, D.Y.[1,2,3]

1Faculty of Geography, Yunnan Normal University, Kunming 650500, China
2Key Laboratory of Resources and Environmental Remote Sensing for Universities in Yunnan, Kunming 650500, China
3Remote Sensing Research Laboratory, Center for Geospatial Information Engineering and Technology of Yunnan Province, Kunming 650500, China
* Corresponding author
E-mail: jlwang@ynnu.edu.cn

Style Definition: 05 Main Section Title: Font: 18 pt

Style Definition: 09 References Title: Font: 18 pt, Left

Style Definition: 10 Figure Titles: Font: Not Italic, Indent: First line: 0.15"

Mapping Pu'er tea plantations from GF-1 images using Object-Oriented Image Analysis (OOIA) and Support Vector Machine (SVM)

~~Mapping Pu'er Tea Plantations From Gf-1 Images Using Ooia And CART~~

LIANG, L.[1,2,3] – WANG, J. L.[1,2,3,]* –DENG, F.[1,2,3] –KONG, D.Y.[1,2,3]

[1] *Faculty of Geography, Yunnan Normal University, Kunming 650500, China*

[2] *Key Laboratory of Resources and Environmental Remote Sensing for Universities in Yunnan, Kunming 650500, China*

[3] *Remote Sensing Research Laboratory, Center for Geospatial Information Engineering and Technology of Yunnan Province, Kunming 650500, China*

** Corresponding author*
e-mail: jlwang@ynnu.edu.cn

Abstract~~.~~

Tea is~~-~~ the most popular drink worldwide, and China is the largest producer of tea. Therefore, tea is an important commercial crop in China, playing a significant role in domestic and foreign markets. It is necessary to make accurate and timely maps of the distribution of tea plantation areas~~ to~~ for plantation management and decision making. In the present study, the town of Menghai in the Xishuangbanna Dai Autonomous Prefecture, Yunnan Province, China, was chosen as the study area, and GF-1 remotely sensed data from 2014-2017 were chosen as the data source. Image texture, spectral and geometrical ~~geometry~~ features were integrated, while feature space was built by SEparability and THresholds algorithms~~separability and thresholds algorithms~~ (SEaTH) with decorrelation. Object-Oriented Image Analysis~~Object-oriented image analysis~~ (OOIA) with a Support Vector Machine ~~classification and regression tree~~ (~~CART~~SVM) algorithm was utilized to map tea~~-~~ plantation areas. The overall accu~~-~~racy and Kappa coefficient of the proposed method were 9~~2.00~~3.14% and 0.81, respectively, ~~4~~3.61% and 0.05~~.~~, 6.99~~14~~% and 0.1~~54~~, 6.44% and 0.16 better than the results of ~~the maximum likelihood~~CART method, ~~and~~ Maximum likelihood method and CNN based method. The tea plantation area increased by 4,095.36 acre~~14 km2~~ from 2014 to 2017, while the fastest-growing period is 2015 to 2016~~, with slight fluctuations during this period~~.

Keywords: *Pu'er tea, OOIA, Feature selection, ~~Decision tree~~SVM, Change detection*

Introduction

Tea is the most popular drink worldwide~~ (Li et al., 2005)~~[1], and the plantation and production of tea play~~s~~ an important role in agricultural economies and country development. Furthermore, the excessive plantation of tea has many negative impacts on environ~~-~~ments such as landslides and water and soil loss~~ (Dihkan et al.,2013)~~[2]; therefore, the continuous moni~~-~~toring of tea-growing areas has significant importance.

Traditionally, related departments use ~~manual~~ visual interpretation methods to monitor tea distribu~~-~~tion from remote sensing image, but these methods are inefficient, have a low

Formatted: Font: 18 pt

Formatted: Font: 12 pt, (Asian) Chinese (PRC), Do not check spelling or grammar

Formatted: Indent: First line: 0.5"

Formatted: Font: 12 pt, (Asian) Chinese (PRC), Do not check spelling or grammar, Not Superscript/ Subscript

Formatted: Font: 12 pt, (Asian) Chinese (PRC), Do not check spelling or grammar

Formatted: Font: 12 pt, (Asian) Chinese (PRC), (Other) English (United Kingdom), Do not check spelling or grammar

precision, are expensive, and are challenged by the need for spatially continuous monitoring over large areas.

Remote sensing technology can obtain spatially continuous information on earth surfaces[3] ~~(Xu et al.,2016)~~, which has plenty of applications in tea spatial distribution extraction and monitoring, because this technology has wide data coverage, fast data-update speeds, and low costs.

However, different tea growing areas have spectral differences due to canopy structure and traits, plantation size, crop health and growing period, enlarging the within-class distance of tea in spectral features. The spectral reflectance of tea is close to that of other vegetation, such as fruit trees and bushes, narrowing the class distance. Therefore, the spectral mixing phenomenon makes it extremely difficult to extract tea plantation distributions ~~(Rajapakse et al.,2002)~~[4]. For example, He[5] used an artificial neural network (ANN) to classify vegetation using Landsat Thematic Mapper (TM) images found that the extraction of bushes and tea had a relatively low accuracy, but the extraction of other vegetation achieved a high accuracy. One effective method of plantation distribution extraction is to use spatial information based on texture features. For instance, Xu[3] built a decision tree based on the texture features of anisotropic strength to extract the tea plantation distribution in mountain areas, using ZY-3 satellite data. Dihkan[2] extracted the Gabor texture feature from multispectral airborne digital image to train a support vector machine classifier (SVM) and extract tea plantations distribution in black sea areas, achieving an overall accuracy above 90%.~~For example, He (He et al.,2006) used an artificial neural network (ANN) to classify vegetation and found that the extraction of bushes and tea had a relatively low accuracy, but the extraction of other vegetation achieved a high accu-racy. One effective method of plantation distribution extraction is to use spatial in-formation based on texture features. For instance, Xu (Xu et al.,2016) built a decision tree based on the texture features of anisotropic strength to extract the tea plantation distribution in mountain areas. Dihkan (Dihkan et al.,2013) uses the Gabor texture feature to train a support vector machine classifier (SVM) and extract tea plantations distribution in black sea areas, achieving an overall accuracy above 90%.~~ Another method is using time series analysis to extract temporal feature based on regular annual growth cycle of tea. For example, Li ~~(Li et al.,2019)~~[6] used high-spatiotemporal-resolution VENμS images to extract temporal and spectral features, ~~-~~and utilized decision tree to mapping tea plantation. Zhu ~~(Zhu et al.,2019)~~[7] inte~~-~~grates multi-temporal features to train a random forest classifier based on middle spital resolution satellite remote sensing data~~-~~ Sentinel-2. However, high-temporal-resolution means either low spatial resolution such as MODIS or small coverage such as VENμS, which not able to map ~~large~~ extensive area. ~~Due to~~Because of data limitations, most previous studies based on mid/low-resolution satellite remote sensing data were unable to effectively extract texture features in tea-growing areas, while few high spatial resolution~~high-resolution~~-based studies used aerial remote sensing data, with high costs and a relatively small coverage.

High spatial resolution~~High-resolution~~ satellite remote sensing data contain sufficient targets details, advancing the effect of the inside texture and structure features of targets. However, too exhaustive details may cause salt-and-pepper noise in information extraction and image classification. OOIA solves this problem effectively. For instance, Mallinis ~~(Mallinis et al.,2008)~~ [8] used OOIA to extract the distribution of vegetation based on Quickbird satellite remote sensing data, which had a spatial resolution of 0.61 m and improved the classification accuracy. Tang ~~(Tang et al.,2020)~~[9] integrated OOIA

with convolutional neural network to mapping tea plantation, achieving comparable results and can be used with limited training samples.

Menghai town in the Xishuangbanna Dai Autonomous Prefecture, Yunnan Prov~~-~~ince, was chosen as the study area, and GF-1 remotely sensed data from 2014 to ~~-~~2017 were chosen as the data source. In this study, OOIA and texture features were com~~-~~bined, while features selected by SEaTH, the CART algorithm and ~~a maximum likeli-hood~~SVM algorithm were compared to find the best way to extract the tea plantation area and analyze the plantation distribution and changes. In that case, the change trends of tea plantations can be analyzed to assist relevant departments in decision making.

Study area and data source

Study area

Menhai county is ~~located~~ in the southwestern part of Yunnan province, the southwest corner of China. The longitude of this county is between 99°56′E～100°41′E, and the latitude is between 21°28′N～22°28′N (Fig. 1). The study area has a tropical monsoon climate, with 1136-1513 ml precipitation per year. The annual average tem~~-~~perature is approximately 18.9℃ - 22.6℃, which is extremely suitable for tea growth, making this area one of the original plantations of "Pu Er tea". As of 2017, the overall tea plantation area was approximately 710 thousand acres, making the tea industry the largest and most profitable industry in Menghai County. Menghai town is ~~located~~ in eastern Menghai County, which is the political, economy, and cultural center of Menghai County. The area of this town is 365.38 $\boldsymbol{km^2}$, ~~，~~ of which 45.3 $\boldsymbol{km^2}$~~-~~, is tea plan~~-~~tation area, which show~~make~~s the tea industry plays a serious role in the local economy.

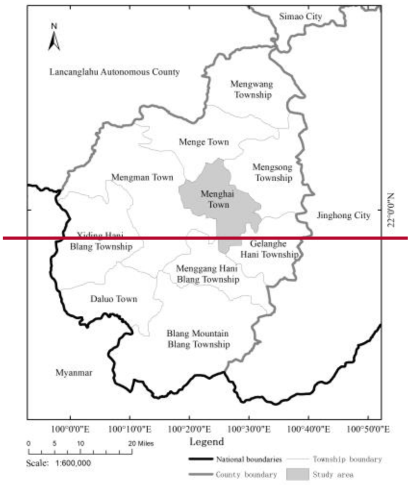

Fig~~ure~~ 1. Study area

Data source and preprocessing

Formatted: Font: 16 pt, Not Italic

Formatted: Font: Not Italic

Formatted: Font: Bold

Formatted: Font: 16 pt, Not Italic

The PMS(Panchromatic and multispectral sensors) data products from 2014 to -2017, which were obtained from the GF-1 satellite data, were used in the present study (Table 1). The preprocesses were supported by ENVI 5.3 software and ~~comprised~~ consisting of seven steps. Primarily, radiometric calibration~~radiation collaboration~~, FLAASH~~flash~~ atmosphere correction, orthorectification~~orthographical correction~~, image pan sharping and image mosaicking were completed. Then, 2017 data were selected as the base image to register the other three images by im-age-to-image registering ~~(Wei et al.,2015, Sun et al.,2013, Deng et al.,2014, Deng et al.,2014)~~[10-13]. The RMS of the image registering result was con-trolled below 1 pixel. Finally, vector data of Menghai town were used to mask the image to obtain four scene images of Menghai town.

Table 1. GF-1 ~~SATELLITE~~ satellite PMS ~~DATA~~ data ~~PARAMETERS~~parameters

Parameters	2 m pan/8 m multiresolution ~~Parameters~~	
**Spectral range**	Pan	0.45-0.90 μm
	Multispectral	0.45-0.52 μm
		0.52-0.59 μm
		0.63-0.69 μm
		0.77-0.89 μm
**Spatial resolution**	Pan	2 m
	Multispectral	8 m
**Swath**	60 km (2 camera)	
**Temporal (sway)**	4 day	
**Temporal (not sway)**	41day	

Methods

Research workflow

A six-step method was used to analyze the tea plantations. First, the normalized difference vegetation index (NDVI) was calculated from the preprocessed image. Second, OTSU algorithm was used to select best NDVI threshold to separate non-vegetation area from vegetation area. The ~~T~~third step were selection of optimal segmentation and~~as~~ multiresolution segmentation~~, but the optimal parameters first had to be obtained~~. The fourth step was building feature space by the selection of best features using SEaTH. The fifth step was a comparison between the ~~performance~~ performing of SVM method, ~~the maximum likelihood algorithm and~~ CART method, Maximum likelihood method and CNN based method~~the CART decision tree~~ to determine the best method. Finally, the best-performing method was utilized to extract tea distribution information from the rest of the image and analyze the changing situation (*Fig. 2*).

Formatted: Font: Not Italic

Formatted: Normal, Indent: First line: 0", Space Before: 12 pt, After: 6 pt

Formatted: Font: 11 pt, Bold, (Asian) Chinese (PRC), (Other) English (United Kingdom), Do not check spelling or grammar

Formatted Table

Formatted: Font: Bold, (Asian) Chinese (PRC), (Other) English (United Kingdom), Do not check spelling or grammar

Formatted: Font: Bold, (Asian) Chinese (PRC), (Other) English (United Kingdom), Do not check spelling or grammar

Formatted: Font: Bold, (Asian) Chinese (PRC), (Other) English (United Kingdom), Do not check spelling or grammar

Formatted: Font: Bold, (Asian) Chinese (PRC), (Other) English (United Kingdom), Do not check spelling or grammar

Formatted: Font: Bold, (Asian) Chinese (PRC), (Other) English (United Kingdom), Do not check spelling or grammar

Formatted: Font: Bold, (Asian) Chinese (PRC), (Other) English (United Kingdom), Do not check spelling or grammar

Formatted: Font: Bold, (Asian) Chinese (PRC), (Other) English (United Kingdom), Do not check spelling or grammar

Formatted: Font: Bold, (Asian) Chinese (PRC), (Other) English (United Kingdom), Do not check spelling or grammar

Formatted: Font: Bold, (Asian) Chinese (PRC), (Other) English (United Kingdom), Do not check spelling or grammar

Formatted: Font: Bold, (Asian) Chinese (PRC), (Other) English (United Kingdom), Do not check spelling or grammar

Formatted: Font: 16 pt, Not Italic

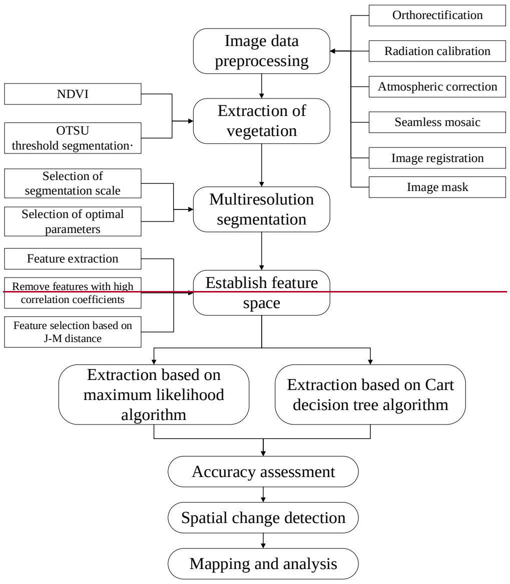

Fig~~ure~~ 2. Research workflow

Extraction of vegetation

The NDVI is a well-performing index to indicate vegetation conditions on the surface. This index is obtained by the spectral reflectance of available light and near infrared between 400 and ~~700~~ 860 nm because vegetation has special reflectance and absorption characteristics in th~~ei~~se bands. The NDVI is calculated as follows:

$$NDVI = \frac{NIR-R}{NIR+R} \quad (~~Eq.~~1)$$

where NIR is the spectral reflectance of near infrared channel near 860µm, while R is the spectral reflectance of red channel near 660µm.

Because vegetation areas contain tea plantation areas, vegetation areas can be easily distinguished from non-vegetation areas by the NDVI threshold. In that case, the selection of the best-performing threshold is the most crucial stage.

The OTSU algorithm was proposed by Otsu ~~(Otsu et al.,1979)~~[14], a Japanese researcher, in 1979, for image binarization, which was widely used to select the best threshold for grayscale segmentation in digital image processing. In this algorithm, the image is split~~ed~~ into two classes, frontward and backward, by selecting the histogram value for segmentation that makes the variance highest between the two classes. In other words, this algorithm calculates the lowest point between two classifications in the histogram.

NDVI was calculated from preprocessed image data from 2014 to 2017 to select the optimized threshold (*Table 2*). Figure 3 shows the histogram of NDVI in 2017. The histogram can be considered ~~to~~ havinge two peak forms, which makes the OTSU algorithm suitable. Figure 4 shows the results of the vegetation extraction for 2017, which achieved a quite accurate performance.

Table 2. Optimal thresholds in 2014-2017

Formatted: Font: 16 pt, Font color: Black, English (United States)

Formatted: 06 Main Text Format

Formatted: Font: 16 pt

Formatted: Normal, Indent: First line: 0", Space Before: 12 pt, After: 6 pt

Formatted: Font: 11 pt, Bold, (Asian) Chinese (PRC), (Other) English (United Kingdom), Do not check spelling or grammar

year	2014	2015	2016	2017
threshold	0.44	0.42	0.53	0.43

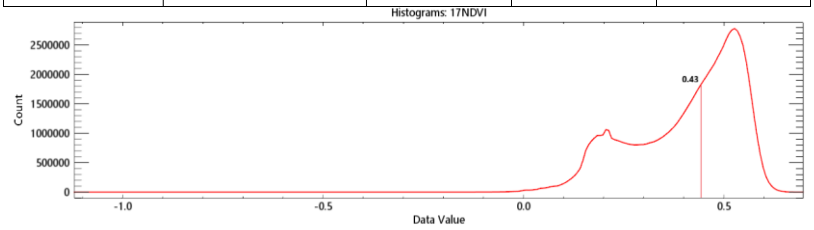

Figure 3. NDVI histogram in 2017

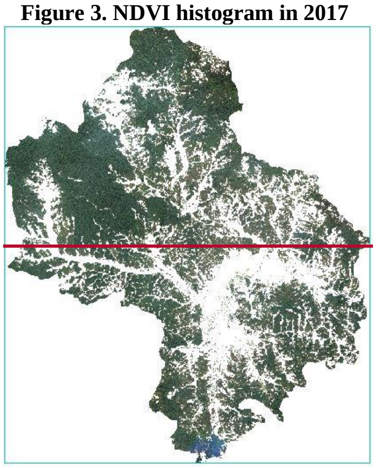

Figure 4. 2017 Vvegetation extraction results. The base map of the LandSat8 OLI images with the following composition:R (4),G (3) and B (2).The LandSat8 OLI images were downloaded from USGS National Map Viewer.

Selection of optimal parameters

To take advantage of the high-resolution feature and neighbor relationship of GF-1 satellite data and prevent noise, a multiresolution segmentation method, which combines neighbor pixels based on spectrum and shape to create image objects, was utilized(Liu et al.,2009)[15]. In this stage, the selection of segmentation scale (SS), weight of spectrum (WS1), weight of shape (WS2), weight of smoothness (WS3), and weight of compactness (WC) controlled the performance of segmentation.

The estimation of scale parameters (ESP) is an optimal parameter selection method based on local variance (LV) and the rate of change (ROC) of image homogeneity(Drăgu et al.,2010)[16]. LV is the average of the neighborhood standard deviation in the entire image. Along with the increase in SS, the inner homogeneity of the image object increased, and heterogeneity with other image objects decreased, causing the LV to rise and a slower change rate. When the optimal scale was achieved, the image objects had the highest similarity with earth surface features in reality, LV peaked, and ROC increased gradually. After visual assessment, the size and shape of the results were the most similar to the tea plantation area.

Formatted: Font: Bold

Formatted Table

Formatted: Font: Not Bold

Formatted: Font: 16 pt, Font color: Black, English (United States)

Formatted: 06 Main Text Format

Formatted: Font: 16 pt

In addition to the SS, the WS1 and WS2 control the segmentation results. WS2 also considers WS3 and WC. WS3 and WC are used to control the smoothness of the image object border and separation.

Based on the idea that the control variable is optimal, all SS and other parameters were traversed to integrate ESP and visual assessment to select optimal parameters. The results indicate that the optimal SS was 66, the optimal WS was 0.5, and the optimal WC was 0.7 (*Fig. 5*).

When a larger SS than the optimal SS was used (*Fig. 6*), e.g., 206, the area was too large, and details were missing, which can cause low accuracy in the classification step. When the SS was overlarge, the ROC of LV performing violent fluctuated, which is not the optimal parameter.

When a lower WC was used (*Fig. 7*), the shape of the image object was too regular, and the border was too smooth, which was not the same as reality (*Fig. 7 (b, c)*) and had a negative impact on segmentation. The optimal parameters resulted in the most complete extraction of tea plantation information, with distinct borders.

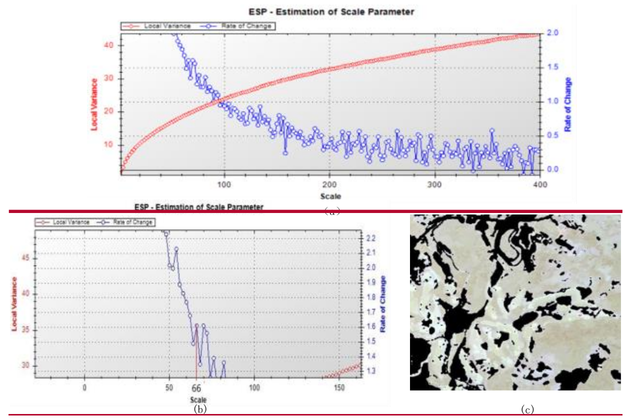

Figure 5. Multiresolution segmentation (SS=66, WS_1=0.5, WC=0.7): (a) variance curve, (b) optimal segmentation scale and (c) image object

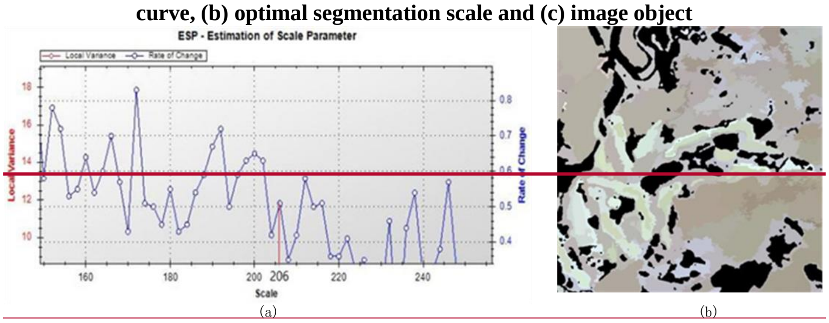

Figure 6. Multiresolution segmentation (SS=206, WS_1=0.5, WC=0.7): (a) variance curve and (b) image object

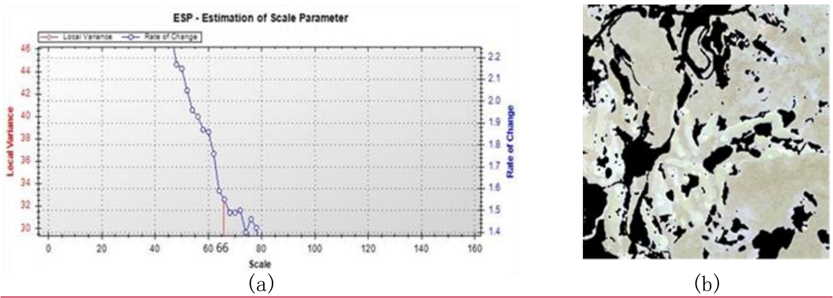

(a) (b)

Figure 7. Multiresolution segmentation (SS=66, WS_1=0.5, WC=0.1): (a) variance curve and (b) image object

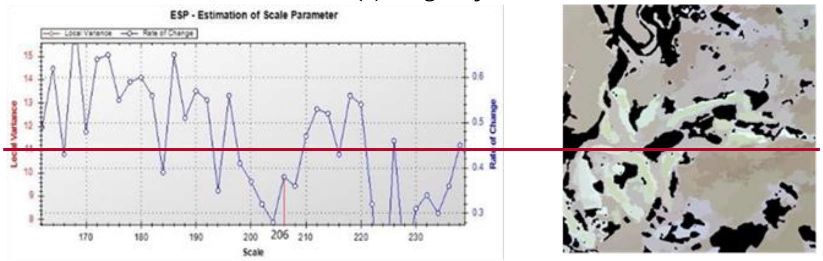

(a) (b)

Figure 8. Multiresolution segmentation (SS=206, WS_1=0.5, WC=0.1): (a) variance curve and (b) image object

Feature space establishment

The eCognition software offers hundreds of features, including spectrum, shape, texture, context, and spatial relationship features. The multidimensional feature space can solve the phenomenon caused by one-dimensional spectrum extraction. However, too many features can cause dimensional disasters, which damage calculation efficiency and accuracy~~(Zhang et al.,2014)~~[17]. Therefore, high efficiency and accuracy are crucial factors for the best selection from abundant features that have high discrimination and low redundancy.

The SEaTH algorithm is a feature selection method based on Jeffries Matusita(J-M) distance~~(Zhang et al.,2018)~~[17], which is calculated by the statistic index of two classes, such as the mean and variance, and used to measure discrimination in one feature dimensionality. A higher J-M distance value indicates better discrimination from the two ~~-~~class sample in one feature dimensionality. The J-M distance equation is:

$$J = 2(1 - e^{-B}) \qquad (\text{~~Eq.~~}2)$$

$$B = \frac{1}{8(m_1 - m_2)^2} * \frac{2}{\sigma_1^2 + \sigma_2^2} + \frac{1}{2} ln \frac{\sigma_1^2 + \sigma_2^2}{2\sigma_1 \sigma_2} \qquad (\text{~~Eq.~~}3)$$

where m_i and σ_i^2 (i = 1 , 2) refer to the mean and variance of a two-classification sample.

The range of the J-M distance is (0-2); 0 means completely mixed, while 2 means completely separated. Because the J-M distance is unable to completely reach 2, the best result is associated with the highest J-M distance.

Formatted: Font: 16 pt, Not Italic, English (United States)

Formatted: 06 Main Text Format, Line spacing: single

Formatted: Font: 16 pt, Not Italic

Formatted: Normal, Indent: First line: 1 ch, Line spacing: Multiple 1.05 li

Since multi-class classification often achieves better than binary classification. We split non-tea class by forest and cropland, and calculate J-M distance between tea and both of them. Table 3 shows the J-M distance results calculated from different features, with results sorted by value.

Table 3 J-M Distances of part features

	J-M distance（tea and corpland）	J-M distance（tea and forest）
Red band mean	0.75	1.05
GLCM Homogeneity all dir	0.44	0.84
GLCM Homogeneity 135	0.35	0.68
Avrage. area represented by segments	0.20	0.72
Stddev of area represented by segments	0.53	0.68
GLCM Homogeneity 45	0.48	0.59
Max. diff.	0.20	0.75

Feature name	J-M distance
GLCM_Ang_135°	1.80
GLCM_Ang_90°	1.64
GLCM_Ang_45°	1.63
GLCM_Ang_180°	1.62
GLDV_Ang_135°	1.20
GLDV_Ang_90°	1.13
GLDV_Ang_45°	1.05
GLCM_Hom	0.74
Mean_Layer_red	0.68
GLCM_Mean	0.66

Most features with high J-M distances are different operators based on the Gray-level cooccurrence matrix (GLCM) and Gray-level difference vector (GLDV). These features have strong discrimination but high correlation, which causes high redundancy and low accuracy. Thus, features with strong correlation coefficients should be removed. Pearson's correlation coefficient was utilized to calculate the correlation coefficients, as shown in Eq.4 and 5:

$$r_{ij} = \frac{\sum_{l=1}^{K}\left(x_i^l - \overline{x_i}\right)\left(x_j^l - \overline{x_j}\right)}{\sqrt{\sum_{l=1}^{K}\left(x_i^l - \overline{x_i}\right)^2 \sum_{l=1}^{K}\left(x_j^l - \overline{x_j}\right)^2}} \qquad \text{(Eq.4)}$$

$$\overline{x_i} = \frac{1}{K}\sum_{l=1}^{K} x_i^l \qquad \text{(Eq.5)}$$

where x_i^l refers to the i-th feature of the l-th sample and $\overline{x_i}$ refers to the mean value of the i-th feature.

Features with high correlation coefficients and relatively low J-M distances were removed from the 20 features with the highest J-M distances. The remaining features established a feature set with four ten features., i.e., two texture features, one spectral feature and one geometry feature (*Table 4*).

In this feature set, the most contributory features was were texture features such as GLCM Angular Second Moment (ASM), GLCM homogeneity, GLCM dissimilarity., which was a function of pixel distance and angle. The texture feature is reflected by the

correlation between two pixels at a certain distance and angle. Those features were calculated inside image object, using all pixels inside the image object.The equations of ~~GLCM Mean and GLCM ASM~~used texture features ~~equations~~ are shown (Eq.6~~ and Eq.7~~10).

$$GLCM_{Homogeneity} = \sum_{i,j=0}^{N-1} P_{i,j}/(1+(i-j)^2) \quad (6)$$

$$GLCM_{ASM} = \sum_{i,j=0}^{N-1} P_{i,j}^{2} \quad (7)$$

$$GLCM_{Dissimilarity} = \sum_{i,j=0}^{N-1} P_{i,j}|i-j| \quad (8)$$

$$GLCM_{StdDev} = \sqrt{\sum_{i,j=0}^{N-1} P_{i,j}(i,j-u_{i,j})} \quad (9)$$

$$GLDV_{Mean} = \sum_{i,j=0}^{N-1} k(V_k) \quad (10)$$

Because tea has similar spectral characteristics as other vegetation, the use of texture features can highly improve the accuracy and results of tea area extraction. Besides, the geometry features also have strong discrimination. The width of an image object is calculated using the length-to-width ratio. The Stddev of Area Represented by Segments is based on the standard deviation of triangles created by Delaunay triangulation. The number of neighbors calculated by the number of the direct neighbors of an image object. Finally, spectral features such as red band mean and max difference were used to build feature set. The Table 4 shows the best separate achievements in texture features.

Table 4 Correlation coefficients and J-M distances of feature set

	GLCM homogeneity	Width	GLDV mean	Max. diff	GLCM dissimilarity	GLCM StdDev	Number of neighbors	Stddev of area represented by segments	Red band mean	GLCM ASM
J-M distance (tea and forest)	0.85	0.04	0.21	0.76	0.21	0.09	0.01	0.68	1.06	0.16
J-M distance (tea and corpland)	0.44	0.86	0.76	0.20	0.76	0.78	0.91	0.54	0.75	0.89
GLCM homogeneity	1.00	0.50	-0.71	0.03	-0.71	-0.64	0.34	0.65	-0.01	-0.42
Width	0.50	1.00	-0.63	0.40	-0.63	-0.72	0.81	0.53	-0.49	-0.34
GLDV mean	-0.71	-0.63	1.00	-0.43	1.00	0.84	-0.55	-0.49	0.35	0.72
Max. diff	0.03	0.40	-0.43	1.00	-0.43	-0.59	0.52	0.00	-0.74	-0.30
GLCM dissimilarity	-0.71	-0.63	1.00	-0.43	1.00	0.84	-0.55	-0.49	0.35	0.72
GLCM StdDev	-0.64	-0.72	0.84	-0.59	0.84	1.00	-0.67	-0.50	0.53	0.53

Number of neighbors	0.34	0.81	-0.55	0.52	-0.55	-0.67	1.00	0.34	-0.58	-0.31
Stddev of area represented by segments	0.65	0.53	-0.49	0.00	-0.49	-0.50	0.34	1.00	-0.04	-0.23
Red band mean	-0.01	-0.49	0.35	-0.74	0.35	0.53	-0.58	-0.04	1.00	0.19
GLCM ASM	-0.42	-0.34	0.72	-0.30	0.72	0.53	-0.31	-0.23	0.19	1.00

	~~GLCM 90°mean~~	~~Area standard deviation~~	~~Red band mean~~	~~GLCM ASM~~
~~J-M distance~~	~~0.66~~	~~0.65~~	~~0.68~~	~~1.80~~
~~GLCM 90°mean~~	~~1~~	~~0.09~~	~~-0.44~~	~~-0.85~~
~~Area standard deviation~~	~~0.09~~	~~1~~	~~0.15~~	~~-0.08~~
~~Red band mean~~	~~-0.44~~	~~0.15~~	~~1~~	~~0.15~~
~~GLCM ASM~~	~~-0.85~~	~~-0.08~~	~~0.15~~	~~1~~

Classification method and accuracy assessment

~~The~~ Maximum likelihood algorithm, OOIA-based CART decision tree algorithm, CNN-based algorithm~~(Tang et al.,2020)~~[9] and ~~maximum likelihood~~SVM algorithm were compared using the feature set established by the above methods to extract tea from 2017 image data. In case of CNN-based algorithm, features was obtain by CNN and selected by Gini index, and classifer was built by random forest, following the method proposed by Tang~~(Tang et al.,2020)~~[9]. ~~-~~In case of SVM algorithm, the Radial Basis Function (RBF) was used as kernel function in order to deal with linear inseparable case.

The samples were gathered by field research and visual interpretation. The number and construction of samples were shown in Table 5. We split them ~~by 80/20~~ for training and ~~validation~~testing. So, ~~T~~the confusion matrix was calculated based on ~~929~~ 2108 non-tea samples and ~~255~~ 303 tea samples obtained by field observation to assess accuracy~~(Dong et al.,2013, Jiang et al.,2015)~~[18-20]. The best algorithm was chosen by comparing the overall accuracy, k~~K~~appa coefficients, producer accuracy, and user accuracy to achieve the best performance during the extraction of tea area from multi-temporal image data.

Table 5. Training and validation samples

	Tea	Not tea	
		Forest	Corpland
Training	990	2927	2003
~~Validation~~Testing	303	1254	854

Results

Extraction and result assessment

After the classification and accuracy assessment stage, tea plantation area (*Fig 9,10*) and accuracy (*Table ~~5~~6*) were obtained. The CNN result achieve relatively accuracy because of over-fitting problem in small sample case. The loss the training loss is converging to 0.01, while the validation loss is still not converging, causing low accuracy in validation set and even low accuracy in test set. The ~~maximum likelihood~~maximum likelihood results had the lowest ~~producer~~ user accuracy and Kappa coefficients because some other vegetation was mis-classified as tea. This misclassification was successfully minimized by the ~~CART~~ SVM algorithm, which improved the ~~producer~~ user accuracy of tea to ~~84~~87.7%. However, the producer accuracy of tea of SVM results slightly lower then maximum likelihood and CART results. ~~However~~Since~~,~~ pine trees are widespread in the study area; ~~because~~ which have similar the texture with ~~of these trees is quite similar as that of~~ tea, it is difficult to separate this vegetation from tea in optical remote sensing data. Thus, misclassification occurred between these two vegetation types, causing low ac-curacy, but the method could effectively separate tea from other vegetation, especially wood.

Table ~~6~~5~~.~~ Accuracy assessment of 2017 classification result

~~classifier~~	Maximum likelihood	CART	CNN	SVM
**Overall accuracy**	86.15%	90.75%	86.7%	93.14%
**Kappa coefficient**	0.67	0.76	0.65	0.81
**Producer accuracy (tea)**	93.28%	88.02%	76.66%	83.81%
**Producer accuracy (other vegetation)**	83.83%	91.63%	90.01%	96.16%
**User accuracy (tea)**	65.28%	77.43%	71.43%	87.7%
**User accuracy (other vegetation)~~User accuracy (tea)~~**	97.45%	95.91%	92.2%	94.81%

Formatted: 05 Main Section Title, Left, Indent: First line: 0 ch, Line spacing: single

Formatted: Font: 16 pt, Not Italic, English (United States)

Formatted: 06 Main Text Format, Line spacing: single, Tab stops: Not at 3" + 6.5"

Formatted: Font: Not Italic

Formatted: Font: Bold, Not Italic

Formatted Table

Formatted: Font: 10 pt, Bold

Formatted: 06 Main Text Format, Centered

Formatted: Font: Bold

Formatted: Font: 10 pt, Bold

Formatted: 06 Main Text Format, Centered

Formatted: Font: Bold

Formatted: Font: 10 pt, Bold

Formatted: 06 Main Text Format, Centered

Formatted: Font: Bold

Formatted: Font: 10 pt, Bold

Formatted: 06 Main Text Format, Centered

Formatted: Font: Bold

Formatted: Font: 10 pt, Bold

Formatted: 06 Main Text Format, Centered

Formatted: Font: Bold

Formatted: Font: 10 pt, Bold

Formatted: 06 Main Text Format, Centered

Formatted: Font: Bold

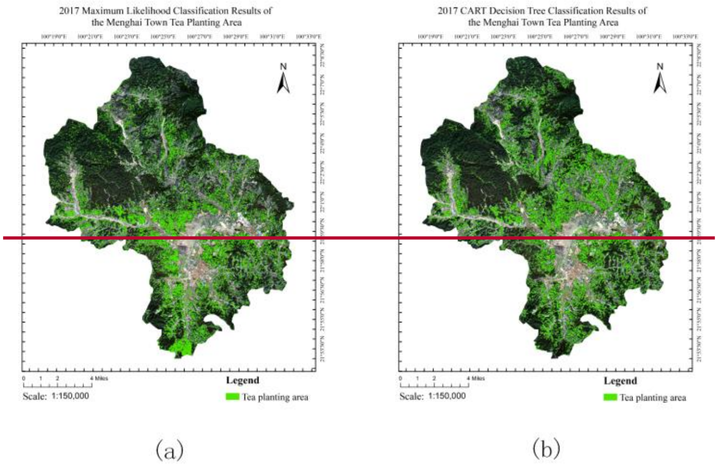

Fig~~ure~~ 9.~~Maximum likelihood~~ Tea extraction result. The base map of the LandSat8 OLI images with the following composition:R (4),G (3) and B (2).The LandSat8 OLI images were downloaded from USGS National Map Viewer.~~and CART decision tree~~

Then, ~~this~~ proposed method was utilized to extract tea from three periods in 2014, 2015, and 2016, and the results contained tea-growing areas (*Fig.* 10). To examine the accuracy of the results, the traditional accuracy assessment method was used to generate a confusion matrix (*Table 6*). ~~According to the analysis of the confusion matrix, the accuracies of 2014 and 2015 were lower than the accuracy of 2016 because of the difficulty of sample selection. Field data are difficult to obtain, while tea area is difficult to identify by visual interpretation in GF-1 image data.~~ Some extracted tea areas were ~~located~~ in the deep forest, but most tea was normally planted at the edge of a forest and in the neighborhood of the residential area during field research. However, the result accuracy was satisfactory for applications.

Table ~~7~~6. Accuracy assessment of ~~2017~~ 2014-2016 SVM classification result

~~time~~	2014	2015	2016
**Overall accuracy**	~~84~~90.43%	~~86~~90.65%	~~92~~93.14%
**Kappa coefficient**	~~0.62~~0.71	~~0.59~~0.75	~~0.77~~0.81
**Producer accuracy (tea)**	~~64~~87.24%	~~87~~73.84%	~~83~~89%
**Producer accuracy (other vegetation)**	~~84~~91.16%	~~86~~97.31%	~~94~~94.5%
**User accuracy (tea)**	~~94~~69.23%	~~55~~91.6%	~~80~~84.17%
**User accuracy (other vegetation)**	96.6%	90.37	96.31%

Formatted: Font: 11 pt, Bold, English (United Kingdom)
Formatted: (Asian) Chinese (PRC), (Other) English (United Kingdom), Do not check spelling or grammar
Formatted: Font: Bold, (Asian) Chinese (PRC), (Other) English (United Kingdom), Do not check spelling or grammar
Formatted: Font: Bold, Not Italic
Formatted: Font: Not Bold
Formatted: Font: Bold, Not Italic
Formatted: Font: Not Italic
Formatted: Font: Bold, Not Italic
Formatted Table
Formatted: Font: 10 pt, Bold
Formatted: 06 Main Text Format, Centered
Formatted: Font: Bold
Formatted: Font: 10 pt, Bold
Formatted: 06 Main Text Format, Centered
Formatted: Font: Bold
Formatted: Font: 10 pt, Bold
Formatted: 06 Main Text Format, Centered
Formatted: Font: Bold
Formatted: Font: 10 pt, Bold
Formatted: 06 Main Text Format, Centered
Formatted: Font: Bold
Formatted: Font: 10 pt, Bold
Formatted: 06 Main Text Format, Centered
Formatted: Font: Bold
Formatted: Font: 10 pt, Bold
Formatted: 06 Main Text Format, Centered
Formatted: Font: Bold

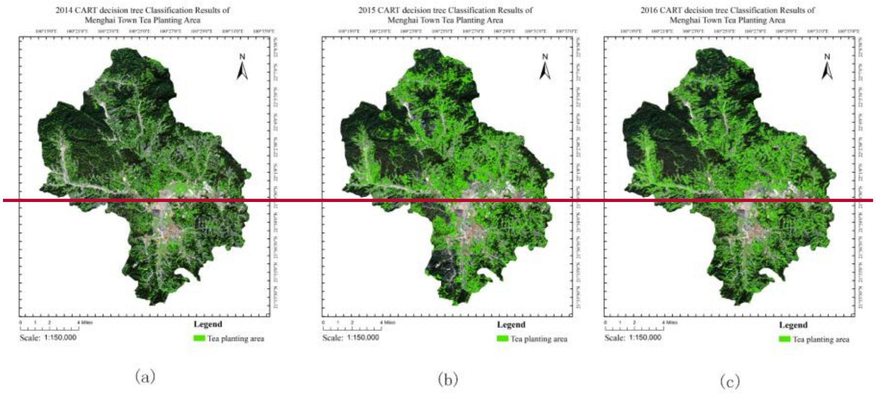

Fig~~ure~~ 10. Extraction results in (a)2014 (b)2015 and (c)2016. The base map of the LandSat8 OLI images with the following composition:R (4),G (3) and B (2).The LandSat8 OLI images were downloaded from USGS National Map Viewer.

Change detection

The ~~eCogntion~~ ENVI software was utilized for change detection, while the area was calculated by Arc ~~RC~~GIS software for statistical analysis. The spatial variation distribution (*Fig. 11*) and area change situation were analyzed (*Table 7*, *Fig. 12*, *13*). The overall pattern of spatial variation is expansion to the mid-west and east. The tea plantation area sharply increased between ~~2014~~ 2015 and ~~2015~~ 2016 and slowly increased between ~~2015~~ 2014 and ~~2016~~ 2015~~but slightly decreased from 2016 to 2017~~. The increasing rate was much slower between 2016 and 2017.

Table ~~8~~7. Tea plantation area changes during 2014-2017

T~~t~~ime	2014	2015	2016	2017
A~~a~~rea/acre	~~14953.65~~13757.64	~~18130.59~~14705.58	~~18006.4~~17765.30	~~18422.41~~17853.00

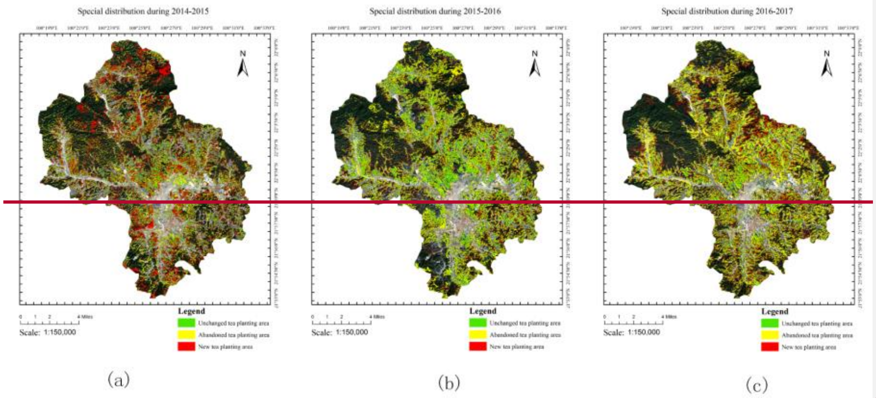

Fig~~ure~~ 11. Spatial distribution during (a)2014-2015 (b)2015-2016 and (c)2016-2017. The base map of the LandSat8 OLI images with the following composition:R (4),G (3) and B (2).The LandSat8 OLI images were downloaded from USGS National Map Viewer.

Formatted: Font: Not Italic
Formatted: Font: Not Bold
Formatted: 10 Figure Titles, Left, Indent: First line: 0 ch, Line spacing: single, Tab stops: Not at 3" + 6.5"
Formatted: Font: 16 pt, Not Italic
Formatted: Font: 16 pt, Bold, Font color: Black, Do not check spelling or grammar
Formatted: Font: Not Italic
Formatted: Font: Bold, Not Italic
Formatted Table
Formatted: Font: Bold
Formatted: (Asian) Chinese (PRC), (Other) English (United Kingdom), Do not check spelling or grammar
Formatted: Font: Bold, (Asian) Chinese (PRC), (Other) English (United Kingdom), Do not check spelling or grammar
Formatted: Font: Bold, Not Italic
Formatted: Font: Not Bold
Formatted: Font: 11 pt, Bold, English (United Kingdom)

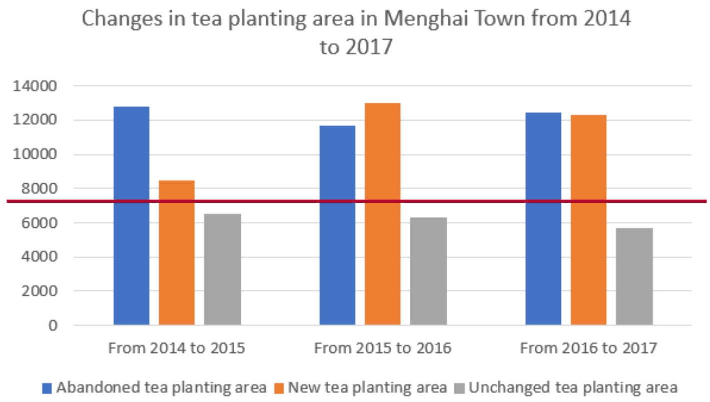

Fig~~ure~~ 12. Spatial variation from 2014 to 2017~~Area change from 2014 to 2017~~

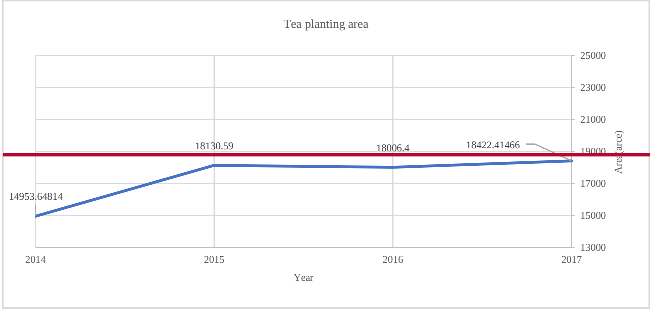

Fig~~ure~~ 13. Area change from 2014 to 2017~~Special variation from 2014 to 2017~~

Discussion

In this study, our primary objective was to analyze and propose a tea plantation mapping method and monitor the tea plantation area in Menghai Town ~~during~~ from 2014 to 2017. ~~The NDVI thershold segmentation metohd was used to extract vegtation area from GF-1 data.~~ The optimal segmentation parameters, such as segmentation scale (SS), weight of spectrum (WS1), weight of shape (WS2), weight of smoothness (WS3), and weight of compactness (WC), were selected by calculate and ana~~y~~lyze the local variance (LV) and the rate of change (ROC) of image homogeneity. The experiment results indicated that the optimal extraction of tea plantation are achieved when SS is set to 66, the WS is set to 0.5, and the WC is set to 0.7. The SS determines the size of the image object, which~~If~~ means ~~SS set~~ higher SS causing larger~~, the~~ image object and missing ~~is too large and~~ details~~ are missing~~. ~~This~~ We ~~finding~~ found ~~indicated~~ that for tea plantation mapping SS can not set too high because of the planting characteristics and vegetation form. WS1 is based on the standard deviation of the spectral colors, while WS2 is based on the deviation of a compact(or smooth) shape. WS1 determines to what degree spectrum influences the segmentation compared to shape, which against WS2. In Menghai town, most tea plantations have regular shape, so high WS2 can achieve better segmentation results. The value of WC gives it a relative weighting against WS3. The

Formatted: English (United States)

Formatted: (Asian) Chinese (PRC), (Other) English (United Kingdom), Do not check spelling or grammar

Formatted: Font: Bold, (Asian) Chinese (PRC), (Other) English (United Kingdom), Do not check spelling or grammar

Formatted: Font: Bold, Not Italic

Formatted: Font: 11 pt, Bold, English (United Kingdom)

Formatted: (Asian) Chinese (PRC), (Other) English (United Kingdom), Do not check spelling or grammar

Formatted: Font: Bold, (Asian) Chinese (PRC), (Other) English (United Kingdom), Do not check spelling or grammar

Formatted: Font: Bold, Not Italic

Formatted: Font: 11 pt, Bold, English (United Kingdom)

plantation form of tea in Menghai town is relatively compact, which make properly higher WC segment tea plantation better[21].

In order to build feature space for image objects to utilize the classification algorithm, SEaTH method is used to calculate the J-M distance of both spectrum~~l~~ and texture features between tea plantation and other vegetations. The experiment results showed that texture features, such as GLCM and GLDV, have the highest J-M distance~~, and most of them are higher than 1.5~~. Since tea plantation in this area have more regular plantation form which significantly different with other vegetation, texture features are more suitable to separate tea from other vegetation. ~~The highest one is GLCM in angle 135°, which is 1.80.~~ Besides,~~ ~~ the mean value of the red band also have a relatively high J-M distance. Because vegetation coverage in tea plantation area normally lower than other vegetation in this area such as forest, the absorption effect of the red band in tea plantation area is relatively lower than other vegetation, causing high discrimination in the mean value of red band [22].

Then decorrelation method was used to remove the correlated features by calculating~~e~~ the correlation coefficients in order to build feature space without ~~-~~redundancy. The results indicated that although J-M distance of most texture features are high, the correlation coefficients are also high, which causes redundancy. Figure 14 shows the correlation coefficients of some texture features. ~~The results indicated that the mean of GLCM in angle 90°, area standard deviation, mean of red band and GLCM ASM have low corelation coefficients and relatively high J-M distance, which can be used to build features to utilize the classification methods.~~

Fig 14. Correlation coefficients of texture features

The accuracy of ~~The~~ maximum likelihood algorithm, CRAT algorithm~~ and~~, CNN-based method(Tang et al.,2020) and ~~maximum likelihood~~SVM algorithm ~~was~~ were ~~compared~~ evaluated at data in 2017 to find the most suitable algorithm~~, using the builded feature space~~. Besides, the performing of binary classification(tea and other vegetation) and multi-classification(tea, cropland and forest) was assessed. Table 9 shows the accuracy assessment results. ~~The experiments results showed that CRAT~~ SVM with multi-classification~~algorithm~~ achieve~~e~~ing ~~92~~93.14% overall accuracy ~~and~~ , 0.81 k~~K~~appa coefficient and 87.7% user accuracy outperforming~~ed~~ ~~maximum likelihood algorithm 4% in overall accuracy and 0.06 in Kappa coefficient~~other methods. The experiment results also indicates that multi-classification method achieving better accuracy than binary classification method in all methods. Multi-classification CART improves 4.15% of overall accuracy and 9.97% of user accuracy on binary classification, which higher than SVM, because SVM only uses support vector to build classifier, which are more robust than CART in binary classification. Besides, multi-classification CNN slightly improves the accuracy of tea extraction on binary classification CNN, which are 0.01 of kappa coefficient and 4.16% of producer accuracy of tea. ~~These findings clearly showed that CART algorithm is more sutiable for the tea plantation mapping, and the combination of special feature, spectral feature and texture feature can achieve promising results in tea plantation mapping.~~

Table 9. Accuracy assessment of 2017 binary classification and multi-classification result ~~Correlation coefficients of texture features~~

~~C~~	Maximum likelihood(binary)	Maximum likelihood(multi)	CART (binary)	CART (multi)	CNN (binary)	CNN (multi)	SVM (binary)	SVM (multi)

Formatted: (Asian) Chinese (PRC), (Other) English (United Kingdom), Do not check spelling or grammar

Formatted: Font: Bold, (Asian) Chinese (PRC), (Other) English (United Kingdom), Do not check spelling or grammar

Formatted: Font: Not Italic

Formatted: Font: Bold, Not Italic

Formatted: Font: Bold, Not Italic, English (United Kingdom)

Formatted: Font: Bold, Not Italic

Formatted Table

**Overall accuracy**	82.5%	86.15%	86.6%	90.75%	86.9%	86.7%	91.94%	93.14%
**Kappa coefficient**	0.60	0.67	0.66	0.76	0.64	0.65	0.77	0.81
**Producer accuracy (tea)**	93.35%	93.28%	86.60%	88.02%	72.5%	76.66%	79.87%	83.81%
**Producer accuracy (other vegetation)**	78.97%	83.83%	86.40%	91.63%	91.6%	90.01%	95.87%	96.16%
**User accuracy (tea)**	59.12%	65.28%	67.46%	77.43%	73.7%	71.43%	86.31%	87.7%
**User accuracy (other vegetation)**	97.32%	97.45%	95.18%	95.91%	91.1%	92.2%	93.6%	94.81%

Then, the best performing algorithm was used to mapping tea plantation at 2014 to 2017. Accuracy assessment was done by confusion matrix and kappa coefficient(*Table 6*). All overall accuracy were above ~~80~~90%, which proves the effectiveness of proposed method.

Conclusion

Menghai Town of Xishuangbanna Dai Autonomous Prefecture, Yunnan Province, Chian,was chosen as the study area, and GF-1 remotely sensed data from 2014 to -2017 were chosen as the data source. Image texture, spectral and geometry features were integrated, while feature space was built by separability and thresholds algorithms (SEaTH) with decorrelation. Object-oriented image analysis (OOIA) with ~~classification and a regression tree (CART)~~SVM algorithm ~~were~~ was utilized to extract tea plantation areas. The salient findings of the study are as follows:

- OOIA can successfully suppress~~ed~~ noise by object-based classification and improve the effect of extraction.
- The selection of segmentation scale and parameters is a critical factor in OOIA. LV and ROC can be used as criteria to evaluate parameters in order to generate meanful image objects. ~~A s~~Scale parameter that is too small causes over segmentation, while a scale that is too large scale mixes different earth surface features in one image object.
- The feature ~~set~~ space built by the SEaTH algorithm and decorrelation with texture features can effectively avoid spectral confusion.
- OOIA combined with the ~~CART decision tree~~SVM algorithm and texture features solves the misclassification and missing-classification issues, yielding a Kappa coefficient up to 0.81 and an overall accuracy of ~~82~~93.14%, higher than ~~the maximum~~

Formatted: Font: Bold, (Asian) Chinese (PRC)

Formatted: Font: Bold, (Asian) Chinese (PRC)

Formatted: Font: Bold, (Asian) Chinese (PRC)

Formatted: Font: Bold, (Asian) Chinese (PRC)

Formatted: Font: Bold, (Asian) Chinese (PRC)

Formatted: Font: Bold, (Asian) Chinese (PRC)

Formatted: Font: 18 pt

likelihoodCART algorithmmethod. Multi-classification method achieve better performance than binary classification in most algorithm.

- According to the extraction results, tea plantation areas are mainly distributed in the neighborhoods of residential areas with moderate slopes, –because tea must be cultivated in easily drained areas and be convenient for farmers to reach. Based on variation analysis, we found that tea plantations expanded towards areas with sparse forests and moderate slopes.

Expectations

The extraction method of tea plantations based on RS still needs to be discovered. Currently, methods still have flaws in accuracy and efficiency. In consideration of this, we will focus on the following aspect in feature research:

- ESP is utilized to select the segmentation scale, but other parameters still need to be selected based on a visual assessment. The parameter selection method can be improved by automatically selecting the weight of the spectrum and the weight of compactness based on LV to achieve the optimal segmentation result.
- According toIn order to overcome the texture and spectral confusion, different data and extraction methods can be used: consider the growing period of tea based on low revisit period RS data; multi-source data fusion such as hyperspectral and high-resolution RS data to analyze the spectral and texture features; integrate with LiDAR data to obtain canopy height and shape information to reduce spectrum fixing; integrate novel classification algorithms such as artificial intelligence and machine learning to improve accuracyuse few sample learning method to overcome overfitting problem of deep learning and CNN.

Acknowledgements.

This work was supported by Multi-government International Science and Technology Innovation Cooperation Key Project of National Key Research and Development Program of China , grant number 2018YFE0184300; the National Natural Science Foundation of P.R. China, grant number 41561048; the Program for Innovative Research Team (in Science and Technology) in the University of Yunnan Province, IRTSTYN; and the Undergraduate Research Innovation Foundation of Yunnan Normal University. The authors are grateful to the editor and anonymous referees for their valuable comments and helpful suggestions. We wish to thank professor Cheng Wang and Xiaohuang Xi for their advice.

ReferencesReferences

1. Li Z, He LQ, Tea and Meteorology. 1st ed. Beijing, China: Meteorological Press; 2005. pp. 25-26.

Formatted: Font: 18 pt, Not Italic, (Asian) Chinese (PRC), (Other) Hungarian, Kern at 12 pt

Formatted: Font: 18 pt

Formatted: Font: 12 pt, (Asian) Chinese (PRC), (Other) Hungarian, Do not check spelling or grammar

Formatted: Indent: First line: 0.5"

Formatted: Font: 12 pt, (Asian) Chinese (PRC), Do not check spelling or grammar

Formatted: Font: 12 pt, (Asian) Chinese (PRC), Do not check spelling or grammar

Formatted: Font: 12 pt, (Asian) Chinese (PRC), Do not check spelling or grammar

Formatted: Font: 12 pt, (Asian) Chinese (PRC), (Other) Hungarian, Do not check spelling or grammar

Formatted: Font: 12 pt, (Asian) Chinese (PRC), Do not check spelling or grammar

Formatted: 04 Text of Abstract + Félkövér Char, Not All caps

2. Dihkan M, Guneroglu N, Karsli F. Remote sensing of tea plantations using an SVM classifier and pattern-based accuracy assessment technique. International Journal of Remote Sensing. 2013; 34(23): 8549-8565.
3. Xu WY, Sun R, Jin ZF. Extracting tea plantations based on ZY-3 satellite data. Transactions of the Chinese Society of Agricultural Engineering (Transactions of the CSAE). 2016; 32(Supp.1): 161-168.
4. Rajapakse RS, Tripathi NK, Honda K. Spectral characterization and LAI modelling for the tea (Camellia sinensis (L.) O. Kuntze) canopy. International Journal of Remote Sensing. 2002; 23(18), 3569-3577.
5. He L, Kong F, Shen Z. Artificial Neural Network Ensemble for Land Cover Classification, World Congress on Intelligent Control & Automation 2006.
6. Li, Z, Li, Z. Mapping the Spatial Distribution of Tea Plantations Using High-Spatiotemporal-Resolution Imagery in Northern Zhejiang. Forests. 2019; 10(10): 856.
7. Jun Z, Ziwu P, Hang W, Peijie H, Jiulin S, Fen Q, et al. An Improved Multi-temporal and Multi-feature Tea Plantation Identification Method Using Sentinel-2 Imagery. Sensors. 2019; 19(9): 2087.
8. Mallinis G, Koutsias N, Tsakiri-Strati M, et al. Object-based classification using Quickbird imagery for delineating forest vegetation polygons in a Mediterranean test site. ISPRS Journal of Photogrammetry & Remote Sensing. 2008; 63(2), 237-250.
9. Zixia T, Mengmeng L Xiaoqin W. Mapping Tea Plantations from VHR Images Using OBIA and Convolutional Neural Networks. Remote Sensing. 2020; 12(18): 2935.
10. Wei YH. Remote Sensing Digital Image Processing Tutorial, 2nd ed. Beijing, China: Science Press; 2015. pp. 185-304.
11. Sun JB. Principle and Application of Remote Sensing, 3rd ed. Wuhan, China: Wuhan University Press; 2013. pp. 108-224.
12. Deng SB, Chen QJ, Du HJ. ENVI Remote Sensing Image Processing Method, 2nd ed. Beijing, China: Beijing Higher Education Press; 2014. pp. 132-228,
13. Gonzalez RC, Woods RE, Eddins SL. Digital Image Processing Using MATLAB, 2nd ed.Beijing, China: Publishing House of Electronics Industry; 2009. pp. 135-136.
14. Otsu N. A Threshold Selection Method from Gray-Level Histograms. IEEE Transactions on Systems Man, and Cybernetics. 1979; 9(1), 62-66.
15. Liu JH. Mao ZY. Review of High Spatial Resolution Remote Sensing Image Segmentation Methods. Remote Sensing Information. 2009; 11(6): 95-101.
16. Drǎgu, L, Tiede D, Levick SR. ESP: a tool to estimate scale parameter for multiresolution image segmentation of remotely sensed data. International Journal of Geographical Information Science. 2010; 24(6): 859-871.
17. Zhang J. Classification and Feature Selection on High-dimensional and Small-sampling Data. 2014; Hefei University of Technology.
18. Dong LY, Xing LX, Pan J. Vegetation Classification in Hyper-spectral Image with CART Decision Tree. Journal of Jilin University Information Science Edition. 2013; 1: 83-89.
19. Jiang HW, Ren HQ, Qin XF. Auto Interpretation and Accuracy Evaluation of Images Based on eCognition. Bulletin of Surveying and Mapping. 2015; 10, 81-84.
20. Sven N, Gunter M. Object-Based Image Analysis and Treaty Verification. Springer Netherlands. 2008; 51-62.
21. Benz UC, Hofmann P, Willhauck G. Multi-Resolution, Object-Oriented Fuzzy Analysis of Remote Sensing Data for GIS-Ready Information. ISPRS Journal of Photogrammetry & Remote Sensing. 2004; 58: 239–258.
22. Henchiri M, Liu Q, Essifi B. Spatio-Temporal Patterns of Drought and Impact on Vegetation in North and West Africa Based on Multi-Satellite Data. Remote Sensing. 2020; 12(23): 3869.

~~Deng Shubin, Chen Qiujin, Du Huijian. ENVI Remote Sensing Image Processing Method, 2nd ed.; Beijing Higher Education Press: Beijing, China, 2014, 132-228,~~

Formatted: No bullets or numbering

Supporting Information

S1 File. 2014TeaExtractionResult.

Deng Shubin, Chen Qiujin, Du Huijian. ENVI Remote Sensing Image Processing Method, 2nd ed.; Beijing Higher Education Press: Beijing, China, 2014, 132-228.

Formatted: No bullets or numbering

S2 File. 2015TeaExtractionResult.

S3 File. 2016TeaExtractionResult.

S4 File. 2017TeaExtractionResult.

Formatted: No bullets or numbering

[1] Dihkan M, Guneroglu N, Karsli F. Remote sensing of tea plantations using an SVM classifier and pattern based accuracy assessment technique. International Journal of Remote Sensing 2013, 34(23), 8549-8565.

[2] Dong Lianying, Xing Lixin, Pan Jun. Vegetation Classification in Hyper-spectral Image with CART Decision Tree, Journal of Jilin University, Information Science Edition 2013 ,1, 83-89.

[3] Drǎgu, Lucian, Tiede D, Levick S R. ESP: a tool to estimate scale parameter for multiresolution image segmentation of remotely sensed data, International Journal of Geographical Information Science 2010, 24(6),859-871.

[4] Gonzalez R C, Woods R E, Eddins S L. Digital Image Processing Using MATLAB, 2nd ed.; Publishing House of Electronics Industry: Beijing, China, 2009, 135-136.

[5] He L, Kong F, Shen Z. Artificial Neural Network Ensemble for Land Cover Classification, World Congress on Intelligent Control & Automation 2006.

[6] Jiang Haowei, Ren Hongquan, Qin Xianfeng. Auto Interpretation and Accuracy Evaluation of Images Based on eCognition, Bulletin of Surveying and Mapping 2015 ,10, 81-84.

[7] Jun Zhu, Ziwu Pan, Hang Wang, Peijie Huang, Jiulin Sun, Fen Qin, Zhenzhen Liu. An Improved Multi-temporal and Multi-feature Tea Plantation Identification Method Using Sentinel-2 Imagery, Sensors 2019, 19(9), 2087.

[8] Li Zhuo, He Lingqi, Tea and Meteorology, 1st ed.; Meteorological Press: Beijing, China, 2005; 25-26.

[9] Li, Zhang, Li, Zhang. Mapping the Spatial Distribution of Tea Plantations Using High-Spatiotemporal-Resolution Imagery in Northern Zhejiang 2019, Forests 10:10, 856.

[10] Liu Jianhua. MAO Zheng-yuan. Review of High Spatial Resolution Remote Sensing Image Segmentation Methods, Remote Sensing Information 2009, 95-101.

[11] Liu Shuhan, Gu Xingfa, Yu Tao. Object-Oriented Classification method based on GF-1 Multi-spectral Remote Sensing image, Surveying Science 2014,12, 91-103.

[12] Mallinis G, Koutsias N, Tsakiri-Strati M, et al. Object-based classification using Quickbird imagery for delineating forest vegetation polygons in a Mediterranean test site, ISPRS Journal of Photogrammetry & Remote Sensing 2008, 63(2), 237-250.

[13] Otsu N. A Threshold Selection Method from Gray-Level Histograms, IEEE Transactions on Systems, Man, and Cybernetics 1979, 9(1), 62-66.

[14] Rajapakse R M S S, Tripathi N K, Honda K. Spectral characterization and LAI modelling for the tea (Camellia sinensis (L.) O. Kuntze) canopy, International Journal of Remote Sensing 2002, 23(18), 3569-3577

[15] Sun Jiabing. Principle and Application of Remote Sensing, 3rd ed.; Wuhan University Press: Wuhan, China, 2013, 108-224.

[16] Sven Nussbaum, Gunter Menz. Object-Based Image Analysis and Treaty Verification, Springer Netherlands 2008,51-62.

[17] Wei Yuchun. Remote Sensing Digital Image Processing Tutorial, 2nd ed.; Science Press: Beijing, China, 2015, 185-304.

[18] Xu Weiyan, Sun Rui, Jin Zhifeng. Extracting tea plantations based on ZY-3 satellite data, Transactions of the Chinese Society of Agricultural Engineering (Transactions of the CSAE) 2016, 32(Supp.1), 161-168.

[19] Zhang Jing. Classification and Feature Selection on High-dimensional and Small-sampling Data 2014, Hefei University of Technology.

[20] Zixia Tang, Mengmeng Li, Xiaoqin Wang. Mapping Tea Plantations from VHR Images Using OBIA and Convolutional Neural Networks. Remote Sensing 2020, 12:18, pages 2935.

Response to Reviewer #1 comments:

Dear Editors and Reviewer:

Thank you for your comments concerning our manuscript entitled “Mapping Pu'er tea plantations from GF-1 images using OOIA and SVM” (ID: PONE-D-21-11402R1). Those comments are valuable and helpful for revising and improving our paper, as well as the important guiding significance to our researches. We have studied comments carefully and have made correction which we hope meet with approval. The responses to the reviewer’s comments are as follows:

* 1. The title of this paper need to be revised. "Mapping Pu'er tea plantations from GF-1 images using Object-oriented image analysis (OOIA) andclassification and regression tree (CART)". A space character shoud be inserted between the word "and" and "classification"!!!

Response 1: Thanks for you remind. We have revised it.

* 2. In the abstract, "The overall accu-racy and Kappa coefficient". Why the "-" is inserted into the word "accuracy"? There are many similar mistakes in the full text. Please revise these errors carefully ! !

Response 2: Thanks for you remind. We have carefully checked the full text and revised them.

* 3. In line 88, "of this town is 365.38,, of which 45.3 88 ,..." the redundant commas should be removed!!

Response 3: Thanks for you remind. We have removed it.

* 4. In line 108, "from vegetation area Third step was multiresolution segmentation", where

is the full stop between “area” and "Third"??

Response 4: Thanks for you remind. We have added it.

* 5. The language of the paper needs to be retouched

Response 5: We have thoroughly retouched the language of the paper.

* 6. Why do you just compare the performance of maximum likelihood and CART? why not support vector machine or the convolutional neural network (CNN) as you mentioned in the introduction section. CNN has been widely used in crop planting area extraction. What are the advantages of your method ?

Response 6: Thanks for you advises. We have added the comparison of CNN and SVM. In the experiment of CNN, we used method proposed by Tang [1], which used a fine-tuned ResNet-50 to extract feature from image object and classified them by random forest. However, we found CNN is suffering overfitting owing to small samples. Even we have used dropout as anti-overfitting method to gain more generalization ability, the experiment result shows that overfitting is still hard to overcome due to lack of samples. In fig 1 (a), the training loss is converging to 0.01, while the validation loss is still not converging, causing low accuracy in validation set and even low accuracy in test set. The producer accuracy and user accuracy of tea in 2017 is 76.66% and 71.34%. Besides, CNN method is relatively slow during the training phase, and the best-performance hyper-parameters require lots of time to search and compare. In the future, we will focus on small sample deep learning and transfer learning in tea plantation extraction in order to utilize the powerful classification ability of deep learning.

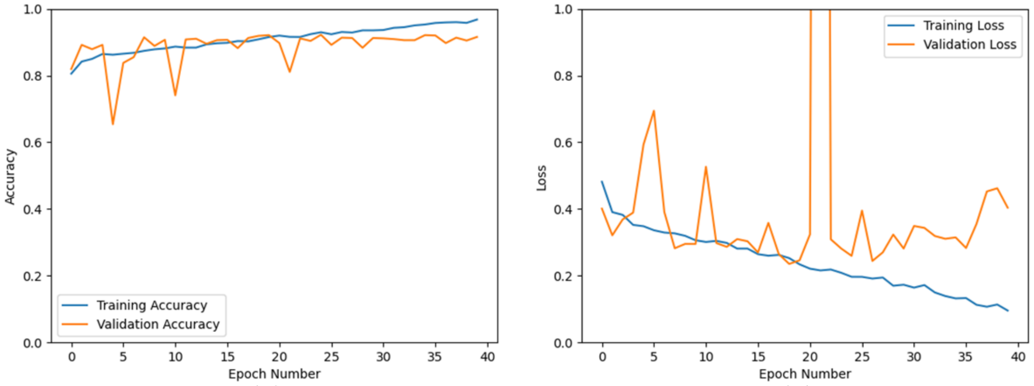

(a) (b)

Figure 1. Loss and accuracy of training and validation

In the experiment of SVM, we found SVM achieved better performance than CART, CNN and maximum likelihood. Therefore, we chose SVM as classifier to classify image object. SVM is more suitable for small sample learning, since it only using supporting vectors to construct classifier. We normalized the feature to 0-1 and used Radial Basis Function (RBF) as kernel function of SVM. Besides, on account of the suggestion given by another reviewer, we divided other vegetation to forest and cropland to achieve better performance during classification and finally combined them into one class. Table 1 shows the accuracy assessment result.

Experiment details and results analysis have been revised and updated in the paper (Line 211-250, Line 269-286, Line346-359, Line403-404).

Table 1. *Accuracy assessment of 2017 classification result*

**Classifier**	**Maximum likelihood(binary)**	**Maximum likelihood(multi)**	**CART (binary)**	**CART (multi)**	**CNN (binary)**	**CNN (multi)**	**SVM (binary)**	**SVM (multi)**
Overall accuracy	82.5%	86.15%	86.6%	90.75 %	86.9 %	86.7 %	91.94%	93.14 %
Kappa coefficient	0.60	0.67	0.66	0.76	0.64	0.65	0.77	0.81
Producer	93.35%	93.28%	86.60%	88.02 %	72.5 %	76.66%	79.87%	83.81 %

accuracy (tea)								
Producer accuracy (other vegetation)	78.97%	83.83%	86.40%	91.63%	91.6%	90.01%	95.87%	96.16%
User accuracy (tea)	59.12%	65.28%	67.46%	77.43%	73.7%	71.43%	86.31%	87.7%
User accuracy (other vegetation)	97.32%	97.45%	95.18%	95.91%	91.1%	92.2%	93.6%	94.81%

** 7. The legends should be added in Figure 6-8 to illustrate the segmentation results.*

Response 7: Thanks for your suggestion. We have added it.

Response to Reviewer #3 comments:

Dear Editors and Reviewer:

Thank you for your comments concerning our manuscript entitled “Mapping Pu'er tea plantations from GF-1 images using OOIA and SVM” (ID: PONE-D-21-11402R1). Those comments are valuable and helpful for revising and improving our paper, as well as the important guiding significance to our researches. We have studied comments carefully and have made correction which we hope meet with approval. The responses to the reviewer’s comments are as follows:

* 1. The authors should read the manuscript and clarify the command clearly. The authors have to care about typos. There are careless mistakes in some places. Example: environ-ments, moni-toring, distribu-tion, accu-racy, …

Response 1: Thanks for you remind. We have carefully checked the full text and revised them.

* 2. The quality of Figure 1 is poor. Please improve it.

Response 2: Thanks for you remind. We have improved it.

* 3. L105-114: The authors should better explain better the methodology to analyze the tea plantations.

Response 3: Thanks for your suggestion. We have revised our explanation of methodology.

* 4. The authors mentioned that all SS and other parameters were traversed to integrate ESP and visual assessment to select optimal parameters. Please explain the correlation between the SS, WS, and WC parameters? Add some references.

Response 4: Thanks for your suggestion. The SS determines the size of the image object,

higher SS achieve bigger image objects. WS1 is based on the standard deviation of the spectral colors, while WS2 is based on the deviation of a compact (or smooth) shape. WS1 determines to what degree spectrum influences the segmentation compared to shape, which against WS2. In the same way, the value of WC gives it a relative weighting against WS3. We have added those explanation in the paper(L317-326).

* 5. L306-312: The discussion section should be the focus on each of the parametric discussed as confidential factors. You can add some references. Remote Sensing. 2020, 12, 3869. https://doi.org/10.3390/rs12233869; Envi. Sci. Pull. Resea. 27(6):5873-5889. DOI:10.1007/s11356-019-07216-1

Response 5: We have thoroughly revised discussion section, adding more references, result analysis and discussion.

* 6. The quality of all the figures should be improved.

Response 6: Thanks for you remind. We have improved them all.

* 7. In the Conclusion section, the authors should be re-writing some sentences (L323-L353).

Response 7: Thanks for your suggestion. We have revised those sentences.

Response to Reviewer #2 comments:

Dear Editors and Reviewer:

Thank you for your comments concerning our manuscript entitled "Mapping Pu'er tea plantations from GF-1 images using OOIA and SVM" (ID: PONE-D-21-11402R1). Those comments are valuable and helpful for revising and improving our paper, as well as the important guiding significance to our researches. We have studied comments carefully and have made correction which we hope meet with approval. The responses to the reviewer's comments are as follows:

* 1. Throughout the paper, please carefully check and correct grammatical errors and typos.

Response 1: Thanks for your suggestion. We have carefully revised them.

* 2. Line 120, some explanations about NDVI should be revised. Please see my comment in the attached file.

Response 2: Thanks for you remind. We have revised it.

* 3. I Line 96, some of the technical terms are misspelled.

Response 3: Thanks for you remind. We have revised it.

* 4. The main drawback of the paper is the lack of appropriate discussion. Some presented reasons about why these results were achieved are not convincing. Discussion section provides a summary of work instead of discussion.

Response 4: Thanks for your suggestion. We have rethought and reorganized our discussion section, making more analysis and explanations about results.

* 5. Details of texture implementation such as window size used are not mentioned.

Response 5: Thanks for your suggestion. Since we used OOIA method to analyze the

image, the textures were calculated inside the image object, using all pixels inside the image object. We have added this explanation in the paper (Line232-233).

* 6. Details of the accuracy assessment, such as the number of training samples and how they were gathered, are not given.

Response 6: Thanks for your suggestion. The training samples were gathered by field research and visual interpretation. We split it 80/20 to do training and accuracy assessment. The table of samples condition was shown in table 1 **(Table 5 in revised draft)**. Detailed explanation has added to the paper (Line 258-265).

Table 1*. Training and validation samples*

	Tea	Not tea	
		**Forest**	**Corpland**
Training	990	2927	2003
Testing	303	1254	854

* 7. Line 30. I do not know exactly what you mean by "manual method"! However, Field measurements are usually considered as the most precise method. Do you have a reference for this claim?

Response 7: Thanks for your suggestion. Manual method means visual interpretation from remote sensing image. We have revised this sentence (Line32-33).

* 8. Line 41. The accuracy of vegetation information extraction results depends more on the data used than on the method used. Please indicate what kind of data/ satellite images were used.

Response 8: Thanks for your suggestion. We have added the kind of data/ satellite images were used (Line42-62).

* 9. Figure 4. Please present what image with what color composite has been depicted here.

Response 9: Thanks for you remind. Due to copyright problem editor posed, the GF-1 images can not be use. So, we change the base map of figure4 to Landsat 8 OLI. The color composition of image is R (4), G (3) and B (2). We have revised it.

* 10. Line 235. were the classifications binary? i.e. considering only tea and non tea. In the case of classification of spectrally similar classes, multi-class classification often provides better results than binary one. The classes which are not the target class can be finally combined into one class (i.e. non- tea class). How many training samples were chosen for training step?

Response 10: Thanks for your suggestion. Yes, the classifications were binary. Given your advice, we have divided other vegetation by two classes, which are cropland and forest. In order to build better feature space for multi-class classification, we recalculate the J-M distance between tea and both of them. The J-M distance and correlation coefficient of new feature space were shown in table 2. The detailed analysis and discussion have added in the paper (Line 210-250).

Table3 shows the accuracy assessment of classification results. We found that multi-class classification achieved better performance in both methods. Besides, on account of the suggestion given by another reviewer, we compared the SVM algorithm and CART algorithm. The results shown that SVM achieved better performance than CART. The number of training samples is shown in table1. The experiment details and result analysis were updated in the article (Line 267-285,

Line 345-358).

Table 2 *correlation coefficients and J-M distances of feature set*

	**GLCM homogeneity**	**Width**	**GLDV mean**	**Max. diff**	**GLCM dissimilarity**	**GLCM StdDev**	**Number of neighbors**	**Stddev of area represented by segments**	**Red band mean**	**GLCM ASM**
J-M distance (tea and forest)	0.85	0.04	0.21	0.76	0.21	0.09	0.01	0.68	1.06	0.16
J-M distance (tea and corpland)	0.44	0.86	0.76	0.20	0.76	0.78	0.91	0.54	0.75	0.89
GLCM homogeneity	1.00	0.50	-0.71	0.03	-0.71	-0.64	0.34	0.65	-0.01	-0.42
Width	0.50	1.00	-0.63	0.40	-0.63	-0.72	0.81	0.53	-0.49	-0.34
GLDV mean	-0.71	-0.63	1.00	-0.43	1.00	0.84	-0.55	-0.49	0.35	0.72
Max. diff	0.03	0.40	-0.43	1.00	-0.43	-0.59	0.52	0.00	-0.74	-0.30
GLCM dissimilarity	-0.71	-0.63	1.00	-0.43	1.00	0.84	-0.55	-0.49	0.35	0.72
GLCM StdDev	-0.64	-0.72	0.84	-0.59	0.84	1.00	-0.67	-0.50	0.53	0.53

Number of neighbors	0.34	0.81	-0.55	0.52	-0.55	-0.67	1.00	0.34	-0.58	-0.31
Stddev of area represented by segments	0.65	0.53	-0.49	0.00	-0.49	-0.50	0.34	1.00	-0.04	-0.23
Red band mean	-0.01	-0.49	0.35	-0.74	0.35	0.53	-0.58	-0.04	1.00	0.19
GLCM ASM	-0.42	-0.34	0.72	-0.30	0.72	0.53	-0.31	-0.23	0.19	1.00

Table 3. Accuracy assessment of classification results

**Classifier**	**Maximum likelihood(binary)**	**Maximum likelihood(multi)**	**CART (binary)**	**CART (multi)**	**CNN (binary)**	**CNN (multi)**	**SVM (binary)**	**SVM (multi)**
Overall accuracy	82.5%	86.15%	86.6%	90.75%	86.9%	86.7%	91.94%	93.14%
Kappa coefficient	0.60	0.67	0.66	0.76	0.64	0.65	0.77	0.81
Producer accuracy (tea)	93.35%	93.28%	86.60%	88.02%	72.5%	76.66%	79.87%	83.81%
Producer accuracy (other vegetation)	78.97%	83.83%	86.40%	91.63%	91.6%	90.01%	95.87%	96.16%
User accuracy (tea)	59.12%	65.28%	67.46%	77.43%	73.7%	71.43%	86.31%	87.7%
User accuracy (other	97.32%	97.45%	95.18%	95.91%	91.1%	92.2%	93.6%	94.81%

vegetation)	

* 11. Line 250. This defect can be largely eliminated by the use of LiDAR data.

Response **11**: Thanks for your suggestion. Since the LiDAR data can obtain canopy height and shape information, it can separate spectrally similar vegetation. However, airborne LiDAR data are hard to obtain in large area such as whole county. Meanwhile, space-borne LiDAR data such as ICESAT/GLAS, ICESAT-2 and GEDI have limited discrete coverage. The number of valid data in research area are quite limited, which makes space-borne LiDAR data hard to use in tea plantation extraction. In the future, we will be focus on multi-source data fusion method in order to use discrete covered space-borne LiDAR data to achieve better performance. We have added those as expectation in the paper (Line 401-402).

* 12. Line 261-262. I'm not fully convinced that this is the reason of the low accuracies of 2014 and 2015. I think it can be related to the features used, themselves. This is also true in the case of the other years.

Response 12: Thanks for your suggestion. Based on your suggestion, we have improved our feature space, adding more feature including spectrum, texture and geometry. The accuracies of 2014 and 2015 are improved. Experiments details and results have illustrated in the paper (Line210-249, Line 292-293).
