# Supplementary material for: Mapping Pu’er tea plantations from GF-1 images using Object-Oriented Image Analysis (OOIA) and Support Vector Machine (SVM)
Source: PLoS One. 2023 Feb 7;18(2):e0263969. doi: 10.1371/journal.pone.0263969 (PMC9904455; doi:10.1371/journal.pone.0263969)
Supplement: Attachment — Submitted filename: Response to Reviewer2.docx [file pone.0263969.s003.docx]

**Response to Reviewer #2 comments:**

Dear Editors and Reviewer:

Thank you for your comments concerning our manuscript entitled “Mapping Pu'er tea plantations from GF-1 images using OOIA and SVM” (ID: PONE-D-21-11402R1). Those comments are valuable and helpful for revising and improving our paper, as well as the important guiding significance to our researches. We have studied comments carefully and have made correction which we hope meet with approval. The responses to the reviewer’s comments are as follows:

* 1. Throughout the paper, please carefully check and correct grammatical errors and typos.

Response 1: Thanks for your suggestion. We have carefully revised them.

* 2. Line 120, some explanations about NDVI should be revised. Please see my comment in the attached file.

Response 2: Thanks for you remind. We have revised it.

* 3. I Line 96, some of the technical terms are misspelled.

Response 3: Thanks for you remind. We have revised it.

* 4. The main drawback of the paper is the lack of appropriate discussion. Some presented reasons about why these results were achieved are not convincing. Discussion section provides a summary of work instead of discussion.

Response 4: Thanks for your suggestion. We have rethought and reorganized our discussion section, making more analysis and explanations about results.

* 5. Details of texture implementation such as window size used are not mentioned.

Response 5: Thanks for your suggestion. Since we used OOIA method to analyze the image, the textures were calculated inside the image object, using all pixels inside the image object. We have added this explanation in the paper (Line232-233).

* 6. Details of the accuracy assessment, such as the number of training samples and how they were gathered, are not given.

Response 6: Thanks for your suggestion. The training samples were gathered by field research and visual interpretation. We split it 80/20 to do training and accuracy assessment. The table of samples condition was shown in table 1 **(****Table 5 in revised draft)**. Detailed explanation has added to the paper (Line 258-265).

***Table 1****. Training and validation samples*

|  | **Tea** | **Not tea** | |
| --- | --- | --- | --- |
|  |  | **Forest** | **Corpland** |
| Training | 990 | 2927 | 2003 |
| Testing | 303 | 1254 | 854 |

* 7. Line 30. I do not know exactly what you mean by "manual method"! However, Field measurements are usually considered as the most precise method. Do you have a reference for this claim?

Response 7: Thanks for your suggestion. Manual method means visual interpretation from remote sensing image. We have revised this sentence (Line32-33).

* 8. Line 41. The accuracy of vegetation information extraction results depends more on the data used than on the method used. Please indicate what kind of data/ satellite images were used.

Response 8: Thanks for your suggestion. We have added the kind of data/ satellite images were used (Line42-62).

* 9. Figure 4. Please present what image with what color composite has been depicted here.

Response 9: Thanks for you remind. Due to copyright problem editor posed, the GF-1 images can not be use. So, we change the base map of figure4 to Landsat 8 OLI. The color composition of image is R (4), G (3) and B (2). We have revised it.

* 10. Line 235. were the classifications binary? i.e. considering only tea and non tea. In the case of classification of spectrally similar classes, multi-class classification often provides better results than binary one. The classes which are not the target class can be finally combined into one class (i.e. non- tea class). How many training samples were chosen for training step?

Response 10: Thanks for your suggestion. Yes, the classifications were binary. Given your advice, we have divided other vegetation by two classes, which are cropland and forest. In order to build better feature space for multi-class classification, we recalculate the J-M distance between tea and both of them. The J-M distance and correlation coefficient of new feature space were shown in table 2. The detailed analysis and discussion have added in the paper (Line 210-250).

Table3 shows the accuracy assessment of classification results. We found that multi-class classification achieved better performance in both methods. Besides, on account of the suggestion given by another reviewer, we compared the SVM algorithm and CART algorithm. The results shown that SVM achieved better performance than CART. The number of training samples is shown in table1. The experiment details and result analysis were updated in the article (Line 267-285, Line 345-358).

***Table 2*** *correlation coefficients and J-M distances of feature set*

|  | **GLCM homogeneity** | **Width** | **GLDV mean** | **Max. diff** | **GLCM dissimilarity** | **GLCM StdDev** | **Number of neighbors** | **Stddev of area represented by segments** | **Red band mean** | **GLCM ASM** |
| --- | --- | --- | --- | --- | --- | --- | --- | --- | --- | --- |
| J-M distance（tea and forest） | 0.85 | 0.04 | 0.21 | 0.76 | 0.21 | 0.09 | 0.01 | 0.68 | 1.06 | 0.16 |
| J-M distance（tea and corpland） | 0.44 | 0.86 | 0.76 | 0.20 | 0.76 | 0.78 | 0.91 | 0.54 | 0.75 | 0.89 |
| GLCM homogeneity | 1.00 | 0.50 | -0.71 | 0.03 | -0.71 | -0.64 | 0.34 | 0.65 | -0.01 | -0.42 |
| Width | 0.50 | 1.00 | -0.63 | 0.40 | -0.63 | -0.72 | 0.81 | 0.53 | -0.49 | -0.34 |
| GLDV mean | -0.71 | -0.63 | 1.00 | -0.43 | 1.00 | 0.84 | -0.55 | -0.49 | 0.35 | 0.72 |
| Max. diff | 0.03 | 0.40 | -0.43 | 1.00 | -0.43 | -0.59 | 0.52 | 0.00 | -0.74 | -0.30 |
| GLCM dissimilarity | -0.71 | -0.63 | 1.00 | -0.43 | 1.00 | 0.84 | -0.55 | -0.49 | 0.35 | 0.72 |
| GLCM StdDev | -0.64 | -0.72 | 0.84 | -0.59 | 0.84 | 1.00 | -0.67 | -0.50 | 0.53 | 0.53 |
| Number of neighbors | 0.34 | 0.81 | -0.55 | 0.52 | -0.55 | -0.67 | 1.00 | 0.34 | -0.58 | -0.31 |
| Stddev of area represented by segments | 0.65 | 0.53 | -0.49 | 0.00 | -0.49 | -0.50 | 0.34 | 1.00 | -0.04 | -0.23 |
| Red band mean | -0.01 | -0.49 | 0.35 | -0.74 | 0.35 | 0.53 | -0.58 | -0.04 | 1.00 | 0.19 |
| GLCM ASM | -0.42 | -0.34 | 0.72 | -0.30 | 0.72 | 0.53 | -0.31 | -0.23 | 0.19 | 1.00 |

*Table 3. Accuracy assessment of classification results*

| **Classifier** | **Maximum likelihood(binary)** | **Maximum likelihood(multi)** | **CART（binary）** | **CART（multi）** | **CNN**  **(binary)** | **CNN**  **(multi)** | **SVM（binary）** | **SVM（multi）** |
| --- | --- | --- | --- | --- | --- | --- | --- | --- |
| Overall accuracy | 82.5% | 86.15% | 86.6% | 90.75% | 86.9% | 86.7% | 91.94% | 93.14% |
| Kappa coefficient | 0.60 | 0.67 | 0.66 | 0.76 | 0.64 | 0.65 | 0.77 | 0.81 |
| Producer accuracy (tea) | 93.35% | 93.28% | 86.60% | 88.02% | 72.5% | 76.66% | 79.87% | 83.81% |
| Producer accuracy (other vegetation) | 78.97% | 83.83% | 86.40% | 91.63% | 91.6% | 90.01% | 95.87% | 96.16% |
| User accuracy (tea) | 59.12% | 65.28% | 67.46% | 77.43% | 73.7% | 71.43% | 86.31% | 87.7% |
| User accuracy (other vegetation) | 97.32% | 97.45% | 95.18% | 95.91% | 91.1% | 92.2% | 93.6% | 94.81% |

* 11. Line 250. This defect can be largely eliminated by the use of LiDAR data.

Response **11**: Thanks for your suggestion. Since the LiDAR data can obtain canopy height and shape information, it can separate spectrally similar vegetation. However, airborne LiDAR data are hard to obtain in large area such as whole county. Meanwhile, space-borne LiDAR data such as ICESAT/GLAS, ICESAT-2 and GEDI have limited discrete coverage. The number of valid data in research area are quite limited, which makes space-borne LiDAR data hard to use in tea plantation extraction. In the future, we will be focus on multi-source data fusion method in order to use discrete covered space-borne LiDAR data to achieve better performance. We have added those as expectation in the paper (Line 401-402).

* 12. Line 261-262. I'm not fully convinced that this is the reason of the low accuracies of 2014 and 2015. I think it can be related to the features used, themselves. This is also true in the case of the other years.

Response 12: Thanks for your suggestion. Based on your suggestion, we have improved our feature space, adding more feature including spectrum, texture and geometry. The accuracies of 2014 and 2015 are improved. Experiments details and results have illustrated in the paper (Line210-249, Line 292-293).
