# Supplementary material for: Mapping Pu’er tea plantations from GF-1 images using Object-Oriented Image Analysis (OOIA) and Support Vector Machine (SVM)
Source: PLoS One. 2023 Feb 7;18(2):e0263969. doi: 10.1371/journal.pone.0263969 (PMC9904455; doi:10.1371/journal.pone.0263969)
Supplement: Attachment — Submitted filename: Manuscript-PONE-D-21-11402R2.docx [file pone.0263969.s005.docx]

**Manuscript Number:** PONE-D-21-11402R2
**Manuscript Title:** Mapping Pu'er Tea Plantations from GF-1 Images Using Object-Oriented Image Analysis (OOIA) and Support Vector Machine (SVM).

I would like to thank the authors for their answers. the following manuscript has been improved and can be accepted after a minor revision.

1. The authors should revise carefully the format of the manuscript; the size of titles is too large; the authors should follow the guidelines for authors. Also they can add the numbers for the titles and sub-titles in the manuscript.

2. The authors should revise all the tables especially tables 4 and 9.
