# Supplementary material for: Mapping Pu’er tea plantations from GF-1 images using Object-Oriented Image Analysis (OOIA) and Support Vector Machine (SVM)
Source: PLoS One. 2023 Feb 7;18(2):e0263969. doi: 10.1371/journal.pone.0263969 (PMC9904455; doi:10.1371/journal.pone.0263969)
Supplement: Attachment — Submitted filename: Response to Reviewer3.docx [file pone.0263969.s007.docx]

**Response to Reviewer #3 comments:**

Dear Editors and Reviewer:

Thank you for your comments concerning our manuscript entitled “Mapping Pu'er tea plantations from GF-1 images using OOIA and SVM” (ID: PONE-D-21-11402R1). Those comments are valuable and helpful for revising and improving our paper, as well as the important guiding significance to our researches. We have studied comments carefully and have made correction which we hope meet with approval. The responses to the reviewer’s comments are as follows:

* 1. The authors should revise carefully the format of the manuscript; the size of titles is too large; the authors should follow the guidelines for authors. Also they can add the numbers for the titles and sub-titles in the manuscript.

Response 1: Thanks for your suggestion. We have carefully revised the format of the manuscript. The large size of titles is because we havs to use the full name of the acronyms (CART and CNN), so it’s hard to shorten the title. Because the manuscript body formatting guidelines provided by PLOS do not have numbers in titles and sub-titles, we have to follow the guidelines and cannot add them in manuscript.

* 2. The authors should revise all the tables especially tables 4 and 9.

Response 2: Thanks for you remind. We have revised them.
