# Supplementary material for: Mapping Pu’er tea plantations from GF-1 images using Object-Oriented Image Analysis (OOIA) and Support Vector Machine (SVM)
Source: PLoS One. 2023 Feb 7;18(2):e0263969. doi: 10.1371/journal.pone.0263969 (PMC9904455; doi:10.1371/journal.pone.0263969)
Supplement: Attachment — Submitted filename: PONE-D-21-11402R3.docx [file pone.0263969.s008.docx]

**Manuscript ID:** PONE-D-21-11402R3

**Title:** Mapping Pu'er Tea Plantations from GF-1 Images Using Object-Oriented Image Analysis (OOIA) and Support Vector Machine (SVM)

PLOS ONE

I do not feel that this article is suitable for publishing in PLOS ONE Journal because the revision is so weak and the manuscript still has many gaps. Before the authors resubmit this manuscript to this journal or another journal please carefully consider my comments.

**Recommendations for Authors:**

1. In the abstract, the authors should add the manuscript's novelty and topicality.
2. The authors could rearrange the keywords alphabetically.
3. The authors should improve the introduction section, add recently published papers (after 2016), mention the gaps in previous studies and the innovations in this study and clarify the aims.
4. Weak methodology, please improve this part and clearly explain the methodology used in this study.
5. All the Figures are not clear, please plot your figure according to your results don’t take ready Figure from the output of program.
6. Change the Figure of the study area to a new figure that shows clearly the location of the study area.
7. Improve the methodology flowchart.
8. The authors should re-arrange all the Tables in the revised manuscript. Example like in Table 9 and 4 the authors could use abbreviation.
9. Please add more recently published papers in your revised manuscript. And keep the same format for all the references.
10. The authors should clearly discuss the results and compare them with previous work.
11. Future research statement should be stated in the conclusion section.
