# Supplementary material for: Mapping Pu’er tea plantations from GF-1 images using Object-Oriented Image Analysis (OOIA) and Support Vector Machine (SVM)
Source: PLoS One. 2023 Feb 7;18(2):e0263969. doi: 10.1371/journal.pone.0263969 (PMC9904455; doi:10.1371/journal.pone.0263969)
Supplement: Attachment — Submitted filename: Response to Reviewer3.docx [file pone.0263969.s009.docx]

**Response to Reviewer #3 comments:**

Dear Editors and Reviewer:

Thank you for your comments concerning our manuscript entitled “Mapping Pu'er tea plantations from GF-1 images using OOIA and SVM” (ID: PONE-D-21-11402R1). Those comments are valuable and helpful for revising and improving our paper, as well as the important guiding significance to our researches. We have studied comments carefully and have made correction which we hope meet with approval. The responses to the reviewer’s comments are as follows:

* 1. In the abstract, the authors should add the manuscript's novelty and topicality.

Response 1: Thanks for your suggestion. We have added the manuscript's novelty and topicality in abstract.

* 2. The authors could rearrange the keywords alphabetically.

Response 2: Thanks for you remind. We have rearranged the keywords.

* 3. The authors should improve the introduction section, add recently published papers (after 2016), mention the gaps in previous studies and the innovations in this study and clarify the aims.

Response 3: Thanks for your suggestion. We have added more related papers in introduction, for example, Wang B, Li J, Jin X, et al. Mapping Tea Plantations from Multi-seasonal Landsat-8 OLI Imageries Using a Random Forest Classifier. J Indian Soc Remote Sens. 2019; 47, 1315–1329; Fatemeh R, Mahdi K, Remote sensing-based detection of tea land losses: The case of Lahijan, Iran. Remote Sensing Applications: Society and Environment. 2021; 23: 568; Zhang Q, Wan B, Cao Z, Zhang Q. Exploring the Potential of Unmanned Aerial Vehicle (UAV) Remote Sensing for Mapping Plucking Area of Tea Plantations. Forests. 2021; 12(9):1214. We have added the gaps in previous studies and the innovations in this study.

* 4. Weak methodology, please improve this part and clearly explain the methodology used in this study.

Response 4: Thanks for your suggestion. We have improved the Research workflow part to better explain the methodology.

* 5. All the Figures are not clear, please plot your figure according to your results don’t take ready Figure from the output of program.

Response 5: Thanks for your suggestion. We have improved the clarity of all figures and re-plot the Fig 3 and Fig 12-13.

* 6. Change the Figure of the study area to a new figure that shows clearly the location of the study area.

Response 6: Thanks for your suggestion. We have re-plot the study area figure Fig 1.

* 7. Improve the methodology flowchart.

Response 7: Thanks for your suggestion. We have improved the methodology flowchart.

* 8. The authors should re-arrange all the Tables in the revised manuscript. Example like in Table 9 and 4 the authors could use abbreviation.

Response 8: Thanks for your suggestion. We have re-arranged tables in the manuscript.

* 9. Please add more recently published papers in your revised manuscript. And keep the same format for all the references.

Response 9: Thanks for your suggestion. We have added more recently published papers and the references.

* 10. The authors should clearly discuss the results and compare them with previous work.

Response 10: Thanks for you suggestion. We have improved the discussion of the results, and compared them with maximum likelihood, CART (Xu, 2016), CNN (Zixia, 2020).

* 11. Future research statement should be stated in the conclusion section.

Response 11: Thanks for your suggestion. We have added the future research statement in the expectation section.
